# Supplementary material for: Small-molecule-induced ERBB4 activation to treat heart failure
Source: Nat Commun. 2025 Jan 10;16:576. doi: 10.1038/s41467-024-54908-5 (PMC11724075; doi:10.1038/s41467-024-54908-5)
Supplement: Supplementary file 1 — Supplementary Information [file 41467_2024_54908_MOESM1_ESM.pdf]

Supplementary files

Small-Molecule-Induced ERBB4 Activation to Treat Heart Failure

Julie MT. Cools*[1], Bo K. Goovaerts*[1], Eline Feyen*[1], Siel Van den Bogaert[1], Yile Fu[2], Celine Civati[1], Jens Van fraeyenhove[1], Michiel R.L. Tubeeckx[1], Jasper Ott[3], Long Nguyen[4], Eike M. Wülfers[5,6], B. van Berlo[1], Antoine A.F. de Vries[7], Nele Vandersickel[5], Daniël A. Pijnappels[7], Dominique Audenaert[4], H. Llewelyn Roderick[2], Hans De Winter[8], Gilles W. De Keulenaer†[1,9], Vincent F.M. Segers†[1,10]

[1]Laboratory of PhysioPharmacology, University of Antwerp, Belgium

[2]Laboratory of Experimental Cardiology, Department of Cardiovascular Sciences, KU Leuven, Belgium

[3]Laboratory of Cell Biology and Histology, University of Antwerp, Belgium

[4a]Screening Core, VIB, Ghent, Belgium

[4b]Centre for Bioassay Development and Screening (C-BIOS), Ghent University, Belgium

[5]Department of Physics and Astronomy, Ghent University, Belgium

[6]Institute for Experimental Cardiovascular Medicine, University Heart Center Freiburg - Bad Krozingen, Freiburg im Breisbau, Germany and Faculty of Medicine, University of Freiburg, Freiburg im Breisgau, Germany

[7]Laboratory of Experimental Cardiology, Leiden University Medical Center, the Netherlands

[8]Laboratory of Medicinal Chemistry, University of Antwerp, Belgium

[9]Department of Cardiology, ZNA Hospital, Antwerp, Belgium

[10]Department of Cardiology, University Hospital Antwerp, Edegem, Belgium

*Contributed equally as first author.

†Contributed equally as last author.

Inventory

Supplementary files 1
Small-Molecule-Induced ERBB4 Activation to Treat Heart Failure 1
Inventory 2
Supplementary Table 1 3
Supplementary Figure 1 6
Supplementary Figure 2 7
Supplementary Figure 3 8
Supplementary Figure 4 9
Supplementary Figure 5 11
Supplementary Table 2 12
Supplementary Figure 6 15
Supplementary Figure 7 17
Supplementary Figure 8 18
Supplementary Figure 9 19

Supplementary Table 1

Category	Parameter	Description
Assay	Type of assay	PathHunter Dimerization assays provide a robust, highly sensitive and easy-to-use cell-based functional assay to study various protein activities in a cell. The assays use Enzyme Fragment Complementation technology, where the b-galactosidase (b-gal) enzyme is split into two fragments, ProLink and Enzyme Acceptor. Independently these fragments have no b-gal activity. However, when forced to complement through protein-protein interactions, they form an active b-gal enzyme. The PathHunter Dimerization assay detects ligand induced dimerization of two subunits of a receptor-dimer pair. The cells have been engineered to co-express one receptor subunit (ERBB4) fused to Enzyme Donor, and a second dimer partner (ERBB4) fused to Enzyme Acceptor. Binding of an agonist to one receptor subunit induces it to interact with its dimer partner, forcing complementation of the two enzyme fragments. This results in the formation of a functional enzyme that hydrolyzes a substrate to generate a chemiluminescent signal.
	Target	ErbB4 receptor (RTK receptor)
	Primary measurement	Detection of chemiluminescent signal, induced by small-molecule ErbB4 agonists
	Key reagents	PathHunter Dimerization ErbB4/ErbB4 Cell Line (93-0961C3) - PathHunter Flash Detection Kit (03-0247) - AssayComplete Cell Culture Kit-103 (92-3103G) - AssayComplete Cell Plating 0 Reagent (93-0563R0A) - AssayComplete Cell Detachment Reagent (92-0009) - AssayComplete Thawing Reagent T3 (92-4103TR) - AssayComplete Freezing Reagent F3 (92-5103FR)
	Assay protocol	https://www.discoverx.com/catalog/pathhunter-u2os-erbb4-erbb4-dimerization-cell-line/93-0961c3
	Additional comments	/
Library	Library size	10,240 synthetic molecules
	Library composition	Pharmacological Diversity Set
	Source	Enamine
	Additional comments	/
Screen	Format	384-well plate format, 5,000 cells/well
	Concentration(s) tested	Compounds at 10 µM final concentration; in all wells, finale DMSO concentration is 1%

	Plate controls	Negative control: PBS<br>Positive control: NRG1
	Reagent/compound dispensing system	PBS
	Detection instrument and software	Read-out with EnVision plate reader (PerkinElmer) for luminescence measurements
	Assay validation/QC	Z' score is 0.81. Eight different concentrations of NRG1 were used to validate the assay, n=3 biological replicates.
	Correction factors	Background values (vehicle) are subtracted from all values as correction.
	Normalization	Intraplate normalization via two-way median polish to correct for row, column and edge effects. The input are raw value measurements, the output are normalized values for each compound (= residuals). Interpolate normalization via B-score calculation to allow comparison between plates. The input are residuals and the output are relative potencies (= B-scores).
	Additional comments	The screening was done at the VIB Screening Core and the Ghent University Centre for Bioassay Development and Screening (C-BIOS)
Post-HTS analysis	Hit criteria	B-score > 0 implies a higher signal than the average of the samples and B-score < 0 implies a lower signal than the average of the samples. We made a B-score ranking from high to low corresponds with potency ranking from high to low. The top 80 compounds with the highest B-scores from the primary screen were selected for confirmation screen.
	Hit rate	62/10,240
	Additional assay(s)	A confirmation screen was done with the same procedure as the primary screen. The threshold for confirmed hits was a luminescence value that is higher than the average of the negative control plus three times the standard deviation of the negative control. This resulted in 62 confirmed hits.
	Confirmation of hit purity and structure	The hit compounds were repurchased (Enamine) and the structure and purity was verified analytically and confirmed by Enamine after resynthesis.
	Additional comments	/

Supplementary Table 1. Small molecule screening data. Source data are provided as a Source Data file. B-gal, b-galactosidase; C-BIOS, Centre for Bioassay Development and Screening; DMSO, dimethyl

sulfoxide; ErbB4, Erythroblastic leukemia viral oncogene homolog 4; HTS, high-throughput screening; NRG1, neuregulin-1; PBS, phosphate buffered saline; QC, quality control; RTK, receptor tyrosine kinase.

Supplementary Figure 1

a

#	Smarts	Hitlist count	Reference count	Enrichment
0	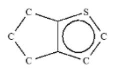	4	5,434	**21**
1	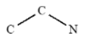	54	1,616,180	**1.0**
2	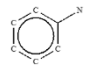	24	712,585	**1.0**
3	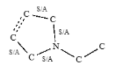	2	26,295	**2.2**
4	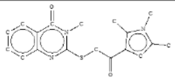	2	127	**458**
5	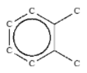	3	118,392	**0.7**
6	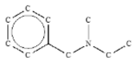	6	197,723	**0.9**
7	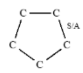	9	65,699	**4.0**
8	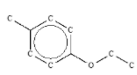	4	76,764	**1.5**
9	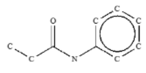	4	142,189	**0.8**

b

MCS	Hitlist count	Reference count	Enrichment
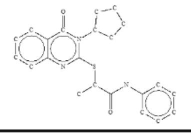	2	6	9,692
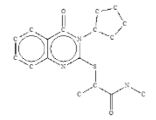	3	16	5,452
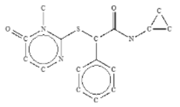	2	19	3,060

Resulting MCS:
Count in hitlist: 4 / 62
Count in reference set: 1,070 / 1,773,567
Enrichment: 109

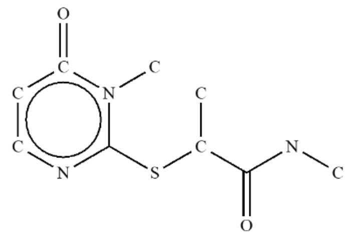

Supplementary Figure 1. Pharmacophore identification. Enrichment analysis of the identified MCS cluster centers and substructure pharmacophore identification. **(a)** Enrichment analysis on the 10 identified MCS cluster centers of the 62 confirmed hits. 'Hitlist counts' represents the number of hits in which the particular MCS is found, while the corresponding 'Reference count' represents the number of reference molecules (the Enamine HTS library containing 1,773,567 molecules) in which the same MCS is found. The 'Enrichment' equals the 'Hitlist count' divided by the 'Reference count'. **(b)** Three MCS patterns within cluster center number 4 give a particular high enrichment. The common MCS derived from these 3 individual MCSs (shown in the bottom right) gives an enrichment of 109, and this MCS pattern was used for subsequent substructure searches. HTS, high-throughput screening; MCS, maximum common substructure; smarts, smiles arbitrary target specification or substructural patterns in molecules.

Supplementary Figure 2

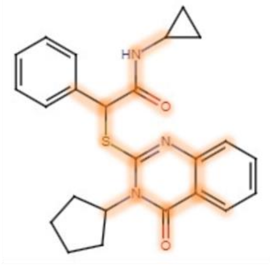

C24H25N3O2S, MW: 419.5
EF-1

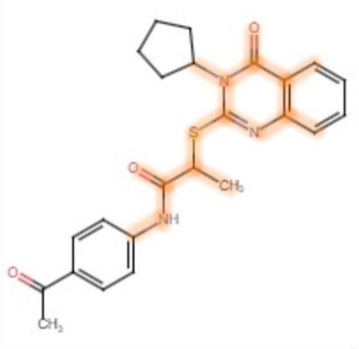

C24H25N3O3S, MW: 435.5
EF-2

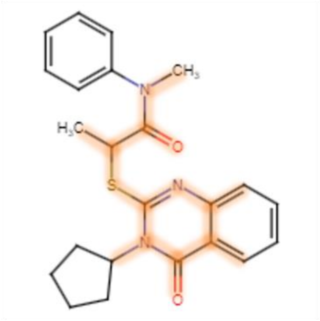

C23H25N3O2S, MW: 407.5
EF-3

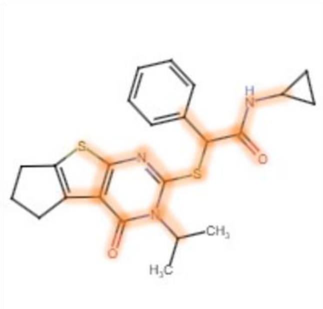

C23H25N3O2S2, MW: 439.6
EF-4

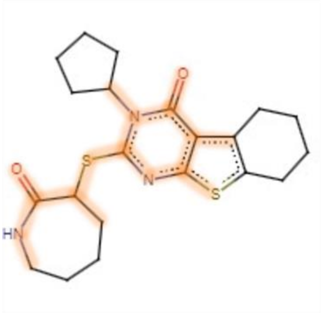

C21H27N3O2S2, MW: 417.6
EF-5

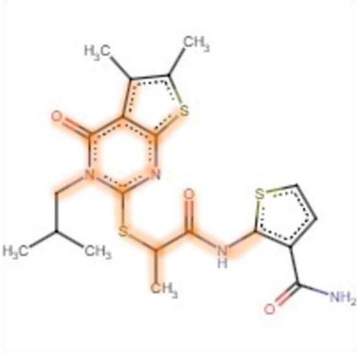

C20H24N4O3S3, MW: 464.6
EF-6

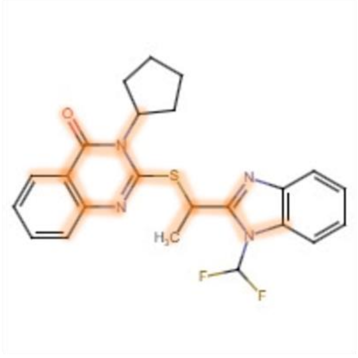

C23H22F2N4OS, MW: 440.5
EF-7

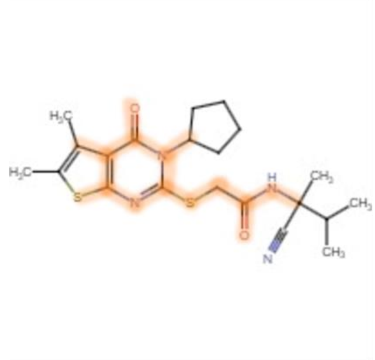

C21H28N4O2S2, MW: 417.6
EF-8

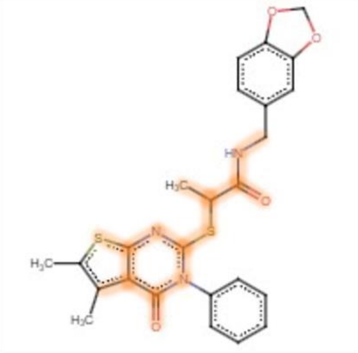

C25H23N3O4S2, MW: 493.6
NA-1

Supplementary Figure 2. Chemical structure and MW of the eight selected hit compounds. The common pharmacophore is highlighted in orange. MW, molecular weight; NA, non-active compound containing pharmacophore.

Supplementary Figure 3

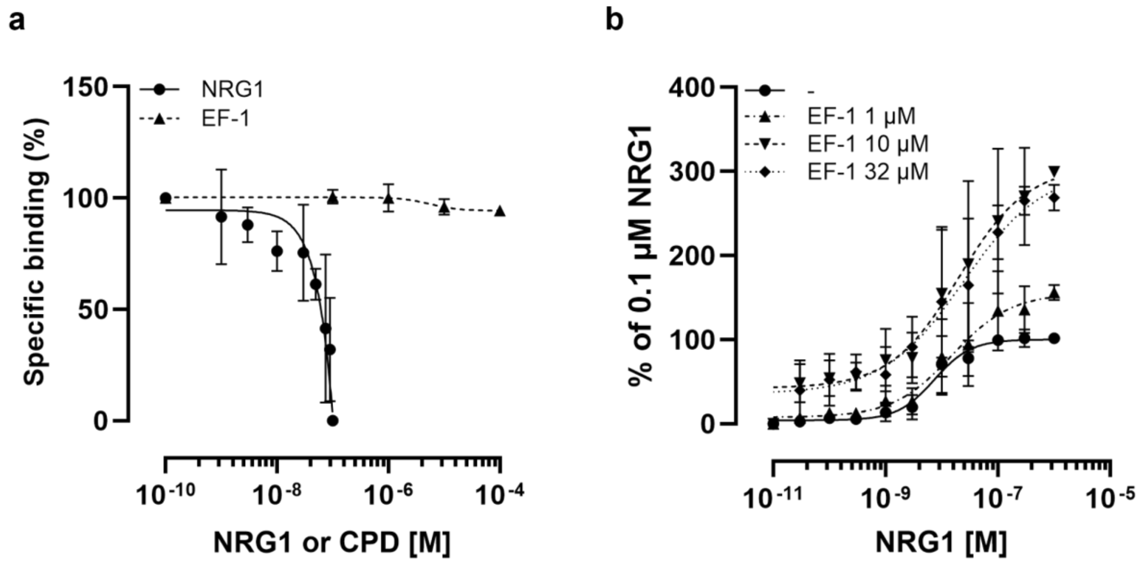

Supplementary Figure 3. Receptor binding assay. (a) Fluorescence-based competition assay between 30 nM F-NRG1 and different concentrations of unlabeled NRG1 or EF-1. The Y-axis represents the percentage of specific binding of F-NRG1, the x-axis represents the molar concentration of unlabeled NRG-1 (full line with full circle) or EF-1 (dashed line with triangle) that was added (n=3 biological replicates for $9x10^{-8}$ M NRG1, n=4 for $1x10^{-8}$ M and $5x10^{-8}$ M NRG1, n=6 for $1x10^{-9}$ M and $3x10^{-9}$ M NRG1, n=8 for $7.5x10^{-8}$ M NRG1, n=10 for $1x10^{-10}$ M, $3x10^{-8}$ M NRG1 and $1x10^{-7}$ M, n=3 for every concentration of EF-1). **(b)** DRC showing the effect of NRG1 (full line with full circle) on the ERBB4/ERBB4 dimerization assay in the presence of 3 different concentrations of EF-1 (1 µM EF-1 = dashed line with triangle: 10 µM EF-1 = dashed line with inverted triangle; 32 µM EF-1 = dashed line with diamond; n=9 biological replicates for NRG1 without EF-1 for all concentrations of NRG1, except n=8 for $3x10^{-11}$ M and $1x10^{-10}$ M NRG1, n=7 for $3x10^{-7}$ M NRG1 and n=4 for $1x10^{-6}$ M NRG1; n=6 biological replicates for NRG1 + EF-1 1 µM for all concentrations of NRG1, except n=4 for $3x10^{-7}$ M and $1x10^{-6}$ M NRG1; n=5 biological replicates for NRG1 + EF-1 10 µM for all concentrations of NRG1, except n=4 for $3x10^{-7}$ M and n=1 for $1x10^{-6}$ M NRG1; n=5 biological replicates for NRG1 + EF-1 32 µM for all concentrations of NRG1, except n=2 for $3x10^{-7}$ M and $1x10^{-6}$ M NRG1). All data are presented as mean ± SD. Source data are provided as a Source Data file. CPD, compound; DRC; dose response curve; FC, fold change; F-NRG1, fluorescently labeled NRG1; M, molar concentration; NRG1, neuregulin-1; Veh, vehicle.

Supplementary Figure 4

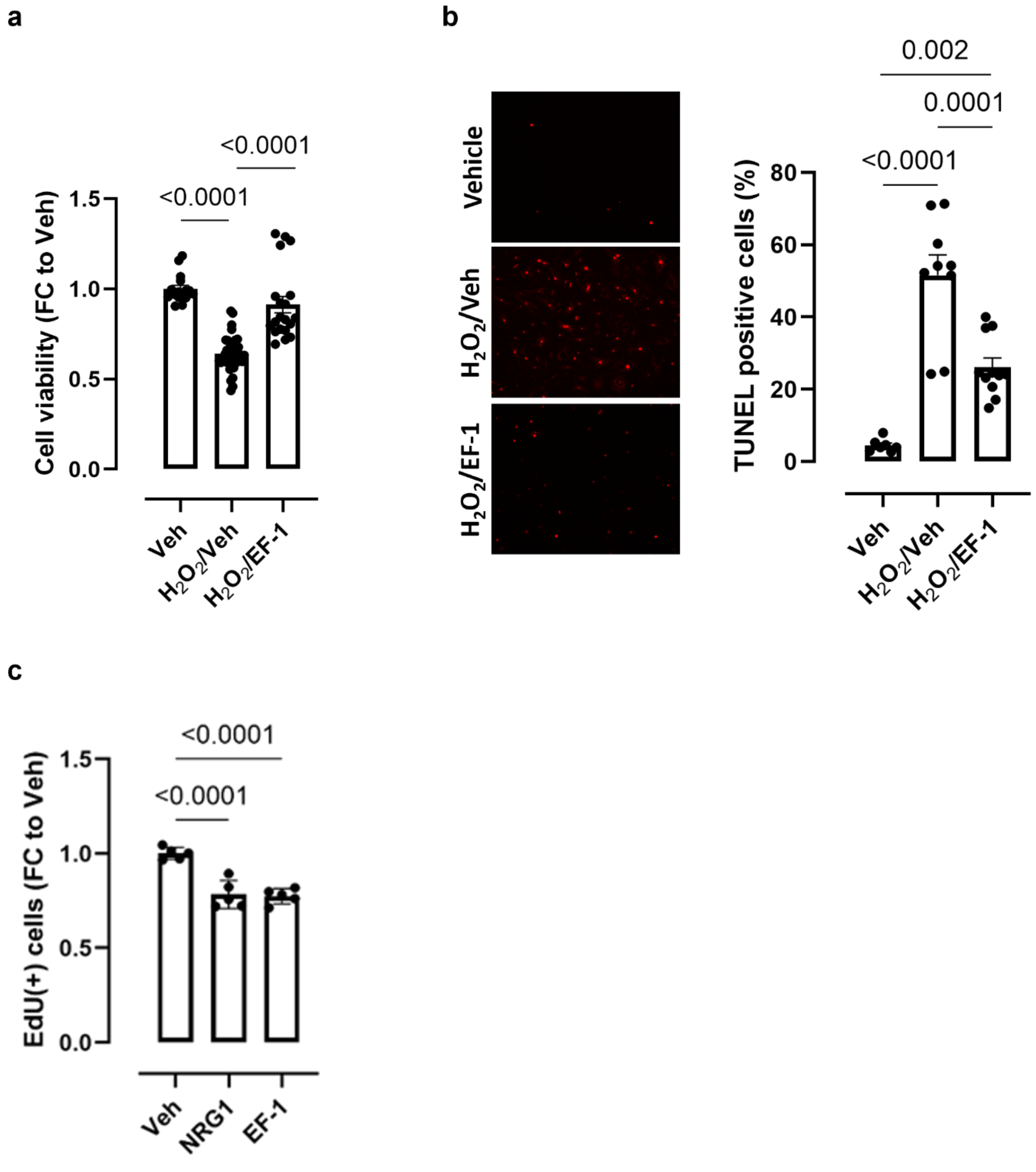

Supplementary Figure 4. EF-1 decreases H_2O_2-induced cardiomyocyte cell death and has no and proliferation properties. (a) Effect of EF-1 on cell viability assessed with the WST-1 assay (n=4 technical replicates in 4 biological replicates in Veh group, n=10 technical replicates in 4 biological replicates for H_2O_2/Veh group, n=5 technical replicates in 4 biological replicates for H_2O_2/EF-1 group). **(b)** Effect of EF-1 on TUNEL staining (red). The corresponding bar graph shows the average percentage of TUNEL-positive cells (n=7 biological replicates in Veh group, n=9 biological replicates for H_2O_2/Veh group, n=11 biological replicates for H_2O_2/EF-1 group). **(c)** EdU-positive cells (FC to Veh) in iAMs, stimulated with EF-

1 or NRG1 (n=5 biological replicates in each group). All data are represented as mean ± SD, one-way ANOVA with Tukey's multiple comparisons test. Source data are provided as a Source Data file. ANOVA, analysis of variance; EdU, 5-Ethynyl-2'-deoxyuridine; FC, fold change; H_2O_2, hydrogen peroxide; iAM, conditionally immortalized rat atrial myocyte; NRG1, neuregulin-1; TUNEL, terminal deoxynucleotidyl transferase dUTP nick end labelling; Veh, vehicle; WST-1, 2-(4-iodophenyl)-3-(4-nitrophenyl)-5-(2,4-disulfophenyl)-2H-tetrazolium.

Supplementary Figure 5

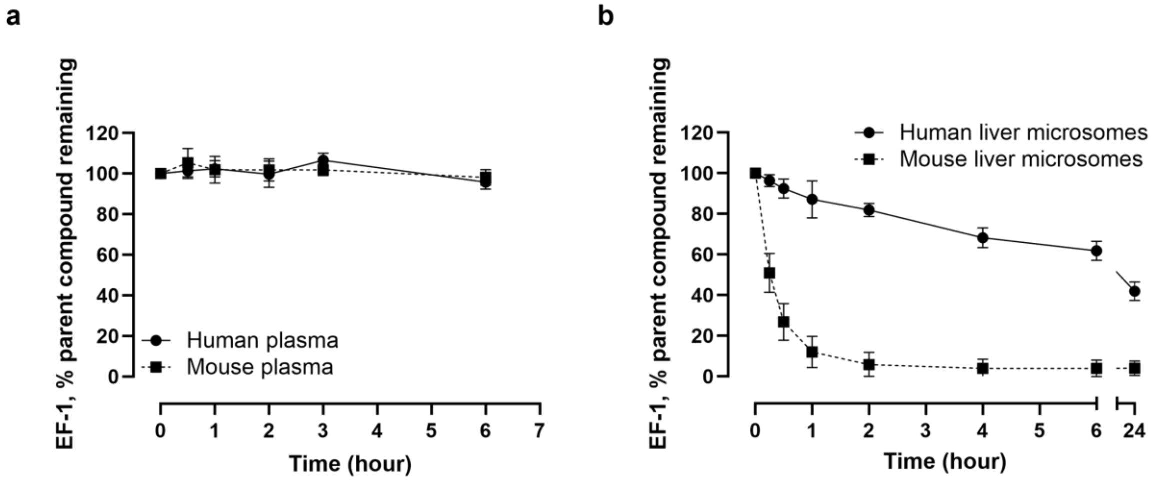

Supplementary Figure 5. Stability of EF-1 in plasma and liver microsomes. (a) Stability of EF-1 in plasma from human (full line with full circle) and mouse (dashed line with square; n=3 technical replicates in 2 biological replicates for both human and mouse plasma for every time point, except n=2 technical replicates once for time point “3 hours” for both human and mouse plasma) and **(b)** in liver microsomes isolated from human (full line with full circle) and mouse (dashed line with square; n=3 technical replicates in 3 biological replicates for both human and mouse liver microsomes for every time point, except n=2 biological replicates for time point “2 hours” for human liver microsomes). Source data are provided as a Source Data file.

Supplementary Table 2

a)

	Male wild-type mice treated with AngII and EF-1			
	**Ctrl**	**EF-1**	**AngII/Veh**	**AngII/EF-1**
**IVSd (mm)**	0.9 ± 0.2	0.9 ± 0.1	0.8 ± 0.2	0.8 ± 0.1
**IVSs (mm)**	1.1 ± 0.2	1.2 ± 0.1	1.0 ± 0.1	1.1 ± 0.2
**LVIDd (mm)**	4.3 ± 0.4	4.2 ± 0.3	4.3 ± 0.3	4.4 ± 0.3
**LVIDs (mm)**	3.5 ± 0.4	3.4 ± 0.3	3.7 ± 0.4	3.7 ± 0.4
**LVPWd (mm)**	0.7 ± 0.1	0.75 ± 0.03	0.8 ± 0.1	0.8 ± 0.2
**LVPWs (mm)**	0.9 ± 0.1	0.9 ± 0.1	0.9 ± 0.2	0.9 ± 0.2
**FS (%)**	18.0 ± 2.9	18.5 ± 5.1	14.1 ± 4.0	16.6 ± 3.6

b)

	Female wild-type mice treated with AngII and EF-1		
	**Ctrl**	**AngII/Veh**	**AngII/EF-1**
**IVSd (mm)**	0.51 ± 0.08	0.5 ± 0.1	0.53 ± 0.07
**IVSs (mm)**	0.7 ± 0.1	0.7 ± 0.2	0.7 ± 0.1
**LVIDd (mm)**	4.6 ± 0.4	4.6 ± 0.3	4.2 ± 0.3
**LVIDs (mm)**	3.9 ± 0.6	4.0 ± 0.3	3.7 ± 0.4
**LVPWd (mm)**	0.49 ± 0.08	0.5 ± 0.1	0.5 ± 0.1
**LVPWs (mm)**	0.7 ± 0.1	0.6 ± 0.1	0.6 ± 0.1
**FS (%)**	15.7 ± 7.4	11.7 ± 6.2	11.5 ± 5.5

c)

	Male wild-type mice treated with AngII and NA-1		
	**Ctrl**	**AngII/Veh**	**AngII/NA-1**
**IVSd (mm)**	0.8 ± 0.1	1.0 ± 0.2	1.0 ± 0.2
**IVSs (mm)**	1.1 ± 0.1	1.3 ± 0.3	1.4 ± 0.2
**LVIDd (mm)**	4.1 ± 0.4	4.3 ± 0.5	4.3 ± 0.7
**LVIDs (mm)**	3.4 ± 0.4	3.5 ± 0.3	3.5 ± 0.7
**LVPWd (mm)**	0.8 ± 0.3	1.2 ± 0.4	0.9 ± 0.1
**LVPWs (mm)**	1.0 ± 0.3	1.4 ± 0.4	1.1 ± 0.3
**FS (%)**	18.2 ± 2.0	18.8 ± 5.0	18.1 ± 5.1

d)

	Validation male and female *Erbb4-null* mice		
	**Wild-type mice**	**Erbb4-null mice**	**P-value**
**LVIDd (mm)**	4.2 ± 0.4	5.0 ± 0.5	0.003
**LVIDs (mm)**	3.5 ± 0.4	4.6 ± 0.6	0.0006
**FS (%)**	18.1 ± 2.3	9.4 ± 6.1	0.001

e)

	Male and female *Erbb4-null* mice treated with AngII and EF-1			
	**Ctrl**	**AngII/Veh**	**AngII/EF-1**	**P-value**
**IVSd (mm)**	0.9 ± 0.2	0.8 ± 0.2	1.0 ± 0.3	/
**IVSs (mm)**	1.1 ± 0.2	0.9 ± 0.3	1.2 ± 0.5	/
**LVIDd (mm)**	5.0 ± 0.5	5.5 ± 0.5	5.8 ± 0.3	‡0.04
**LVIDs (mm)**	4.6 ± 0.6	5.0 ± 0.7	5.5 ± 0.5	/
**LVPWd (mm)**	0.7 ± 0.1	0.8 ± 0.1	1.0 ± 0.4	‡0.05
**LVPWs (mm)**	0.7 ± 0.1	0.9 ± 0.1	1.1 ± 0.4	/
**FS (%)**	9.4 ± 6.1	8.3 ± 5.6	5.8 ± 3.5	/

f)

	Myocardial infarction in female wild-type mice treated with EF-1			
	**Ctrl**	**MI/Veh**	**MI/EF-1**	**P-value**
	**T0**			
**LVEDA (mm²)**	21.3 ± 2.4	22.2 ± 2.8	21.8 ± 1.4	/

**LVESA (mm²)**	14.8 ± 1.5	14.5 ± 1.4	15.5 ± 2.1	/
**LVEDV (µL)**	51.9 ± 7.9	57.1 ± 11.5	56.7 ± 5.5	/
**LVESV (µL)**	31.2 ± 6.2	27.9 ± 4.3	32.7 ± 6.7	/
**EF (%)**	42.7 ± 10.1	49.5 ± 11.5	42.7 ± 8.1	/
	**T2**			
**LVEDA (mm²)**	23.2 ± 2.1	27.2 ± 4.8	25.7 ± 1.6	¥0.04
**LVESA (mm²)**	16.1 ± 1.7	22.3 ± 5.8	20.8 ± 2.9	¥0.01; ‡0.05
**LVEDV (µL)**	63.6 ± 9.9	83.8 ± 24.0	73.6 ± 9.1	¥0.04
**LVESV (µL)**	34.9 ± 7.0	62.0 ± 28.0	53.9 ± 14.5	¥0.01
**EF (%)**	44.6 ± 5.0	29.0 ± 14.8	27.4 ± 14.1	¥0.05; ‡0.02
	**T3**			
**LVEDA (mm²)**	21.6 ± 2.1	31.5 ± 6.0	26.5 ± 4.5	¥0.0005
**LVESA (mm²)**	15.5 ± 2.1	28.0 ± 6.6	21.2 ± 5.8	¥0.0003; †0.03
**LVEDV (µL)**	55.1 ± 7.7	107.5 ± 29.1	80.3 ± 22.9	¥0.0002; †0.04
**LVESV (µL)**	31.1 ± 7.6	86.5 ± 32.3	56.6 ± 25.0	¥0.0002; †0.04
**EF (%)**	42.8.3 ± 6.3	21.5 ± 9.4	32.2 ± 12.4	¥0.0007
	**T4**			
**LVEDA (mm²)**	21.6 ± 2.1	31.5 ± 6.0	26.5 ± 4.5	¥0.0005
**LVESA (mm²)**	15.5 ± 2.1	28.0 ± 6.6	21.2 ± 5.8	¥0.0003; †0.03
**LVEDV (µL)**	55.1 ± 7.7	107.5 ± 29.1	80.3 ± 22.9	¥0.0002; †0.04
**LVESV (µL)**	31.1 ± 7.6	86.5 ± 32.3	56.6 ± 25.0	¥0.0002; †0.04
**EF (%)**	42.8 ± 6.3	21.5 ± 9.4	32.2 ± 12.4	¥0.0007

**g)**	**Myocardial infarction in male wild-type mice treated with EF-1**			
	**Ctrl**	**MI/Veh**	**MI/EF-1**	**P-value**
	**T0**			
**LVEDA (mm²)**	24.1 ± 2.8	25.7 ± 2.3	24.7 ± 3.9	/
**LVESA (mm²)**	17.7 ± 2.6	18.4 ± 3.1	17.6 ± 2.8	/
**LVEDV (µL)**	63.0 ± 11.6	71.4 ± 13.2	67.1 ± 15.6	/
**LVESV (µL)**	40.0 ± 9.9	40.5 ± 13.1	37.3 ± 8.9	/
**EF (%)**	41.2 ± 8.8	43.5 ± 11.1	43.6 ± 9.5	/
	**T2**			
**LVEDA (mm²)**	26.2 ± 2.1	34.8 ± 5.1	34.8 ± 5.5	¥0.0007; ‡0.001
**LVESA (mm²)**	18.9 ± 2.2	28.9 ± 5.2	29.7 ± 6.7	¥0.0004; ‡0.0003
**LVEDV (µL)**	72.7 ± 8.8	117.7 ± 28.6	118.7 ± 30.8	¥0.001; ‡0.001
**LVESV (µL)**	41.9 ± 7.5	89.0 ± 27.3	93.3 ± 36.1	¥0.001; ‡0.0008
**EF (%)**	42.2 ± 7.7	24.9 ± 11.2	23.2 ± 12.8	¥0.003; ‡0.002
	**T3**			
**LVEDA (mm²)**	23.4 ± 2.2	31.2 ± 4.0	36.6 ± 5.9	¥0.02; ‡0.0008
**LVESA (mm²)**	15.5 ± 2.7	25.0 ± 5.7	31.3 ± 8.0	¥0.05; ‡0.003
**LVEDV (µL)**	61.2 ± 7.6	95.1 ± 17.1	124.6 ± 35.3	‡0.002
**LVESV (µL)**	30.2 ± 7.3	67.9 ± 26.2	100.4 ± 42.0	‡0.005
**EF (%)**	50.5 ± 10.2	30.7 ± 15.2	22.0 ± 15.7	‡0.02
	**T4**			
**LVEDA (mm²)**	26.3 ± 2.5	33.2 ± 6.1	35.9 ± 6.9	¥0.02; ‡0.002
**LVESA (mm²)**	18.4 ± 2.3	26.2 ± 3.7	30.5 ± 7.7	¥0.006; ‡<0.0001
**LVEDV (µL)**	72.7 ± 12.4	108.8 ± 36.8	119.8 ± 37.5	¥0.04; ‡0.009
**LVESV (µL)**	42.3 ± 9.9	72.3 ± 18.5	92.5 ± 40.6	¥0.05; ‡0.009

**EF (%)**	42.0 ± 7.2	26.0 ± 10.5	25.2 ± 11.5	¥0.003; ‡0.003

Supplementary Table 2. Echocardiographic parameters. Echocardiographic parameters obtained through the analysis of the M-mode short-axis views. **(a)** Male and **(b)** female wild-type mice treated with AngII and EF-1 (n=5 male and 8 female mice in Ctrl, AngII/Veh and AngII/EF-1 group, n=4 male mice in EF-1 group). **(c)** Male wild-type mice treated with AngII and NA-1 (n=5 male mice in Ctrl and AngII/Veh group, n=4 male mice in AngII/NA-1 group). **(d)** Comparison between wild-type and *Erbb4-null* mice (n=10 male and female wild-type mice and n=6 male and female *Erbb4-null* mice). **(e)** Male and female *Erbb4-null* mice treated with AngII and EF-1 (n=6 male and female *Erbb4-null* mice in Ctrl group and n=5 male and female *Erbb4-null* mice in AngII/Veh and AngII/EF-1 group). **(f)** Echocardiographic parameters obtained through left ventricular trace analysis of the parasternal long-axis obtained in B-mode in female wild-type mice that underwent a sham or MI operation: mice with the MI were treated with Veh or EF-1 (n=8 female mice in Ctrl and MI/Veh group and n=9 female mice in MI/EF-1 group). **(g)** Echocardiographic parameters obtained through left ventricular trace analysis of the parasternal long-axis obtained in B-mode in male wild-type mice that underwent a sham or MI operation: mice with the MI were treated with Veh or EF-1 (n=5 male mice in Ctrl and MI/EF-1 group and n=6 male mice in MI/Veh group for T0 and T3; n=10 male mice in Ctrl and MI/EF-1 group and n=12 male mice in MI/Veh group for T2 and T4). Echocardiographic parameters were measured weekly (indicated by 'T'). All data are presented as mean (value of individual mice) ± SD, two-tailed unpaired t-test or one-way ANOVA test with Tukey's correction for multiple testing. ¥ shows significant *P* -values between **(f-g)** ctrl and MI/Veh groups; ‡ shows significant *P*-values between **(e)** ctrl and AngII/EF-1 groups and **(f-g)** ctrl and MI/EF-1 groups; † shows significant *P* -values between **(f-g)** MI/Veh and MI/EF-1 groups. Source data are provided as a Source Data file. AngII, angiotensin II; ANOVA, analysis of variance; Ctrl, control; DOX, doxorubicin; EF, ejection fraction; ERBB4, erythroblastic leukemia viral oncogene homolog 4; FS, fractional shortening; IVSd, interventricular septum thickness in diastole; IVSs, interventricular septum thickness in systole; LVIDd, left ventricular internal diameter in diastole; LVIDs, left ventricular internal diameter in systole; LVPWd, left ventricular posterior wall thickness in diastole; LVPWs, left ventricular posterior wall thickness in systole; MI, myocardial infarction; NA, non-activating pharmacophore-containing compound; SD, standard variation; Veh, vehicle.

Supplementary Figure 6

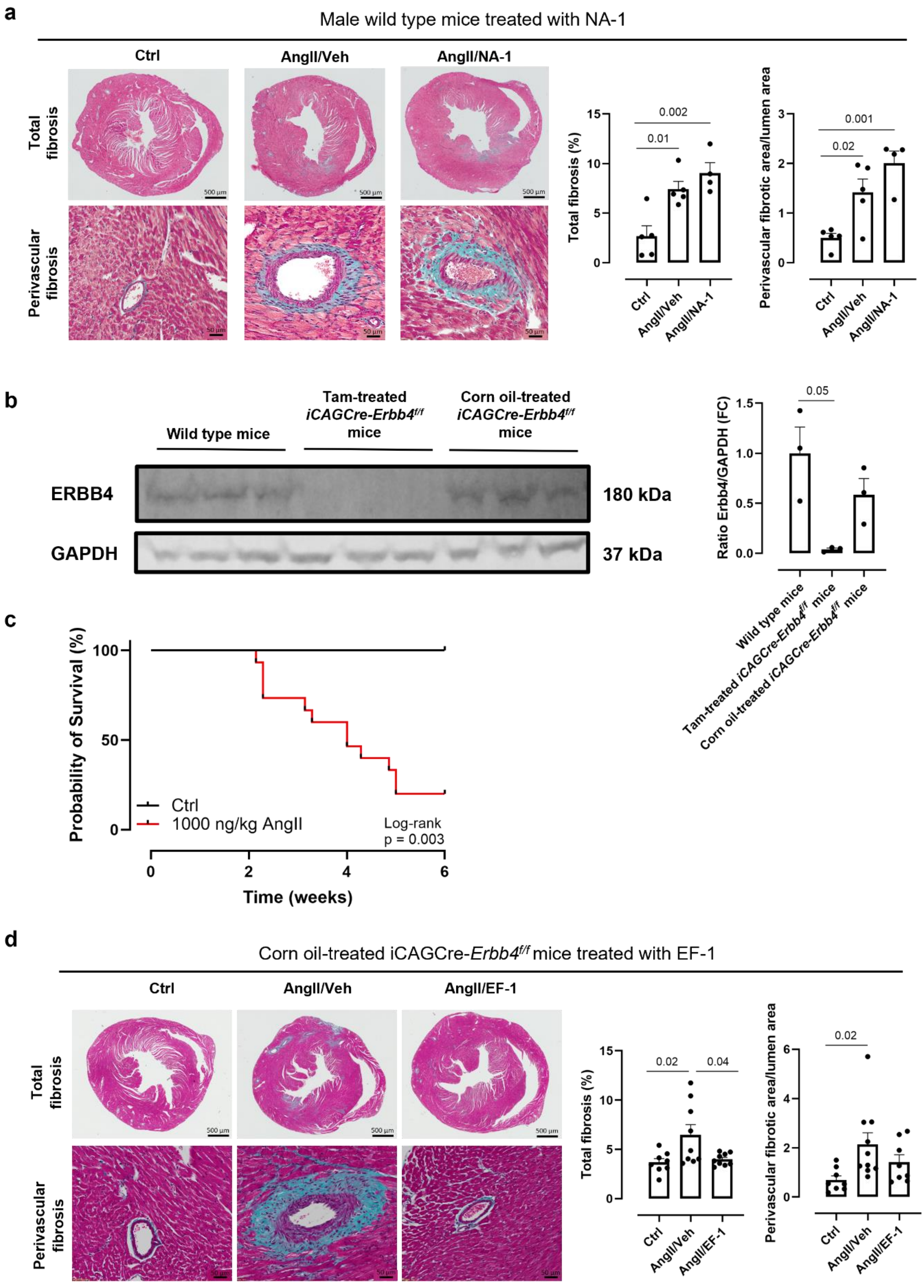

Supplementary Figure 6. Effect of AngII in male WT mice with compound NA-1 and the validation of genotype and phenotype of iCAGCre-Erbb4 f/f mice. (a) Representative images of Masson's trichrome staining of AngII-induced myocardial fibrosis following treatment with NA-1 and corresponding graphs showing the quantitation for total and perivascular fibrosis in male mice (n=5 male mice in Ctrl and AngII/Veh group, n=4 male mice in AngII/NA-1 group). **(b)** Representative western blot image and corresponding graph of the validation of the presence of Erbb4 in wild type mice and in i*CAGCre-Erbb4*$^{f/f}$ mice treated with tamoxifen or corn oil. The graph bar represent mean values of ErbB4 normalized to the housekeeping protein GAPDH, relative to wild type mice (n=3 individual mice for each group). Uncropped gel is presented in Supplementary Figure 9. **(c)** Survival curve depicting the effects of 1000 ng/kg AngII treatment on mouse survival (ctrl = black line; 1000 ng/kg AngII = red line; n=6 male and female mice in Ctrl group and n=15 mice who received 1000 ng/kg AngII treatment). **(d)** Representative images of Masson's trichrome staining of AngII-induced myocardial fibrosis in i*CAGCre-Erbb4*$^{f/f}$ mice injected with corn oil, treated with EF-1 or Veh and corresponding graphs showing the quantitation for total and perivascular fibrosis (n=8 male and female mice in Ctrl and AngII/EF-1 group and n=10 male and female ice in AngII/Veh group). Scale bar = 500 µm for total fibrosis, scale bar = 50 µm for perivascular fibrosis. All data are represented as mean ± SD, one-way ANOVA with Tukey's multiple comparisons test. For the survival curve analysis, a Kaplan-Meier test was done with the log-rank (Mantel-Cox) test. Source data are provided as a Source Data file. AngII, angiotensin II; ANOVA, analysis of variance; Ctrl, control; ERBB4, erythroblastic leukemia viral oncogene homolog 4; GAPDH, glyceraldehyde-3-phosphate dehydrogenase; SD, standard deviation; Tam, tamoxifen; Veh, vehicle.

Supplementary Figure 7

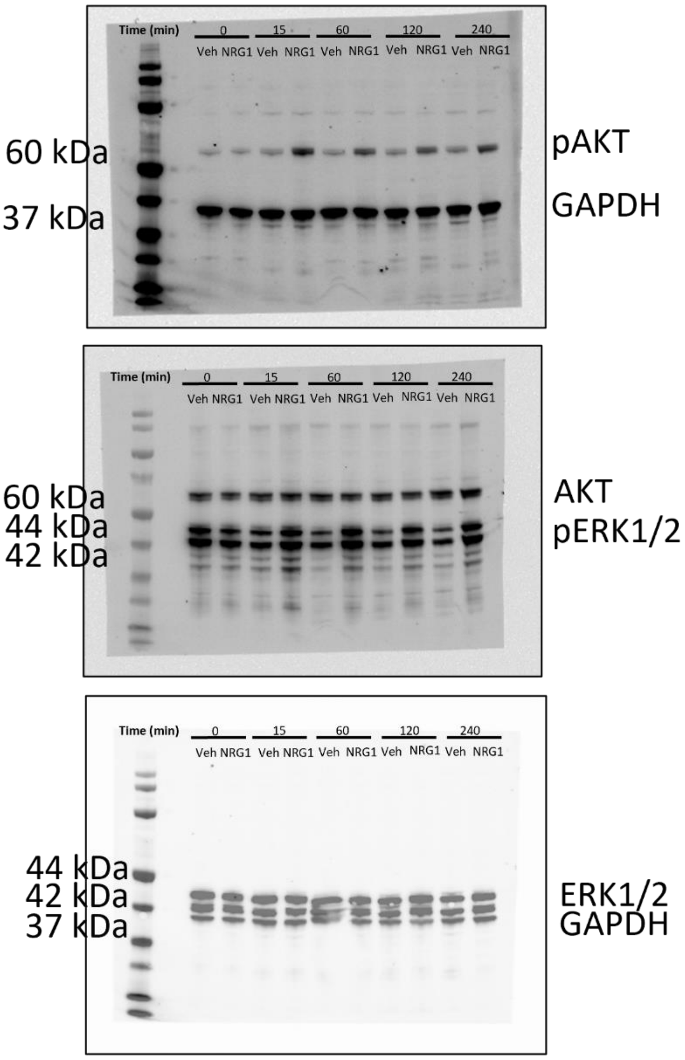

Supplementary Figure 7. Uncropped gels corresponding to western blots presented in **Fig. 2a**.

Supplementary Figure 8

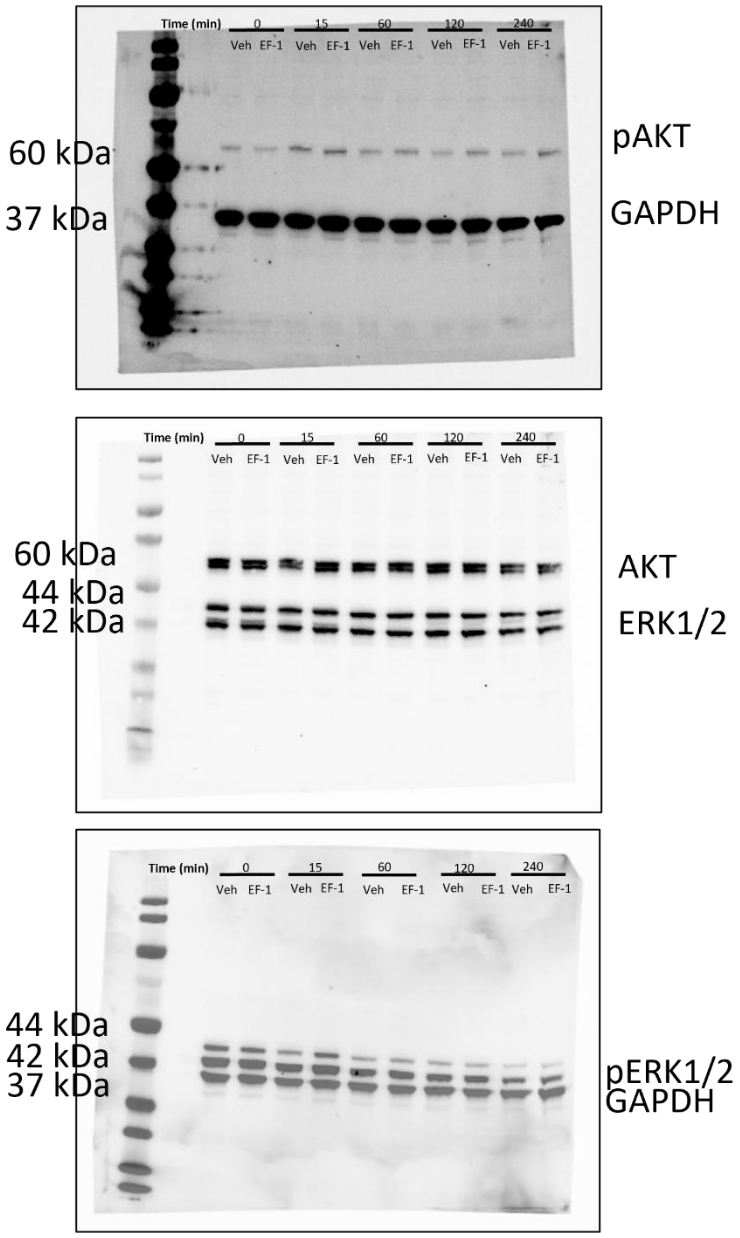

Supplementary Figure 8. Uncropped gels corresponding to western blots presented in **Fig. 2b**.

Supplementary Figure 9

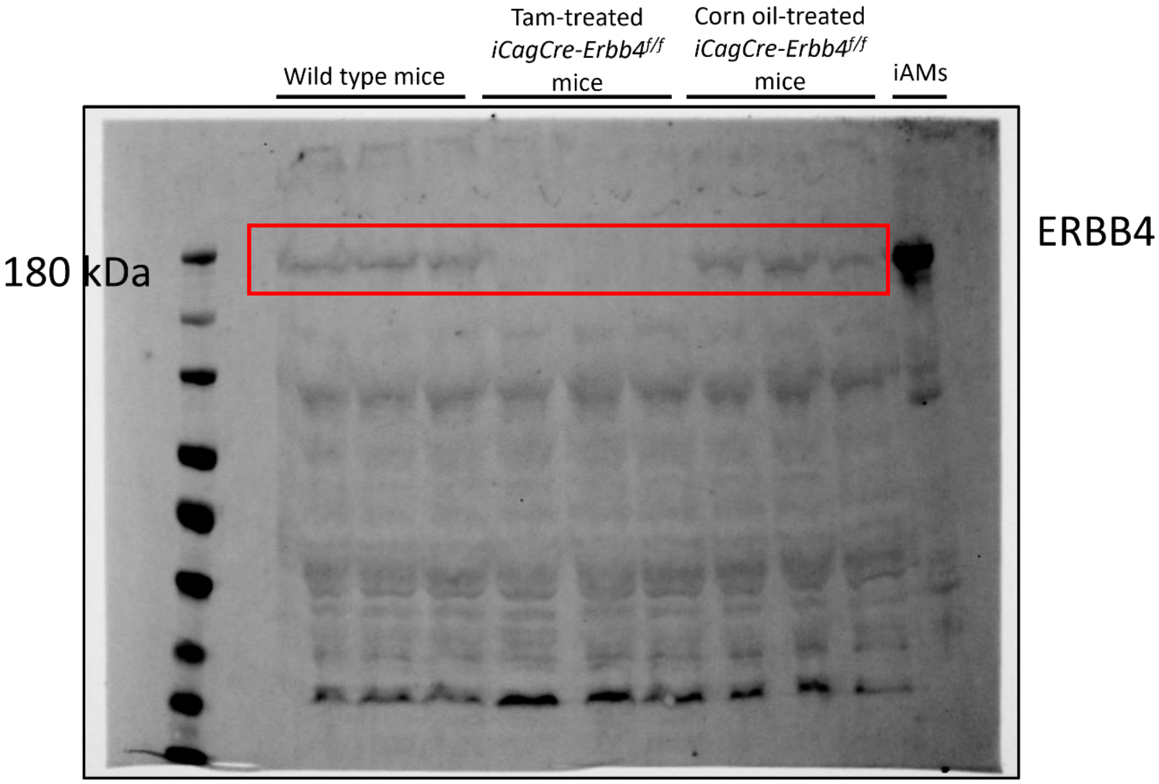

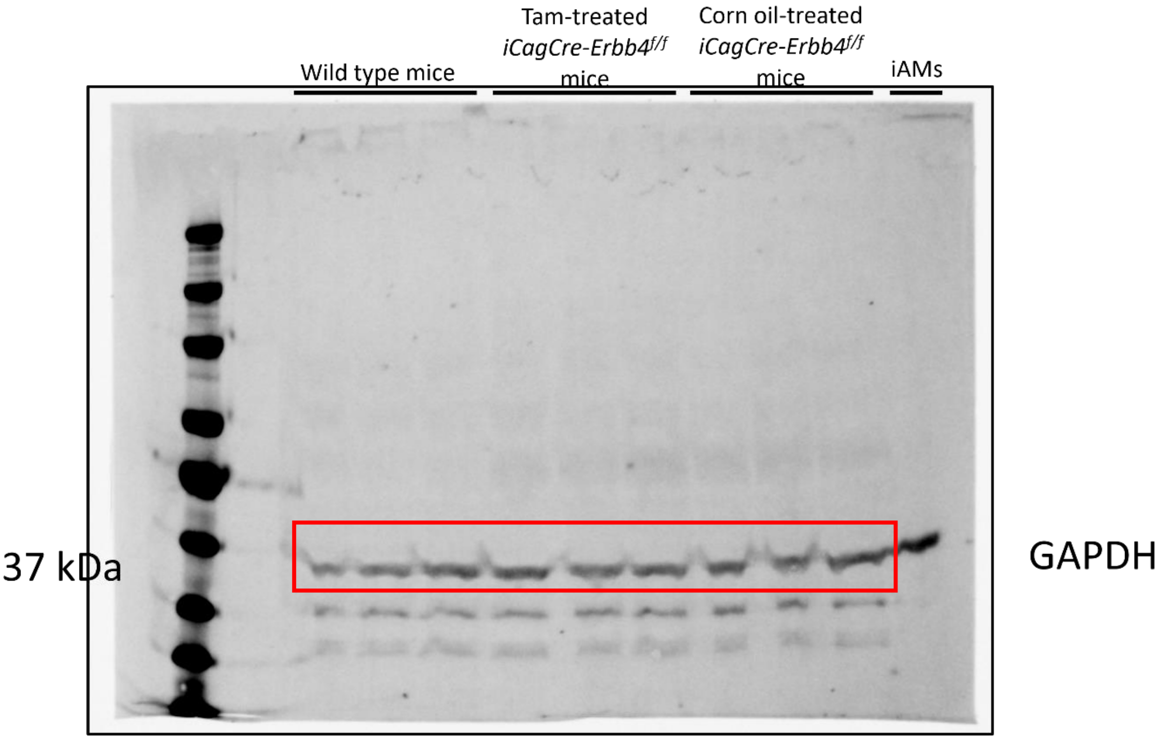

Supplementary Figure 9. Uncropped gels corresponding to western blots presented in **Supplementary Fig. 6b**. Areas shown in **Suppl. Fig 6b** are highlighted with red boxes.
