# Supplementary material for: Small-molecule-induced ERBB4 activation to treat heart failure
Source: Nat Commun. 2025 Jan 10;16:576. doi: 10.1038/s41467-024-54908-5 (PMC11724075; doi:10.1038/s41467-024-54908-5)
Supplement: Supplementary file 3 — Description of Additional Supplementary Files [file 41467_2024_54908_MOESM3_ESM.pdf]

Description of Additional Supplementary Files

File Name: Supplementary Data 1
Description: All raw data files of the LC-MS/MS.
