# Supplementary material for: Small-molecule-induced ERBB4 activation to treat heart failure
Source: Nat Commun. 2025 Jan 10;16:576. doi: 10.1038/s41467-024-54908-5 (PMC11724075; doi:10.1038/s41467-024-54908-5)
Supplement: Supplementary file 4 — Supplementary Data 1 [file 41467_2024_54908_MOESM4_ESM.pdf]

Dataset: Untitled
Last Altered: Friday, August 06, 2021 16:06:38 Romance Daylight Time
Printed: Friday, August 06, 2021 16:07:17 Romance Daylight Time

Method: C:\MassLynx_Projects\Medchem SU.PRO\MethDB\A.mdb 11 Jan 2021 11:59:51
Calibration: 06 Aug 2021 16:06:38

Compound name: A
Correlation coefficient: r = 0.981517, r^2 = 0.963376
Calibration curve: 17.9926 * x + -191.679
Response type: External Std, Area
Curve type: Linear, Origin: Exclude, Weighting: 1/x, Axis trans: None

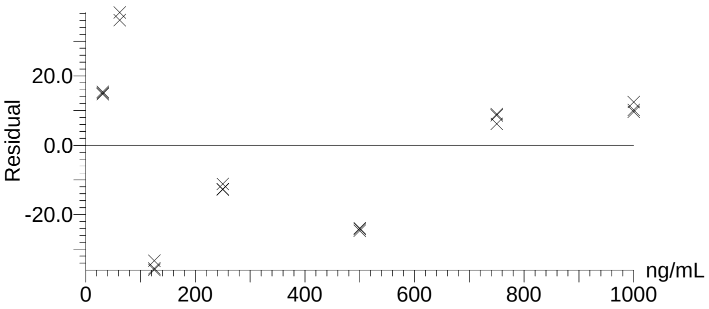

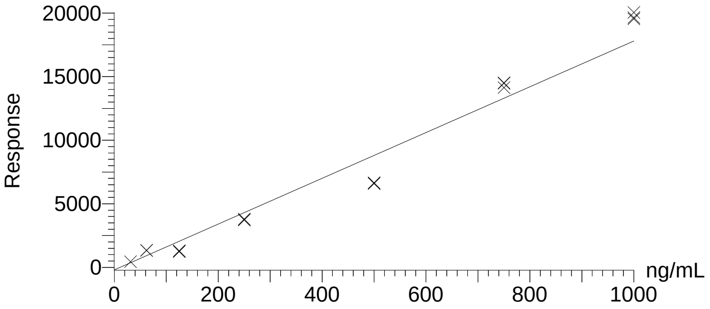

Dataset: Untitled
Last Altered: Friday, August 06, 2021 16:06:38 Romance Daylight Time
Printed: Friday, August 06, 2021 16:07:17 Romance Daylight Time

Method: C:\MassLynx_Projects\Medchem SU.PRO\MethDB\A.mdb 11 Jan 2021 11:59:51
Calibration: 06 Aug 2021 16:06:38

Sample Name: A HP 0805 31-1-1

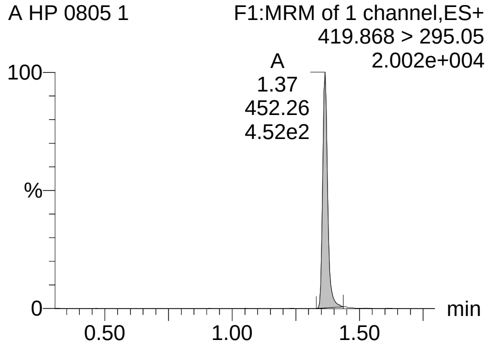

Sample Name: A HP 0805 31-1-2

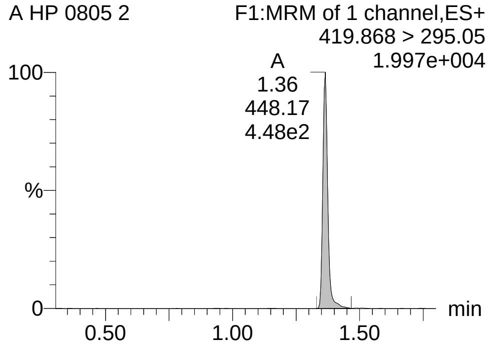

Sample Name: A HP 0805 31-1-3

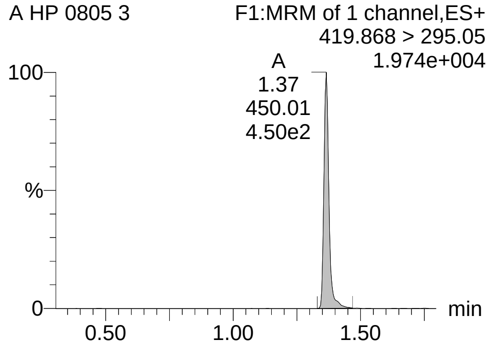

Sample Name: A HP 0805 62-1-1

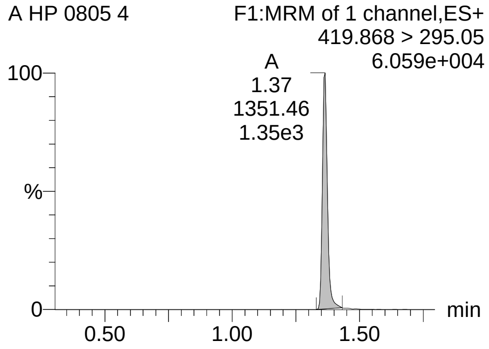

Sample Name: A HP 0805 62-1-2

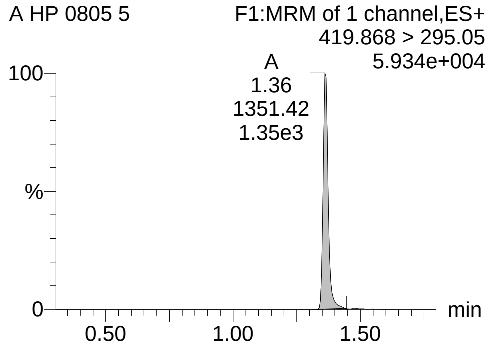

Sample Name: A HP 0805 62-1-3

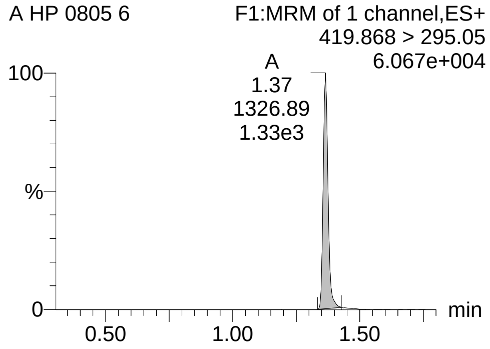

Sample Name: A HP 0805 125-1-1

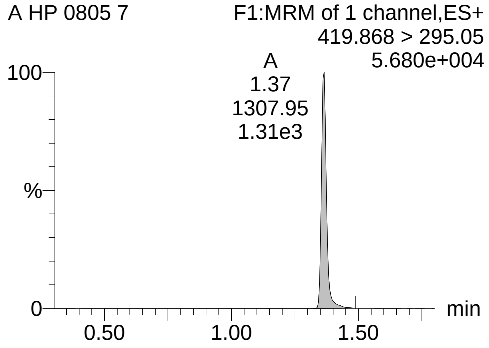

Sample Name: A HP 0805 125-1-2

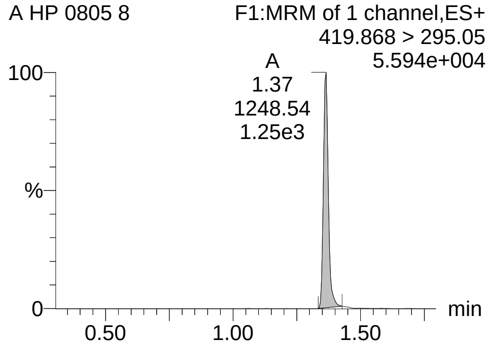

Sample Name: A HP 0805 125-1-3

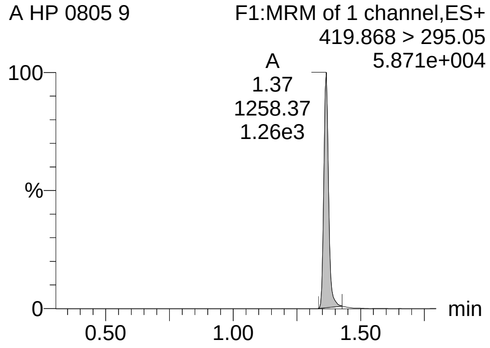

Sample Name: A HP 0805 250-1-1

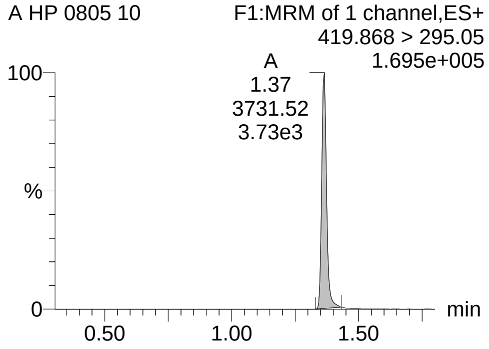

Sample Name: A HP 0805 250-1-2

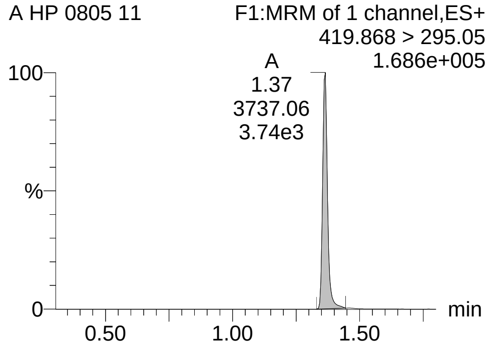

Sample Name: A HP 0805 250-1-3

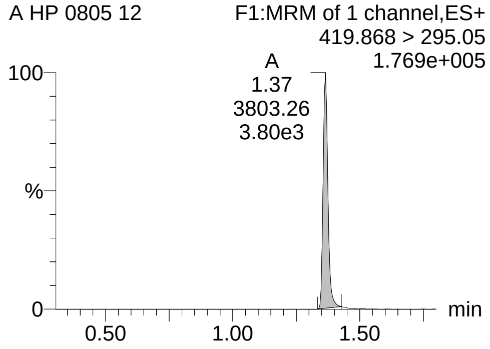

Sample Name: A HP 0805 500-1-1
A HP 0805 13 F1:MRM of 1 channel,ES+
419.868 > 295.05

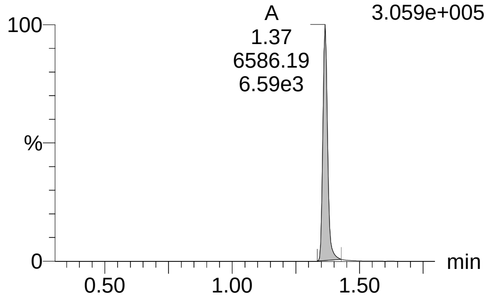

Sample Name: A HP 0805 500-1-2
A HP 0805 14 F1:MRM of 1 channel,ES+
419.868 > 295.05

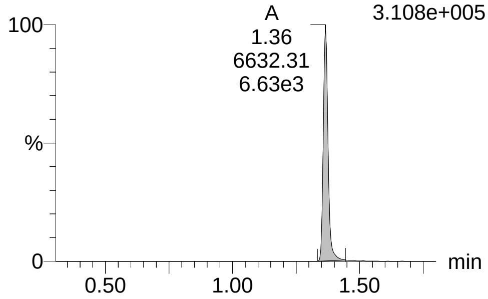

Sample Name: A HP 0805 500-1-3
A HP 0805 15 F1:MRM of 1 channel,ES+
419.868 > 295.05

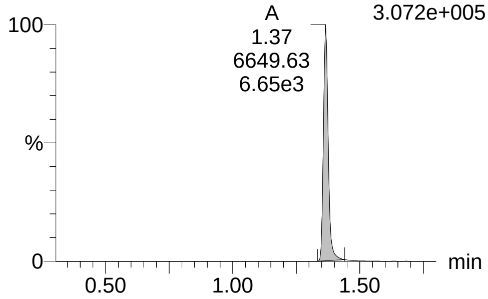

Sample Name: A HP 0805 750-1-1
A HP 0805 16 F1:MRM of 1 channel,ES+
419.868 > 295.05

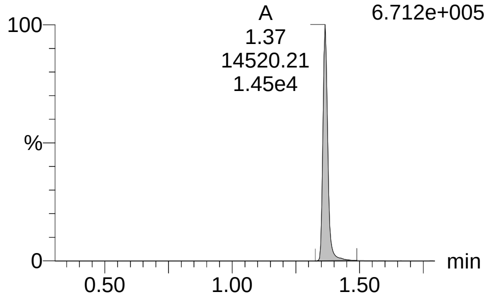

Sample Name: A HP 0805 750-1-2
A HP 0805 17 F1:MRM of 1 channel,ES+
419.868 > 295.05

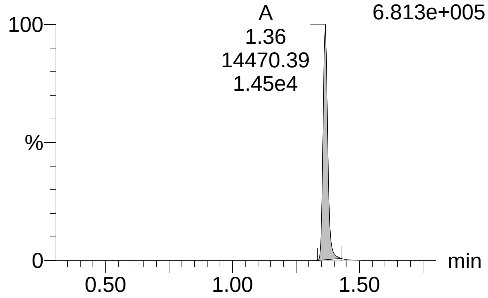

Sample Name: A HP 0805 750-1-3
A HP 0805 18 F1:MRM of 1 channel,ES+
419.868 > 295.05

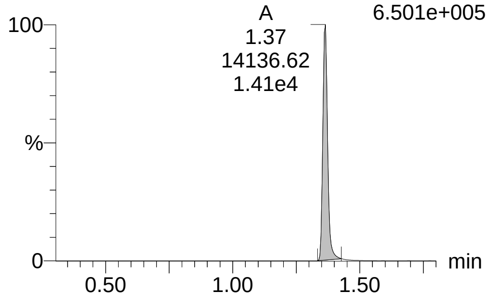

Sample Name: A HP 0805 1000-1-1
A HP 0805 19 F1:MRM of 1 channel,ES+
419.868 > 295.05

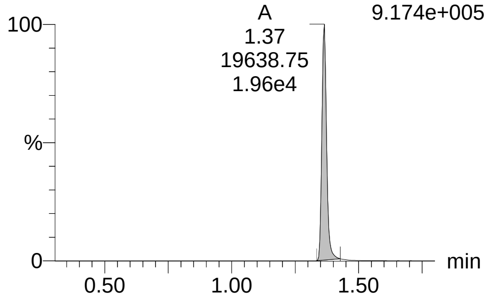

Sample Name: A HP 0805 1000-1-2
A HP 0805 20 F1:MRM of 1 channel,ES+
419.868 > 295.05

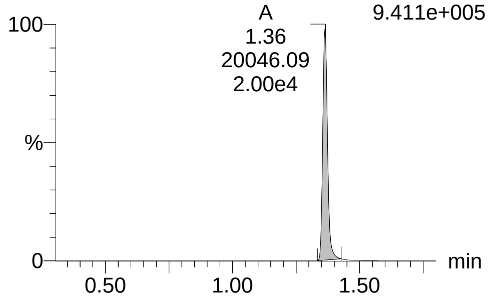

Sample Name: A HP 0805 1000-1-3
A HP 0805 21 F1:MRM of 1 channel,ES+
419.868 > 295.05

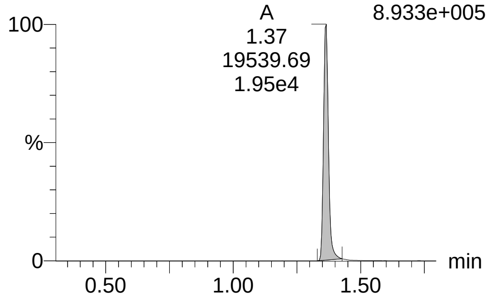

Sample Name: A HP 0805 Blank 1
A HP 0805 22 F1:MRM of 1 channel,ES+
419.868 > 295.05

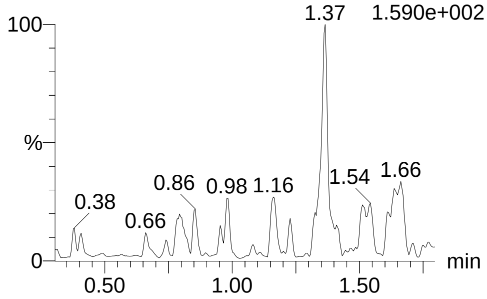

Sample Name: A HP 0805 0-1-1
A HP 0805 23 F1:MRM of 1 channel,ES+
419.868 > 295.05

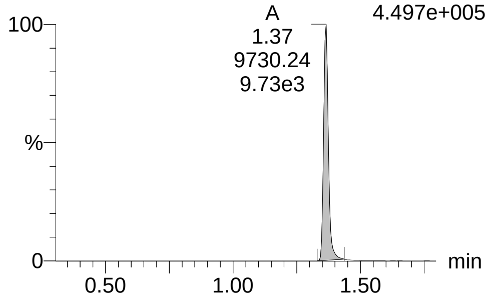

Sample Name: A HP 0805 0-1-2
A HP 0805 24 F1:MRM of 1 channel,ES+
419.868 > 295.05

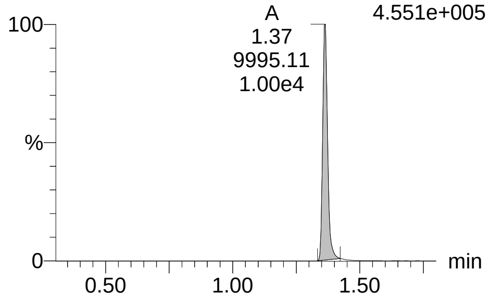

Sample Name: A HP 0805 0-1-3
A HP 0805 25 F1:MRM of 1 channel,ES+
419.868 > 295.05

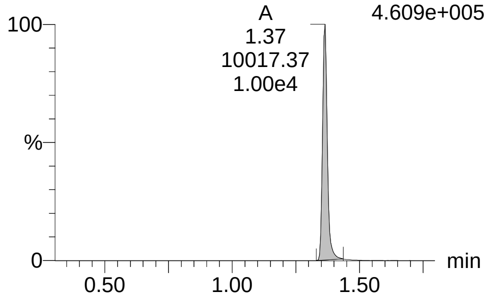

Sample Name: A HP 0805 0-2-1
A HP 0805 26 F1:MRM of 1 channel,ES+
419.868 > 295.05

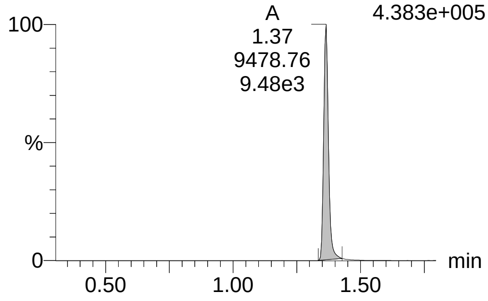

Sample Name: A HP 0805 0-2-2
A HP 0805 27 F1:MRM of 1 channel,ES+
419.868 > 295.05

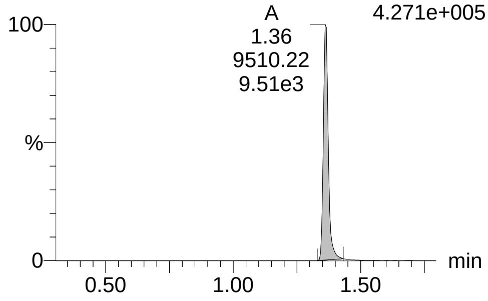

Sample Name: A HP 0805 0-2-3
A HP 0805 28 F1:MRM of 1 channel,ES+
419.868 > 295.05

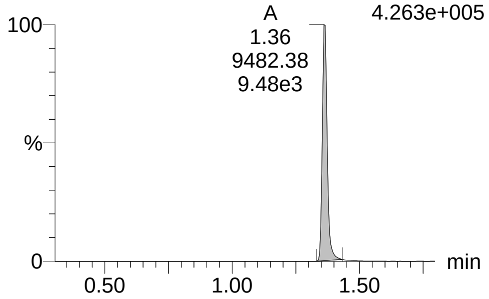

Sample Name: A HP 0805 Blank 2
A HP 0805 29 F1:MRM of 1 channel,ES+
419.868 > 295.05

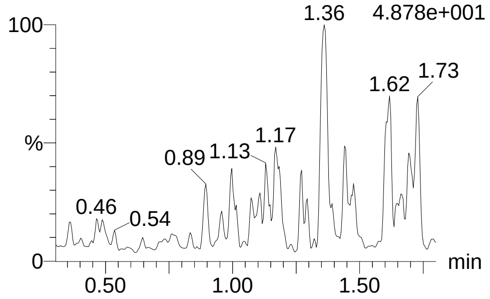

Sample Name: A HP 0805 30-1-1
A HP 0805 30 F1:MRM of 1 channel,ES+
419.868 > 295.05

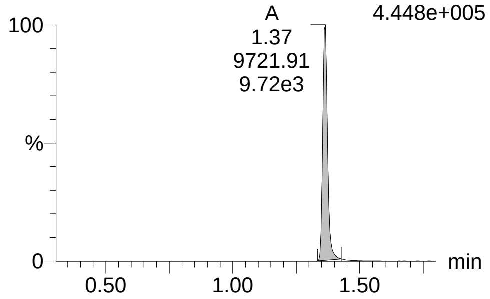

Sample Name: A HP 0805 30-1-2
A HP 0805 31 F1:MRM of 1 channel,ES+
419.868 > 295.05

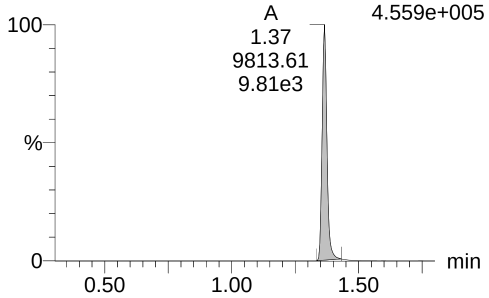

Sample Name: A HP 0805 30-1-3
A HP 0805 32 F1:MRM of 1 channel,ES+
419.868 > 295.05

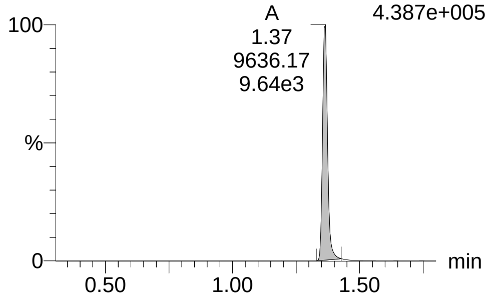

Sample Name: A HP 0805 30-2-1
A HP 0805 33 F1:MRM of 1 channel,ES+
419.868 > 295.05

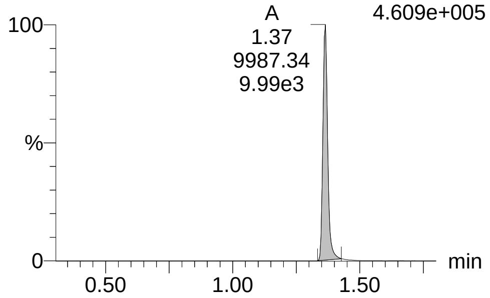

Sample Name: A HP 0805 30-2-2
A HP 0805 34 F1:MRM of 1 channel,ES+
419.868 > 295.05

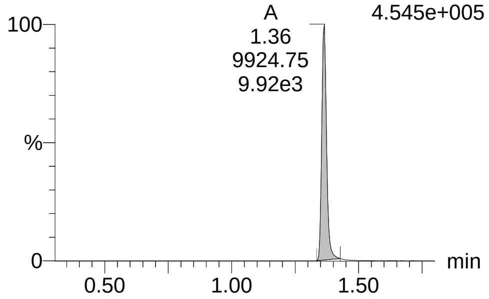

Sample Name: A HP 0805 30-2-3
A HP 0805 35 F1:MRM of 1 channel,ES+
419.868 > 295.05

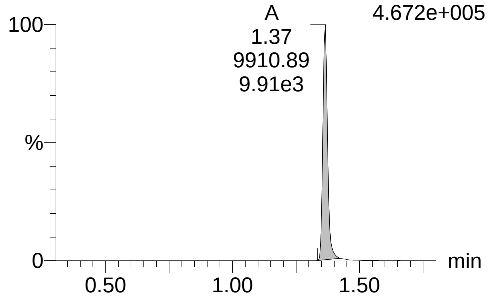

Sample Name: A HP 0805 Blank 3
A HP 0805 36 F1:MRM of 1 channel,ES+
419.868 > 295.05

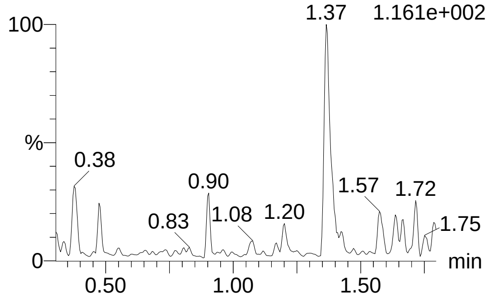

Sample Name: A HP 0805 1-1-1
A HP 0805 37 F1:MRM of 1 channel,ES+
419.868 > 295.05

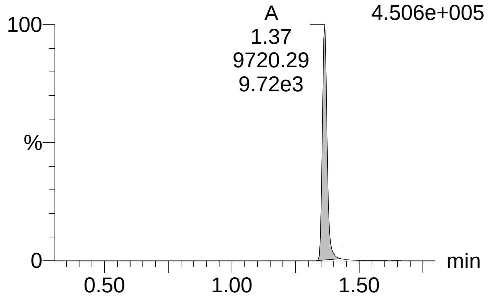

Sample Name: A HP 0805 1-1-2
A HP 0805 38 F1:MRM of 1 channel,ES+
419.868 > 295.05

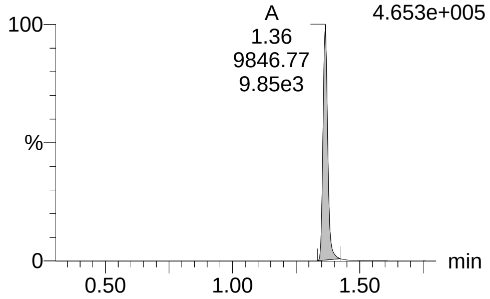

Sample Name: A HP 0805 1-1-3
A HP 0805 39 F1:MRM of 1 channel,ES+
419.868 > 295.05

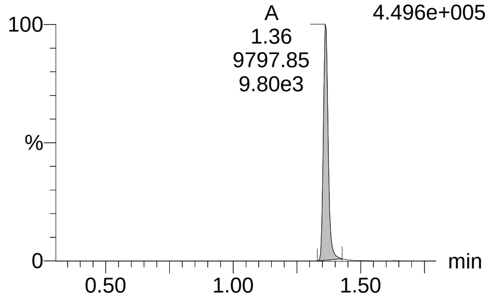

Sample Name: A HP 0805 1-2-1
A HP 0805 40 F1:MRM of 1 channel,ES+
419.868 > 295.05

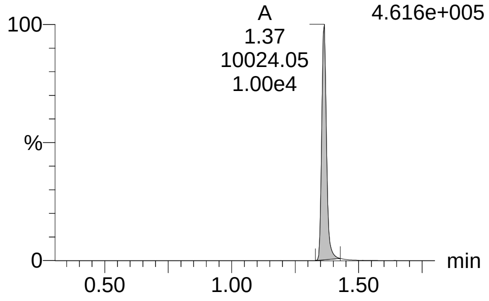

Sample Name: A HP 0805 1-2-2
A HP 0805 41 F1:MRM of 1 channel,ES+
419.868 > 295.05

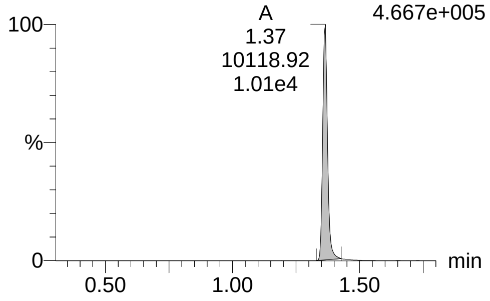

Sample Name: A HP 0805 1-2-3
A HP 0805 42 F1:MRM of 1 channel,ES+
419.868 > 295.05

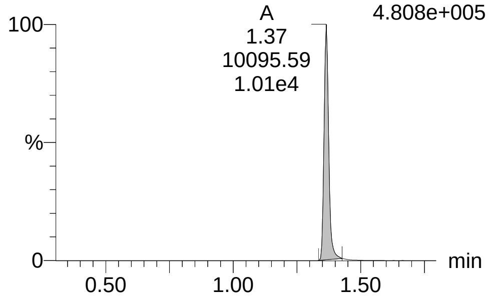

Sample Name: A HP 0805 Blank 4
A HP 0805 43 F1:MRM of 1 channel,ES+
419.868 > 295.05
1.152e+002

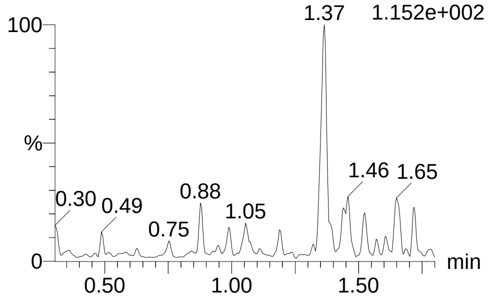

Sample Name: A HP 0805 2-1-1
A HP 0805 44 F1:MRM of 1 channel,ES+
419.868 > 295.05
4.411e+005

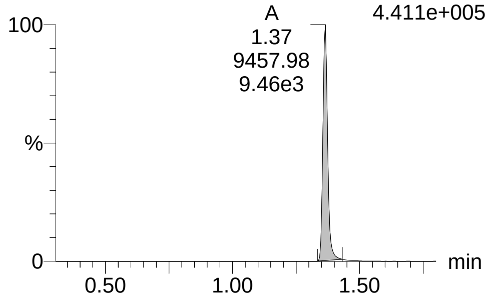

Sample Name: A HP 0805 2-1-2
A HP 0805 45 F1:MRM of 1 channel,ES+
419.868 > 295.05
4.414e+005

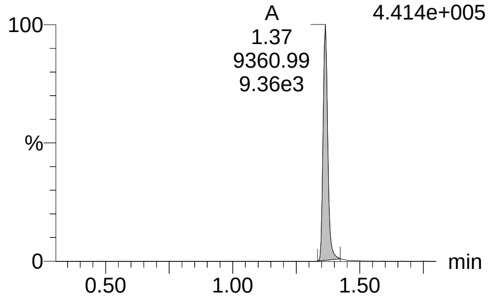

Sample Name: A HP 0805 2-1-3
A HP 0805 46 F1:MRM of 1 channel,ES+
419.868 > 295.05
4.301e+005

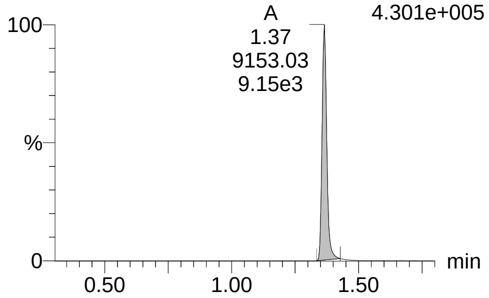

Sample Name: A HP 0805 2-2-1
A HP 0805 47 F1:MRM of 1 channel,ES+
419.868 > 295.05
4.648e+005

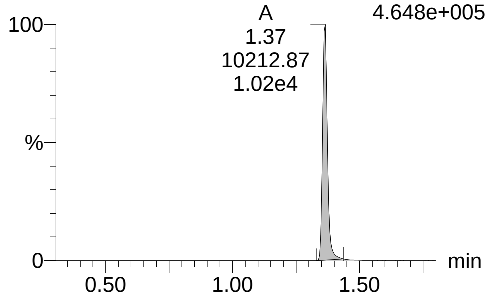

Sample Name: A HP 0805 2-2-2
A HP 0805 48 F1:MRM of 1 channel,ES+
419.868 > 295.05
4.565e+005

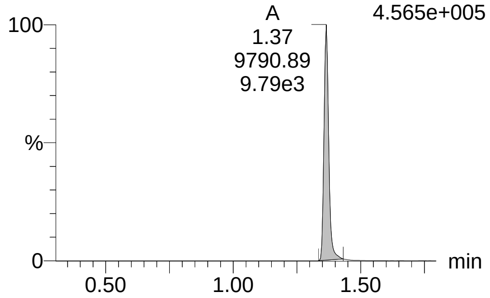

Sample Name: A HP 0805 2-2-3
A HP 0805 49 F1:MRM of 1 channel,ES+
419.868 > 295.05
4.671e+005

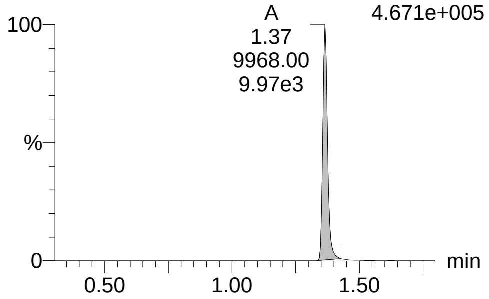

Sample Name: A HP 0805 Blank 5
A HP 0805 50 F1:MRM of 1 channel,ES+
419.868 > 295.05
5.517e+001

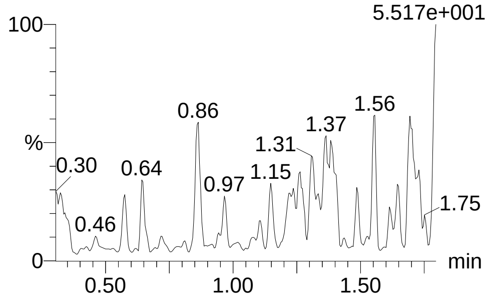

Sample Name: A HP 0805 3-1-1
A HP 0805 51 F1:MRM of 1 channel,ES+
419.868 > 295.05
4.660e+005

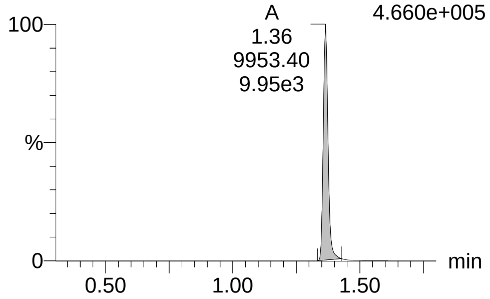

Sample Name: A HP 0805 3-1-2
A HP 0805 52 F1:MRM of 1 channel,ES+
419.868 > 295.05
4.697e+005

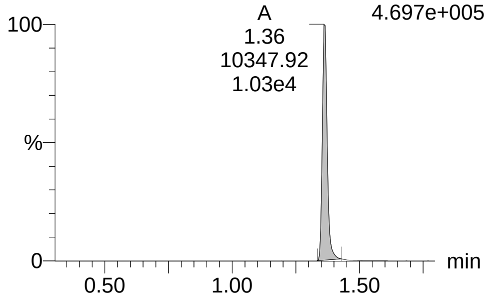

Sample Name: A HP 0805 3-1-3
A HP 0805 53 F1:MRM of 1 channel,ES+
419.868 > 295.05
1.161e+002

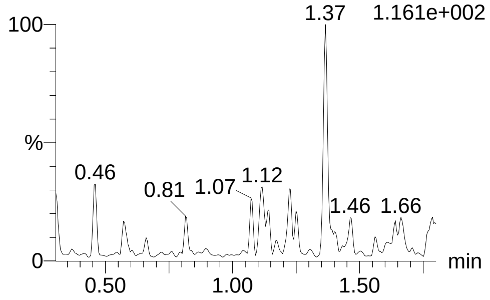

Sample Name: A HP 0805 3-2-1
A HP 0805 54 F1:MRM of 1 channel,ES+
419.868 > 295.05
4.795e+005

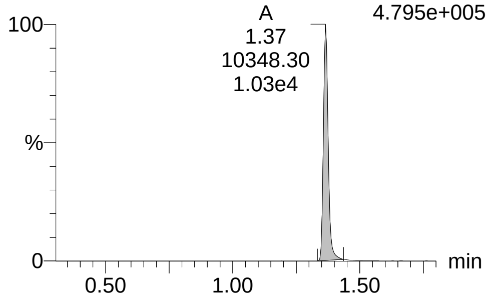

Sample Name: A HP 0805 3-2-2
A HP 0805 55 F1:MRM of 1 channel,ES+
419.868 > 295.05
4.772e+005

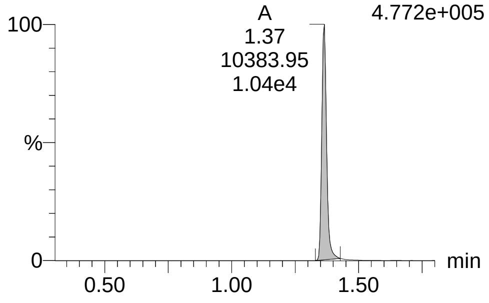

Sample Name: A HP 0805 3-2-3
A HP 0805 56 F1:MRM of 1 channel,ES+
419.868 > 295.05
4.891e+005

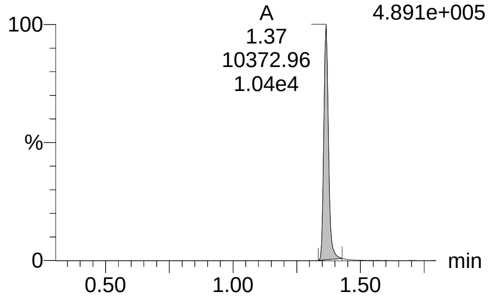

Sample Name: A HP 0805 Blank 6
A HP 0805 57 F1:MRM of 1 channel,ES+
419.868 > 295.05
6.000e+001

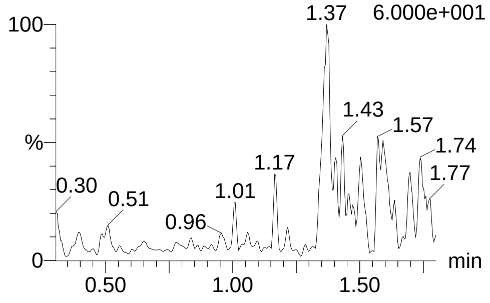

Sample Name: A HP 0805 6-1-1

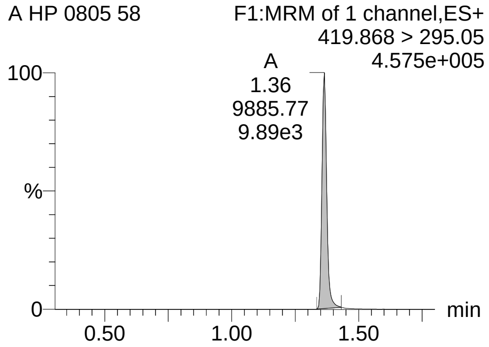

Sample Name: A HP 0805 6-1-2

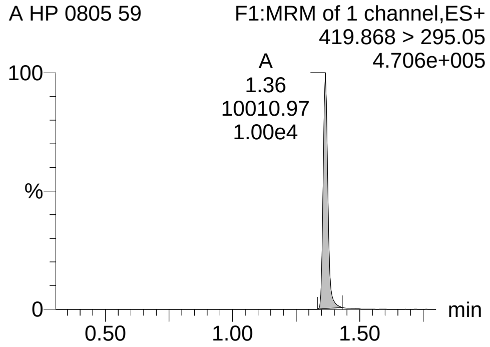

Sample Name: A HP 0805 6-1-3

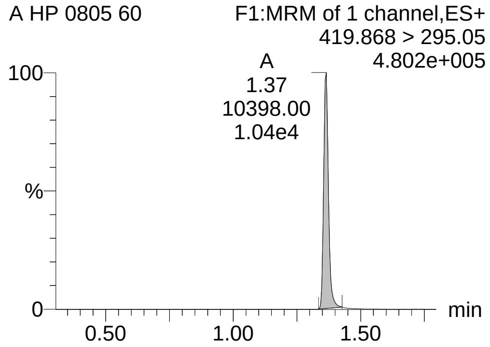

Sample Name: A HP 0805 6-2-1

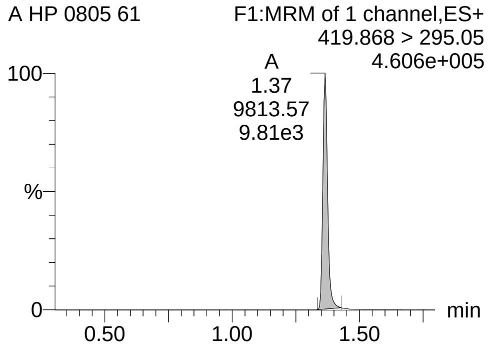

Sample Name: A HP 0805 6-2-2

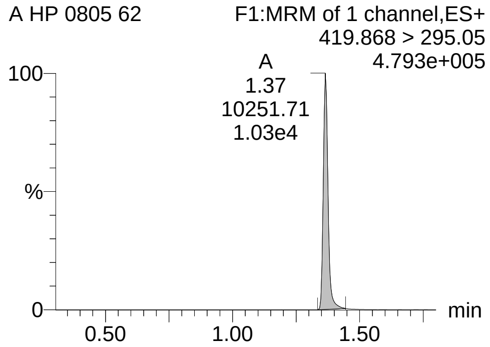

Sample Name: A HP 0805 6-2-3

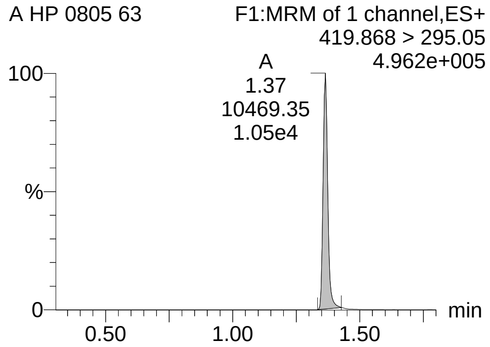

Dataset: Untitled
Last Altered: Friday, August 06, 2021 16:06:38 Romance Daylight Time
Printed: Friday, August 06, 2021 16:07:17 Romance Daylight Time

	#	Name	Type	Std. Conc	RT	Area	IS Area	Response	Detecti...	ng/mL	%Dev
1	1	A HP 0805 31-1-1	Stan...	31.000	1.37	452.262		452.262	bb	35.8	15.4
2	2	A HP 0805 31-1-2	Stan...	31.000	1.36	448.172		448.172	bb	35.6	14.7
3	3	A HP 0805 31-1-3	Stan...	31.000	1.37	450.010		450.010	bb	35.7	15.0
4	4	A HP 0805 62-1-1	Stan...	62.000	1.37	1351.462		1351.462	bb	85.8	38.3
5	5	A HP 0805 62-1-2	Stan...	62.000	1.36	1351.417		1351.417	bb	85.8	38.3
6	6	A HP 0805 62-1-3	Stan...	62.000	1.37	1326.890		1326.890	bb	84.4	36.1
7	7	A HP 0805 125-1-1	Stan...	125.000	1.37	1307.955		1307.955	bb	83.3	-33.3
8	8	A HP 0805 125-1-2	Stan...	125.000	1.37	1248.538		1248.538	bb	80.0	-36.0
9	9	A HP 0805 125-1-3	Stan...	125.000	1.37	1258.365		1258.365	bb	80.6	-35.5
10	10	A HP 0805 250-1-1	Stan...	250.000	1.37	3731.521		3731.521	bb	218.0	-12.8
11	11	A HP 0805 250-1-2	Stan...	250.000	1.37	3737.057		3737.057	bb	218.4	-12.7
12	12	A HP 0805 250-1-3	Stan...	250.000	1.37	3803.258		3803.258	bb	222.0	-11.2
13	13	A HP 0805 500-1-1	Stan...	500.000	1.37	6586.194		6586.194	bb	376.7	-24.7
14	14	A HP 0805 500-1-2	Stan...	500.000	1.36	6632.315		6632.315	bb	379.3	-24.1
15	15	A HP 0805 500-1-3	Stan...	500.000	1.37	6649.627		6649.627	bb	380.2	-24.0
16	16	A HP 0805 750-1-1	Stan...	750.000	1.37	14520.214		14520.214	bb	817.7	9.0
17	17	A HP 0805 750-1-2	Stan...	750.000	1.36	14470.389		14470.389	bb	814.9	8.7
18	18	A HP 0805 750-1-3	Stan...	750.000	1.37	14136.617		14136.617	bb	796.3	6.2
19	19	A HP 0805 1000-1-1	Stan...	1000.000	1.37	19638.746		19638.746	bb	1102.1	10.2
20	20	A HP 0805 1000-1-2	Stan...	1000.000	1.36	20046.088		20046.088	bb	1124.8	12.5
21	21	A HP 0805 1000-1-3	Stan...	1000.000	1.37	19539.691		19539.691	bb	1096.6	9.7
22	22	A HP 0805 Blank 1	Blank								
23	23	A HP 0805 0-1-1	Anal...		1.37	9730.243		9730.243	bb	551.4	
24	24	A HP 0805 0-1-2	Anal...		1.37	9995.113		9995.113	bb	566.2	
25	25	A HP 0805 0-1-3	Anal...		1.37	10017.368		10017.368	bb	567.4	
26	26	A HP 0805 0-2-1	Anal...		1.37	9478.764		9478.764	bb	537.5	
27	27	A HP 0805 0-2-2	Anal...		1.36	9510.215		9510.215	bb	539.2	
28	28	A HP 0805 0-2-3	Anal...		1.36	9482.383		9482.383	bb	537.7	
29	29	A HP 0805 Blank 2	Blank								
30	30	A HP 0805 30-1-1	Anal...		1.37	9721.905		9721.905	bb	551.0	
31	31	A HP 0805 30-1-2	Anal...		1.37	9813.613		9813.613	bb	556.1	
32	32	A HP 0805 30-1-3	Anal...		1.37	9636.169		9636.169	bb	546.2	
33	33	A HP 0805 30-2-1	Anal...		1.37	9987.337		9987.337	bb	565.7	
34	34	A HP 0805 30-2-2	Anal...		1.36	9924.747		9924.747	bb	562.3	
35	35	A HP 0805 30-2-3	Anal...		1.37	9910.893		9910.893	bb	561.5	
36	36	A HP 0805 Blank 3	Blank								
37	37	A HP 0805 1-1-1	Anal...		1.37	9720.288		9720.288	bb	550.9	
38	38	A HP 0805 1-1-2	Anal...		1.36	9846.772		9846.772	bb	557.9	
39	39	A HP 0805 1-1-3	Anal...		1.36	9797.854		9797.854	bb	555.2	
40	40	A HP 0805 1-2-1	Anal...		1.37	10024.047		10024.047	bb	567.8	
41	41	A HP 0805 1-2-2	Anal...		1.37	10118.918		10118.918	bb	573.0	
42	42	A HP 0805 1-2-3	Anal...		1.37	10095.590		10095.590	bb	571.7	
43	43	A HP 0805 Blank 4	Blank								
44	44	A HP 0805 2-1-1	Anal...		1.37	9457.978		9457.978	bb	536.3	
45	45	A HP 0805 2-1-2	Anal...		1.37	9360.990		9360.990	bb	530.9	
46	46	A HP 0805 2-1-3	Anal...		1.37	9153.032		9153.032	bb	519.4	
47	47	A HP 0805 2-2-1	Anal...		1.37	10212.867		10212.867	bb	578.3	
48	48	A HP 0805 2-2-2	Anal...		1.37	9790.886		9790.886	bb	554.8	
49	49	A HP 0805 2-2-3	Anal...		1.37	9968.002		9968.002	bb	564.7	
50	50	A HP 0805 Blank 5	Blank								
51	51	A HP 0805 3-1-1	Anal...		1.36	9953.403		9953.403	bb	563.8	
52	52	A HP 0805 3-1-2	Anal...		1.36	10347.916		10347.916	bb	585.8	
53	53	A HP 0805 3-1-3	Anal...								
54	54	A HP 0805 3-2-1	Anal...		1.37	10348.301		10348.301	bb	585.8	
55	55	A HP 0805 3-2-2	Anal...		1.37	10383.954		10383.954	bb	587.8	
56	56	A HP 0805 3-2-3	Anal...		1.37	10372.957		10372.957	bb	587.2	
57	57	A HP 0805 Blank 6	Blank								
58	58	A HP 0805 6-1-1	Anal...		1.36	9885.768		9885.768	bb	560.1	

	#	Name	Type	Std. Conc	RT	Area	IS Area	Response	Detecti...	ng/mL	%Dev
59	59	A HP 0805 6-1-2	Anal...		1.36	10010.969		10010.969	bb	567.0	
60	60	A HP 0805 6-1-3	Anal...		1.37	10398.004		10398.004	bb	588.6	
61	61	A HP 0805 6-2-1	Anal...		1.37	9813.566		9813.566	bb	556.1	
62	62	A HP 0805 6-2-2	Anal...		1.37	10251.705		10251.705	bb	580.4	
63	63	A HP 0805 6-2-3	Anal...		1.37	10469.347		10469.347	bb	592.5	

Method: C:\MassLynx_Projects\Medchem SU.PRO\MethDB\A.mdb 11 Jan 2021 11:59:51
Calibration: 06 Aug 2021 16:06:38

```
Header
Acquired File Name:   A HP 0805 31-1-1
Acquired Date:        06-Aug-2021
Acquired Time:        03:42:00
Job Code:             PSHP - 20210805 - N - 216 - 075 - A
Task Code:
User Name:
Laboratory Name:
Instrument:           ACQ-TQD#QBA320
Conditions:
Submitter:
SampleID:             A HP 0805 1
Bottle Number:        2:79
Description:
Instrument Calibration:
Calibration File: C:\MassLynx\IntelliStart\Results\Unit Mass Resolution\Calib 20161209.cal
Parameters
MS1 Static:
Mass:             20 Da to 1974 Da.
Resolution:       15.0/15.0
Ion Energy:       0.5
Reference File:   Naics2
Acquisition File: STATMS1
MS1 Scanning:
Mass:             15 Da to 2048 Da.
Resolution:       15.0/15.0
Ion Energy:       0.5
Reference File:   Naics2
Acquisition File: SCNMS1
MS1 Scan Speed Compensation:
Scan:             339 to 2000 amu/sec.
Resolution:       15.0/15.0
Ion Energy:       0.5
Reference File:   Naics2
Acquisition File: FASTMS1
MS2 Static:
Mass:             20 Da to 1974 Da.
Resolution:       15.0/15.0
Ion Energy:       0.5
Reference File:   Naics2
Acquisition File: STATMS2
MS2 Scanning:
Mass:             15 Da to 2048 Da.
Resolution:       15.0/15.0
Ion Energy:       0.5
Reference File:   Naics2
Acquisition File: SCNMS2
MS2 Scan Speed Compensation:
Scan:             339 to 10165 amu/sec.
Resolution:       15.0/15.0
Ion Energy:       0.5
Reference File:   Naics2
Acquisition File: FASTMS2
Calibration Time: 10:33
Calibration Date: 12/09/16
Coefficients
MS1 Static:       -0.000000000000*x^4 + 0.000000000843*x^3 + -0.000001360559*x^2 + 1.000940122634*x
 +-0.253266439024
MS2 Static:       -0.000000000000*x^4 + 0.000000000573*x^3 + -0.000001239648*x^2 + 1.000902600483*x
 +-0.192715155436
Function 1:       None
Function 2:       None
Parameters for C:\Documents and Settings\Administrator\Desktop\old desktop\log D\EA.EXP
Data Processing:
SIR / MRM Chromatogram Spike Removal      ON
```

```
SIR / MRM Smoothing           OFF
Smoothing window size (scans) 3
Number of smooths       2
Prescan Statistics:
Initial Average Intensity     21.9169
Initial Average Std Dev       1.7908
Bunch Zero Level        0.0027
Bunch Std Dev                 0.0088
Bunch Threshold               0.0702
Spike Removal Std Dev         1.7757
Method Events:
Initial Stop Flow:      No Change
Initial Switch 2: No Change
Initial Switch 3: No Change
Initial Switch 4: No Change
Initial Infusion: No Change
Initial Flow State:     LC
Initial Flow Rate:      5
Initial Reservoir:      No Action
API Probe Delay Temp:   20
Initial Refill:         No Action
Timed Events Enabled
Event Time  Name        Action
Instrument Parameters - Function 1:
Parameter File - C:\Documents and Settings\Administrator\Desktop\old desktop\log D\log D tune file.
IPR
Polarity    ES+
Calibration Static 2
Capillary (kV)    3.50  3.48
Cone (V)    38.00 33.46
Extractor (V)     3.00  2.69
RF (V)      0.10
Source Temperature (°C) 140   139
Desolvation Temperature (°C)  450   444
Cone Gas Flow (L/Hr)    90    90
Desolvation Gas Flow (L/Hr)   900   900
Collision Gas Flow (mL/Min)   0.20  0.20
LM 1 Resolution   15.50
HM 1 Resolution   14.84
Ion Energy 1      0.30
MS Mode Entrance  50.00
MS Mode Collision Energy      3.00
MS Mode Exit      50.00
MSMS Mode Entrance      -2.00
MSMS Mode Collision Energy    2.00
MSMS Mode Exit    2.00
LM 2 Resolution   15.00
HM 2 Resolution   15.50
Ion Energy 2      1.22
Gain  1.00
Multiplier  -493.56
Active Reservoir  B
Engineers Settings:
MS1 Low Mass Position         518
MS1 High Mass Position        284
MS1 Low Mass Resolution       513
MS1 High Mass Resolution      1732
MS1 Resolution Linearity      834
MS1 High Mass DC Balance      0
MS1 DC Polarity               Positive
MS2 Low Mass Position         519
MS2 High Mass Position        238
MS2 Low Mass Resolution       518
MS2 High Mass Resolution      644
MS2 Resolution Linearity      756
MS2 High Mass DC Balance      -0
MS2 DC Polarity               Positive
HM RF Lens Correction +       0
```

```
HM RF Lens Correction -       0
Inter-scan delays:
Automatic Mode
MS 1 Delay Table:
         R     delay
<=   0.500     0.005
<=   1.200     0.010
<=   2.400     0.015
<=   6.000     0.020
<=  15.000     0.025
<=  25.000     0.028
>   25.000     0.030
MS 2 Delay Table:
         R     delay
<=   2.000     0.005
<=   4.000     0.008
<=   7.000     0.010
<=  10.000     0.012
<=  20.000     0.014
>   20.000     0.016
ACE Experimental Record
Inlet Method File: c:\masslynx_projects\medchem su.pro\acqudb\adme sophie
-------------------   Prerun method parameters   ---------------
Waters ACQUITY QSM
Waters Acquity TUV
 Run Time: 0.20 min
 Wavelength Mode: Single Wavelength
 Lamp On: On
Channel A...
 Comment:
 Wavelength: 254 nm
 Sampling Rate: 20 points/sec
 Data Mode: Absorbance
 Time Constant: 0.1000 sec
 Auto Zero On Wavelength Change: Maintain Baseline
 Auto Zero On Inject Start: Yes
Analog 1...
 Sensitivity: 2.000 AUFS
 Chart Polarity: Positive (+)
 Voltage Offset: 0 mV
 Enable Chart Mark: Yes
 Run Events: Yes
 Pulse Width: 1.0 sec
 Rect Wave Period: 0.2 sec
----------------------------  oOo  ---------------------------
---------------------   Run method parameters   ----------------
Waters ACQUITY QSM
 Solvent A Name: Water
 Solvent B Name: Acetonitrile
 Solvent C Name: water 1 % FA
 Solvent D Name:
 Low Pressure Limit: 0 psi
 High Pressure Limit: 15000 psi
 Seal Wash Period: 5.00 min
 [Gradient Table]
  Time(min) Flow Rate(mL/min) %A %B %C %D Curve
 1. Initial 0.700 90.0 5.0 5.0 0.0 Initial
 2. 0.15 0.700 45.0 50.0 5.0 0.0 6
 3. 1.50 0.700 0.0 95.0 5.0 0.0 6
 4. 1.80 0.700 90.0 5.0 5.0 0.0 1
 Comment: ACQUITY UPLC BEH C18 2.1 x 50 mm
 Flow Ramp Rate: 0.45 min
 D Solvent Selection (if supported): No Change
 System Pressure Data Channel: No
 Flow Rate Data Channel: No
 %A Data Channel: No
 %B Data Channel: No
 %C Data Channel: No
```

```
 %D Data Channel: No
 Primary Data Channel: No
 Accumulator Data Channel: No
 Degasser Data Channel: No
 Gradient Start: At Injection
 Gradient Start Volume: 0 uL
 Gradient Start Time: 0.00 min
 Participate in pre-analysis: No
Waters Acquity TUV
 Run Time: 1.80 min
 Wavelength Mode: Single Wavelength
 Lamp On: On
Channel A...
 Comment:
 Wavelength: 214 nm
 Sampling Rate: 20 points/sec
 Data Mode: Absorbance
 Time Constant: 0.1000 sec
 Auto Zero On Wavelength Change: Maintain Baseline
 Auto Zero On Inject Start: Yes
Analog 1...
 Sensitivity: 2.000 AUFS
 Chart Polarity: Positive (+)
 Voltage Offset: 0 mV
 Enable Chart Mark: Yes
 Run Events: Yes
 Pulse Width: 1.0 sec
 Rect Wave Period: 0.2 sec
Waters ACQUITY FTN AutoSampler
 Run Time: 1.80 min
 Comment: ACQUITY UPLC BEH C18 2.1 x 50 mm
 Load Ahead: Disabled
 Loop Offline: Automatic min
 Wash Solvent Name: Acetonitrile
 Pre-Inject Wash Time: 0.0 sec
 Post-Inject Wash Time: 6.0 sec
 Purge Solvent Name: Water
 Dilution: Disabled
 Dilution Volume: 0 uL
 Delay Time: 0 min
 Dilution Needle Placement: 4.0 mm
 Target Column Temperature: 40.0 C
 Column Temperature Alarm Band: Disabled
 Target Sample Temperature: 15.0 C
 Sample Temperature Alarm Band: Disabled
 Syringe Draw Rate: Automatic
 Needle Placement: Automatic
 Pre-Aspirate Air Gap: Automatic
 Post-Aspirate Air Gap: Automatic
 Column Temperature Data Channel: No
 Room Temperature Data Channel: No
 Sample Temperature Data Channel: No
 Sample Organizer Temperature Data Channel: No
 Sample Pressure Data Channel: No
 Preheater Temperature Data Channel: No
 Seal Force Data Channel: No
 No Injection Mode Enabled: No
 Run Events: No
Sample Run Injection Parameter
Injection Volume (ul)   -   3.00
----------------------------  oOo  ----------------------------
End of experimental record.
-------------------- Waters ACQUITY QSM Postrun Report  ---------------
 Firmware Version: 1.50.237 (May 18 2011)
 Software Version: 1.50.1621
 Checksum: 0xae400516
 Serial Number: M09QSM056N
 Minimum System Pressure: 0.0 psi
```

```
 Maximum System Pressure: 0.0 psi
 Average System Pressure: 0.0 psi
----------------------------  oOo  ----------------------------
------------------- Waters ACQUITY FTN Postrun Report  ---------------
 Software Version: 1.50.1481
 Firmware Version: 1.50.317 (Jul 11 2011)
 Checksum: 0x3e83519d
 Serial Number: M09SDI055N
 Sample Syringe Size: 100.0
 Extension Loop Size: 0.0
 Needle Size: 15.0
 Column Type: ACQUITY UPLC® BEH C18 1.7µm
 Column Serial Number: 02343407715781
 Total Injections on Column: 6319
 Minimum Sample Temperature: 0.0
 Maximum Sample Temperature: 0.0
 Average Sample Temperature: 0.0
 Minimum Column Temperature: 40.0
 Maximum Column Temperature: 40.1
 Average Column Temperature: 0.0
----------------------------  oOo  ----------------------------
------------------- Generic Instrument Postrun Report  ---------------
 Software Version: 1.50.2530
 Firmware Version: 1.50.2182 (May 11 2011)
 Checksum: 0xc09b9cb2
 Serial Number: J08UPT460M
 Lamp On/Off Event: No
 Lamp Life: 798.00
 Lamp Serial Number: 000296721
 Flow Cell Type: Other
 Flow Cell Path Length: 0.00 mm
 Flow Cell Volume: 0.00 microliters
 Flow Cell Serial Number: 1
 Flow Cell Part Number: 1
 Optics Temperature Stabilzation Setting: unknown
----------------------------  oOo  ----------------------------
------------------- Waters ACQUITY QSM Postrun Report  ---------------
 Firmware Version: 1.50.237 (May 18 2011)
 Software Version: 1.50.1621
 Checksum: 0xae400516
 Serial Number: M09QSM056N
 Minimum System Pressure: 0.0 psi
 Maximum System Pressure: 0.0 psi
 Average System Pressure: 0.0 psi
----------------------------  oOo  ----------------------------
Function 1
Scans in function:          337
Cycle time (secs):          Automatic
Inter Scan Delay (secs):    Automatic
Inter Channel Delay (secs):Automatic
Span (Da):                  0.500
Start and End Time(mins):    0.300 to 1.800
Ionization mode:            ES+
Data type:                  SIR or MRM data
Function type:              MRM of 1 channel
Chan Reaction               Dwell(secs) Cone Volt. Col.Energy Delay(secs) Compound Formula|Mass Comm
ents
1  : 419.87 > 295.05           0.260       30.0       18.0       Auto      EA       418.9        Inte
lliStart Generated
Function 2
Scans in function:          2161
Function type:              Diode Array
Wavelength range (nm):       214 to 214
```

Dataset: Untitled
Last Altered: Tuesday, August 10, 2021 16:06:43 Romance Daylight Time
Printed: Tuesday, August 10, 2021 16:07:40 Romance Daylight Time

Method: C:\MassLynx_Projects\Medchem SU.PRO\MethDB\A.mdb 11 Jan 2021 11:59:51
Calibration: 10 Aug 2021 16:06:43

Compound name: A
Correlation coefficient: r = 0.992787, r^2 = 0.985626
Calibration curve: 17.6488 * x + -329.92
Response type: External Std, Area
Curve type: Linear, Origin: Exclude, Weighting: 1/x, Axis trans: None

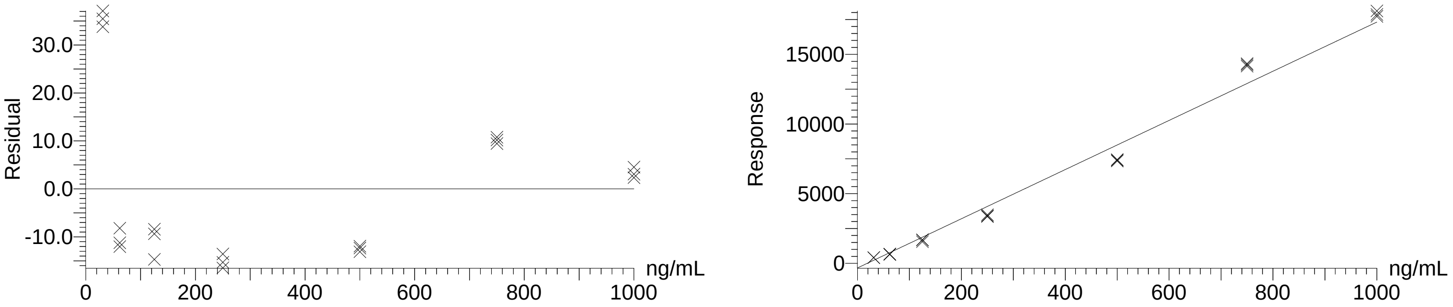

Dataset: Untitled
Last Altered: Tuesday, August 10, 2021 16:06:43 Romance Daylight Time
Printed: Tuesday, August 10, 2021 16:07:40 Romance Daylight Time

Method: C:\MassLynx_Projects\Medchem SU.PRO\MethDB\A.mdb 11 Jan 2021 11:59:51
Calibration: 10 Aug 2021 16:06:43

Sample Name: A MP 0809 31-1-1

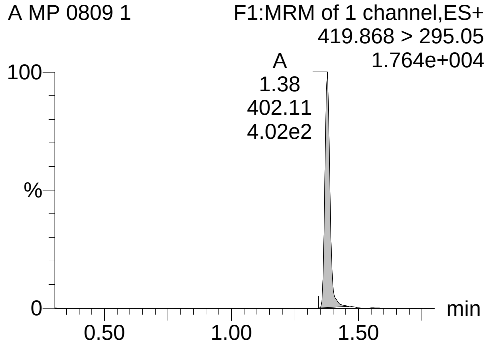

Sample Name: A MP 0809 31-1-2

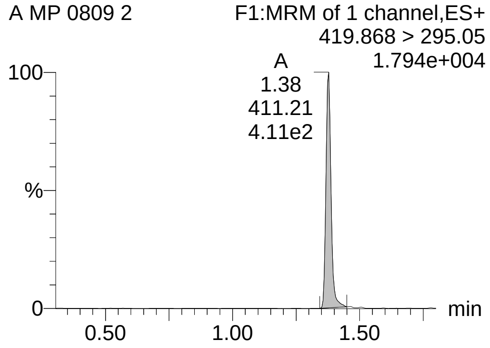

Sample Name: A MP 0809 31-1-3

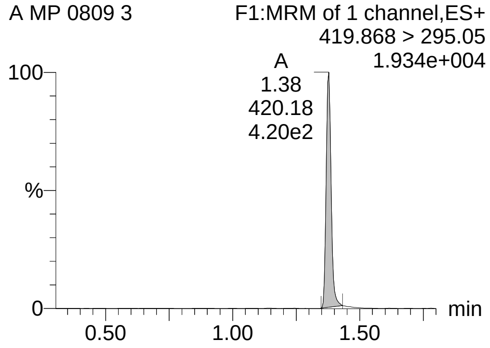

Sample Name: A MP 0809 62-1-1

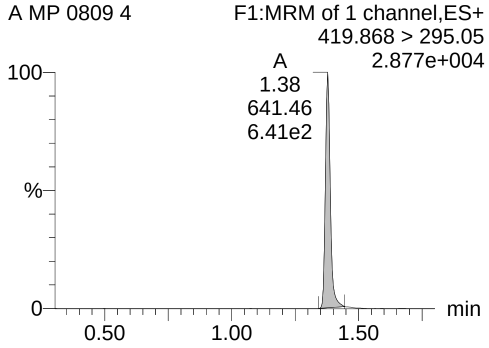

Sample Name: A MP 0809 62-1-2

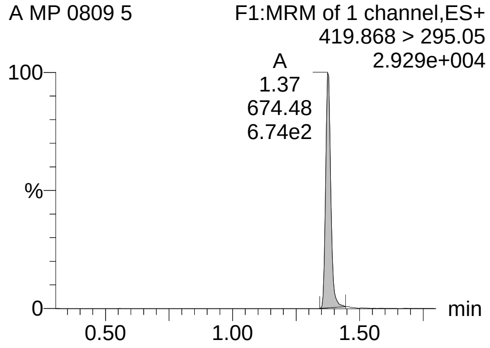

Sample Name: A MP 0809 62-1-3

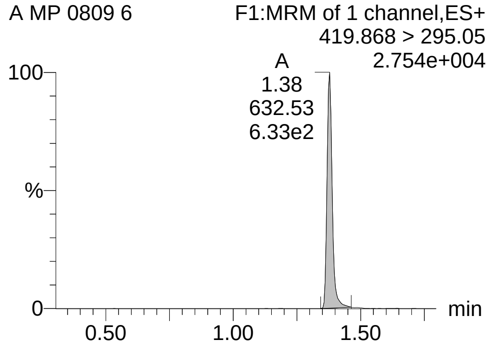

Sample Name: A MP 0809 125-1-1

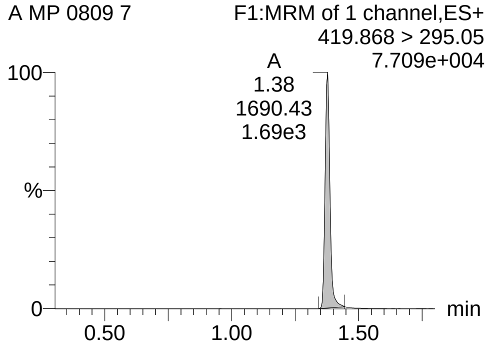

Sample Name: A MP 0809 125-1-2

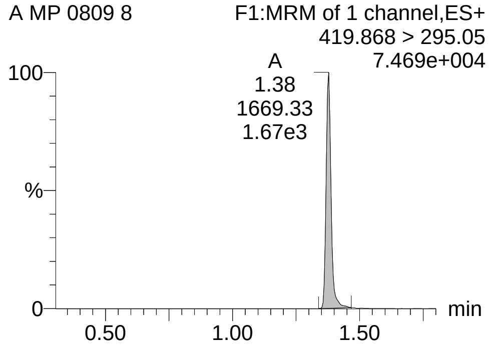

Sample Name: A MP 0809 125-1-3

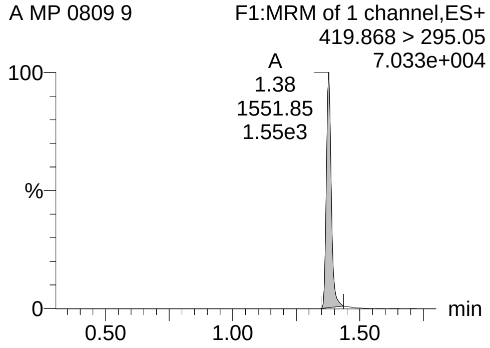

Sample Name: A MP 0809 250-1-1

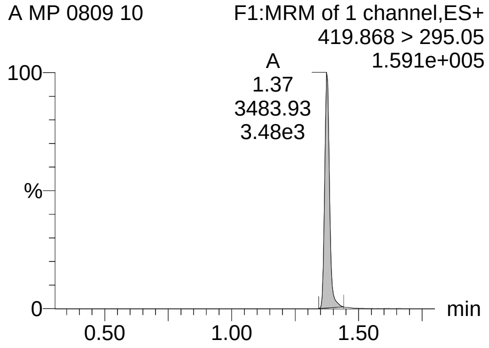

Sample Name: A MP 0809 250-1-2

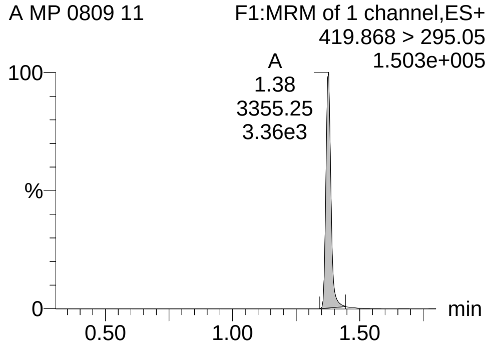

Sample Name: A MP 0809 250-1-3

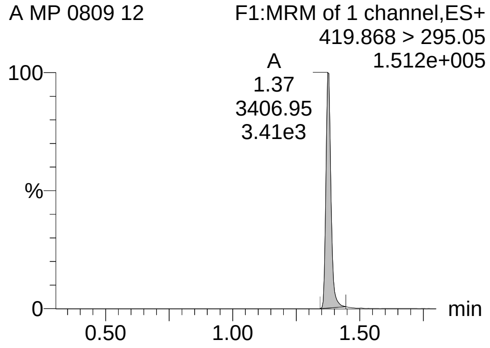

Sample Name: A MP 0809 500-1-1

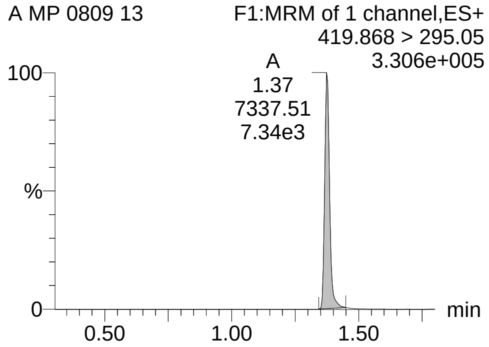

Sample Name: A MP 0809 500-1-2

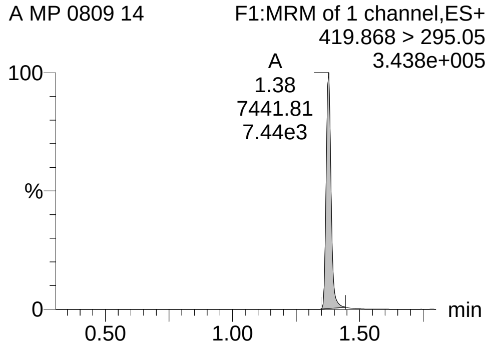

Sample Name: A MP 0809 500-1-3

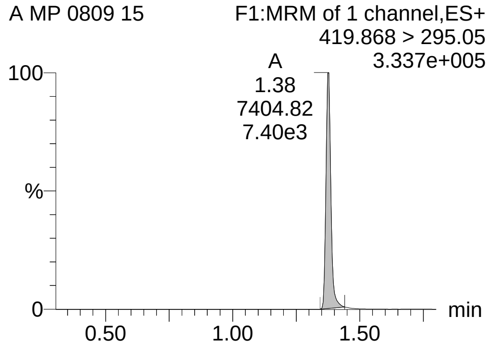

Sample Name: A MP 0809 750-1-1

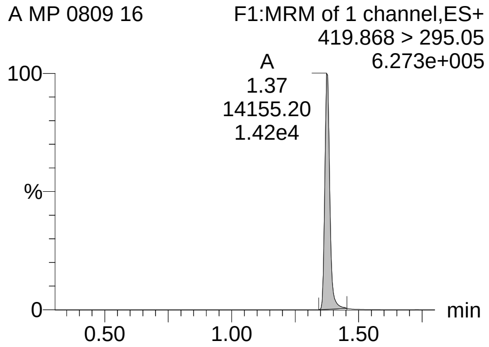

Sample Name: A MP 0809 750-1-2

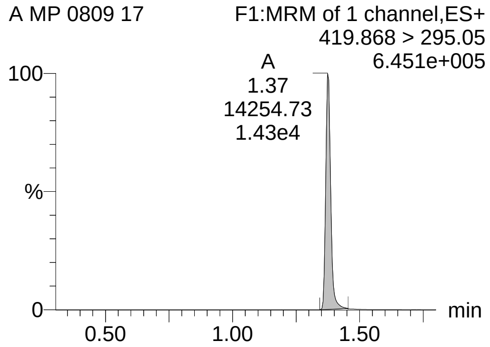

Sample Name: A MP 0809 750-1-3

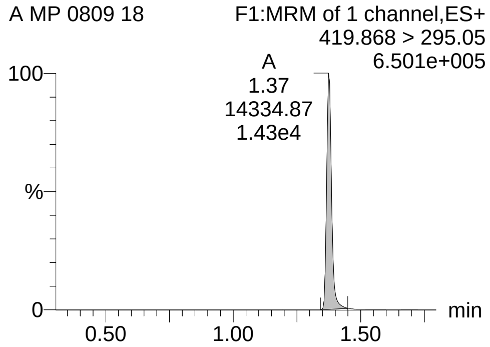

Sample Name: A MP 0809 1000-1-1

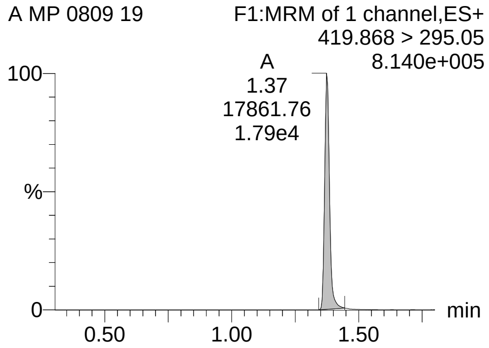

Sample Name: A MP 0809 1000-1-2

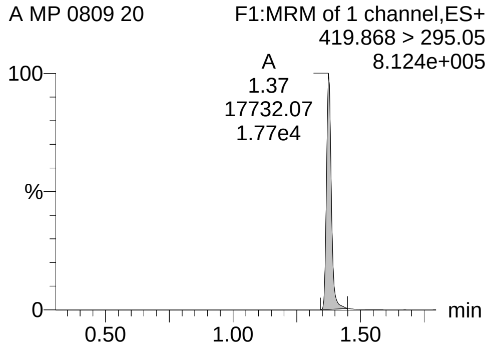

Sample Name: A MP 0809 1000-1-3

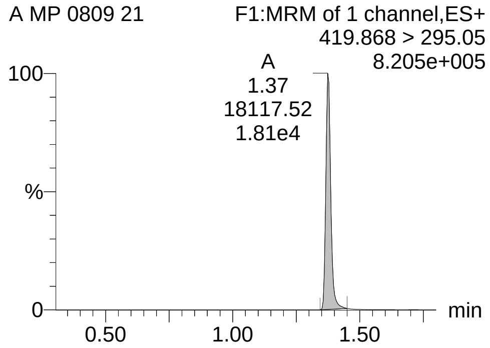

Sample Name: A MP 0809 Blank 1

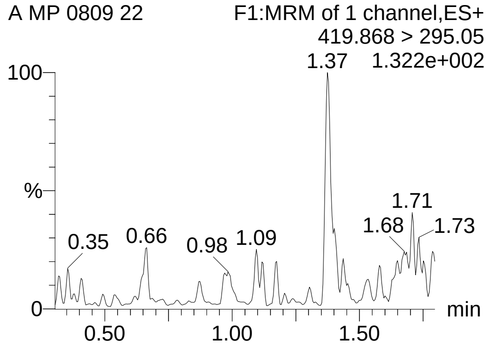

Sample Name: A MP 0809 0-1-1

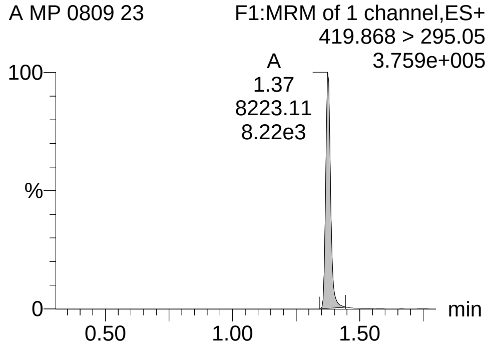

Sample Name: A MP 0809 0-1-2

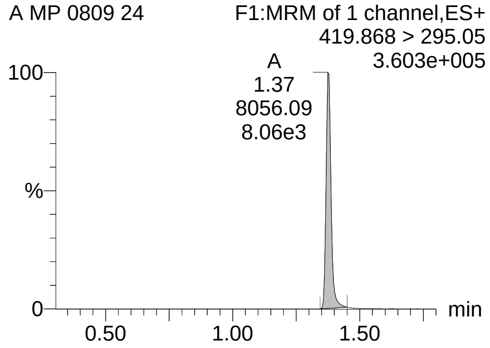

Sample Name: A MP 0809 0-1-3

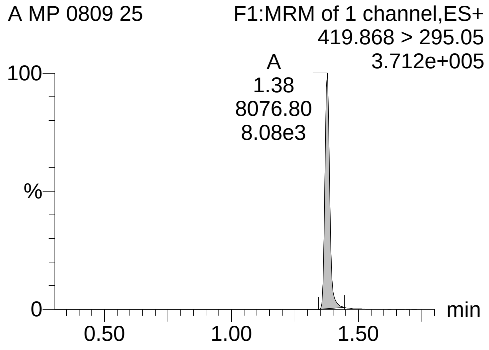

Sample Name: A MP 0809 0-2-1

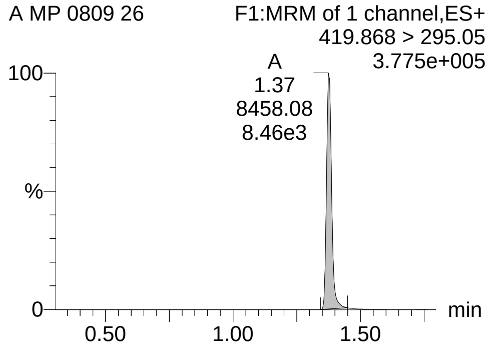

Sample Name: A MP 0809 0-2-2

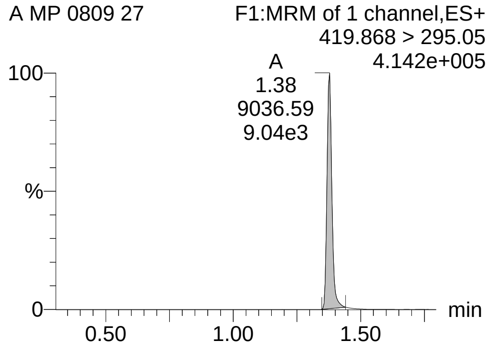

Sample Name: A MP 0809 0-2-3
A MP 0809 28 F1:MRM of 1 channel,ES+
419.868 > 295.05
3.954e+005

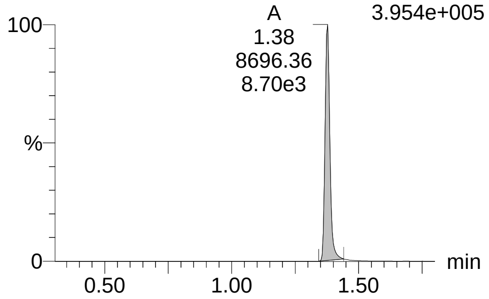

Sample Name: A MP 0809 Blank 2
A MP 0809 29 F1:MRM of 1 channel,ES+
419.868 > 295.05
3.933e+001

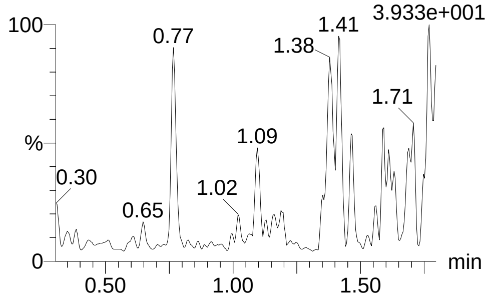

Sample Name: A MP 0809 30-1-1
A MP 0809 30 F1:MRM of 1 channel,ES+
419.868 > 295.05
4.096e+005

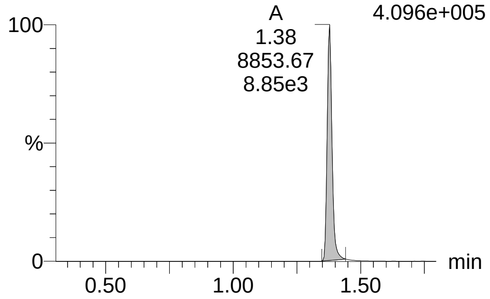

Sample Name: A MP 0809 30-1-2
A MP 0809 31 F1:MRM of 1 channel,ES+
419.868 > 295.05
4.239e+005

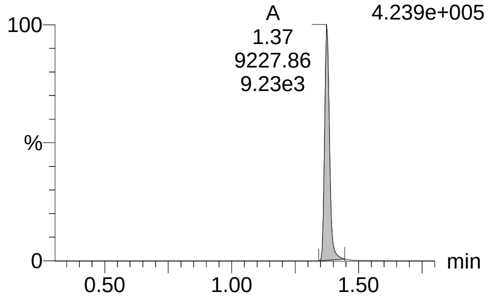

Sample Name: A MP 0809 30-1-3
A MP 0809 32 F1:MRM of 1 channel,ES+
419.868 > 295.05
4.127e+005

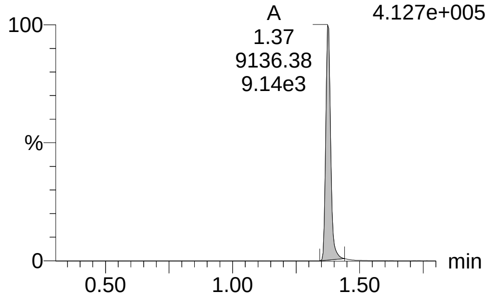

Sample Name: A MP 0809 30-2-1
A MP 0809 33 F1:MRM of 1 channel,ES+
419.868 > 295.05
3.776e+005

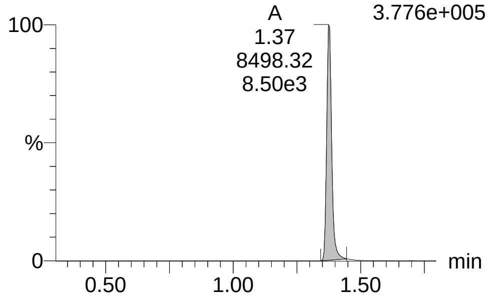

Sample Name: A MP 0809 30-2-2
A MP 0809 34 F1:MRM of 1 channel,ES+
419.868 > 295.05
4.044e+005

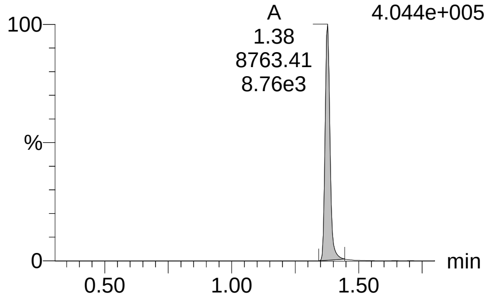

Sample Name: A MP 0809 30-2-3
A MP 0809 35 F1:MRM of 1 channel,ES+
419.868 > 295.05
3.925e+005

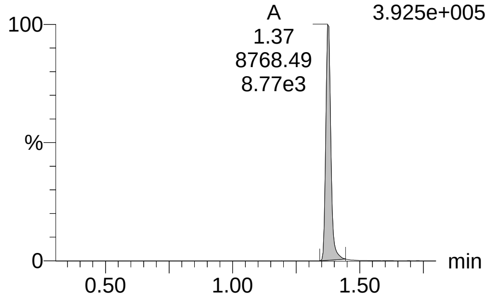

Sample Name: A MP 0809 Blank 3
A MP 0809 36 F1:MRM of 1 channel,ES+
419.868 > 295.05
5.289e+001

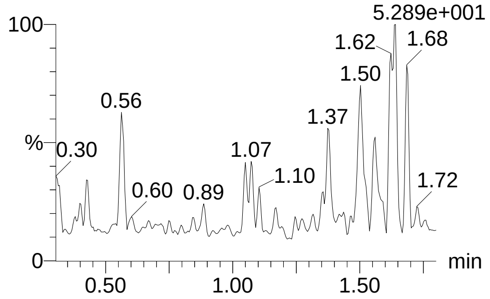

Sample Name: A MP 0809 1-1-1
A MP 0809 37 F1:MRM of 1 channel,ES+
419.868 > 295.05
3.937e+005

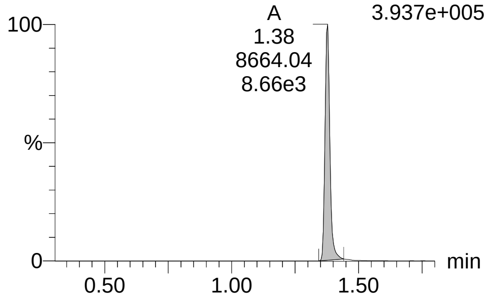

Sample Name: A MP 0809 1-1-2
A MP 0809 38 F1:MRM of 1 channel,ES+
419.868 > 295.05
3.918e+005

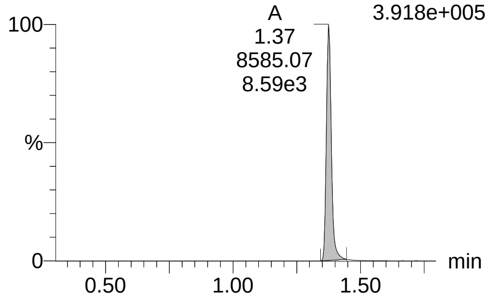

Sample Name: A MP 0809 1-1-3
A MP 0809 39 F1:MRM of 1 channel,ES+
419.868 > 295.05
4.008e+005

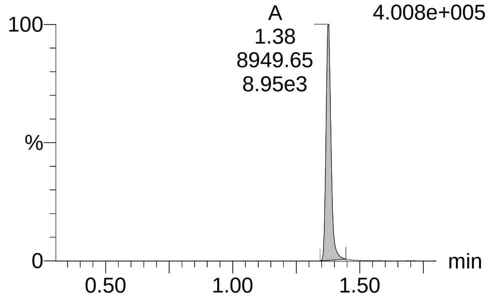

Sample Name: A MP 0809 1-2-1
A MP 0809 40 F1:MRM of 1 channel,ES+
419.868 > 295.05
3.846e+005

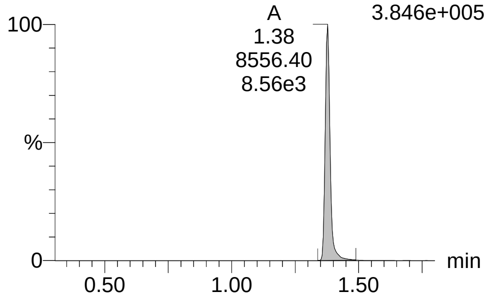

Sample Name: A MP 0809 1-2-2
A MP 0809 41 F1:MRM of 1 channel,ES+
419.868 > 295.05
3.701e+005

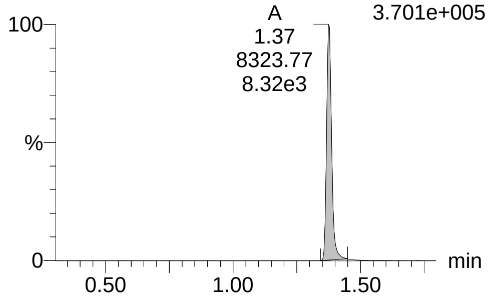

Sample Name: A MP 0809 1-2-3
A MP 0809 42 F1:MRM of 1 channel,ES+
419.868 > 295.05
3.757e+005

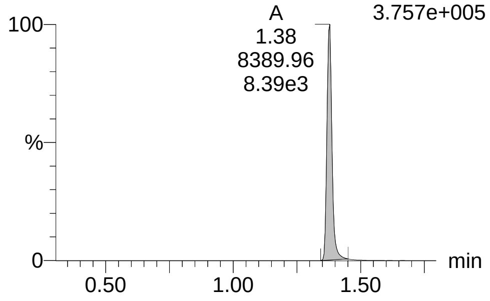

Sample Name: A MP 0809 Blank 4

A MP 0809 43 F1:MRM of 1 channel,ES+
419.868 > 295.05

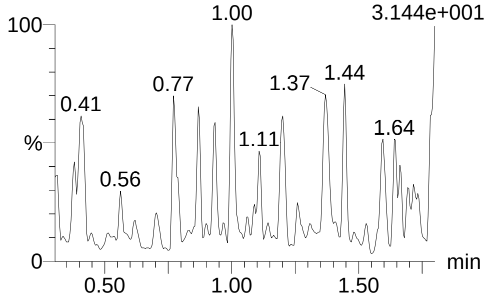

Sample Name: A MP 0809 2-1-1

A MP 0809 44 F1:MRM of 1 channel,ES+
419.868 > 295.05

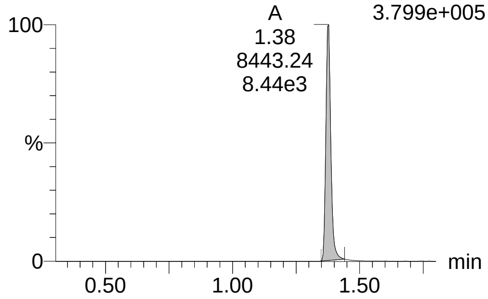

Sample Name: A MP 0809 2-1-2

A MP 0809 45 F1:MRM of 1 channel,ES+
419.868 > 295.05

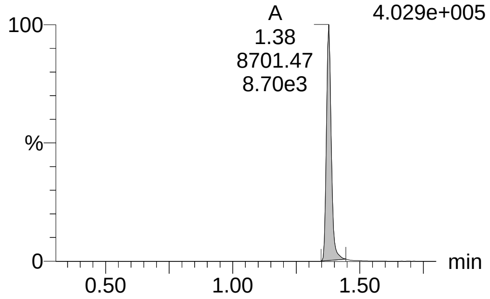

Sample Name: A MP 0809 2-1-3

A MP 0809 46 F1:MRM of 1 channel,ES+
419.868 > 295.05

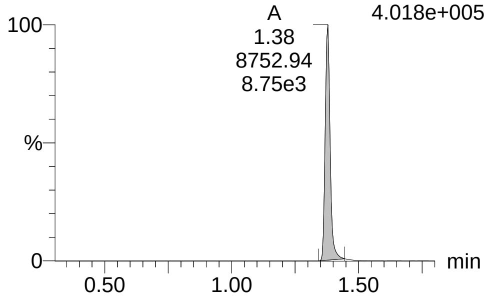

Sample Name: A MP 0809 2-2-1

A MP 0809 47 F1:MRM of 1 channel,ES+
419.868 > 295.05

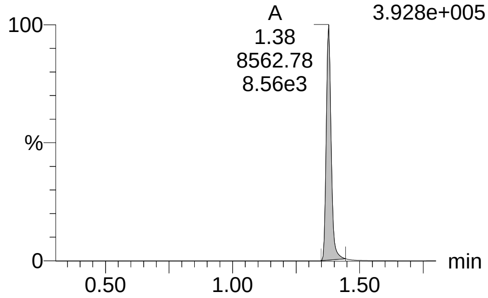

Sample Name: A MP 0809 2-2-2

A MP 0809 48 F1:MRM of 1 channel,ES+
419.868 > 295.05

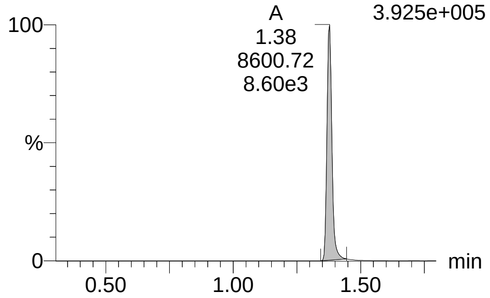

Sample Name: A MP 0809 2-2-3

A MP 0809 49 F1:MRM of 1 channel,ES+
419.868 > 295.05

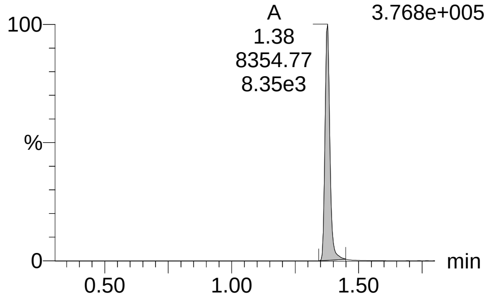

Sample Name: A MP 0809 Blank 5

A MP 0809 50 F1:MRM of 1 channel,ES+
419.868 > 295.05

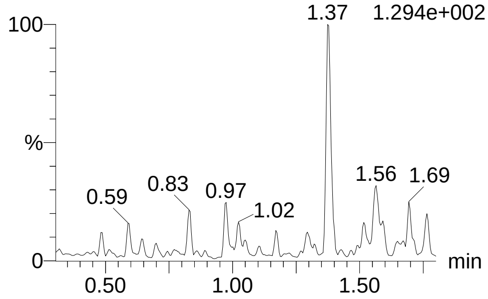

Sample Name: A MP 0809 3-1-1

A MP 0809 51 F1:MRM of 1 channel,ES+
419.868 > 295.05

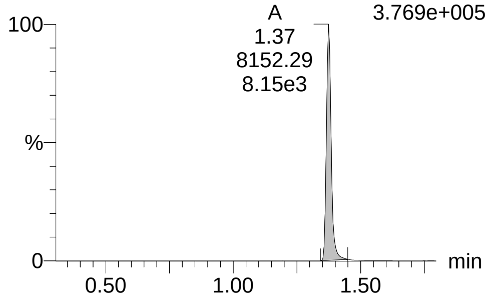

Sample Name: A MP 0809 3-1-2

A MP 0809 52 F1:MRM of 1 channel,ES+
419.868 > 295.05

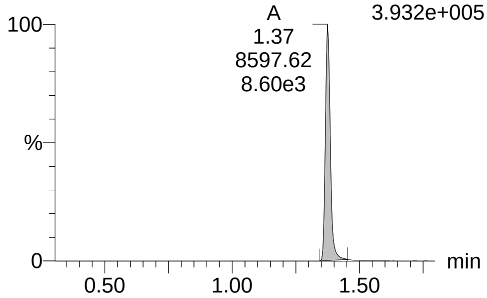

Sample Name: A MP 0809 3-1-3

A MP 0809 53 F1:MRM of 1 channel,ES+
419.868 > 295.05

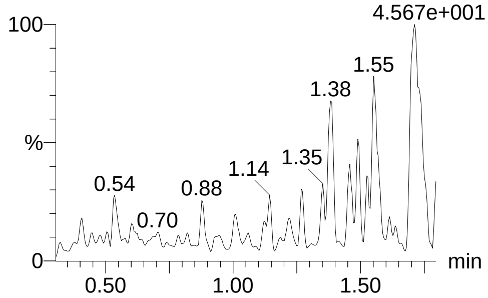

Sample Name: A MP 0809 3-2-1

A MP 0809 54 F1:MRM of 1 channel,ES+
419.868 > 295.05

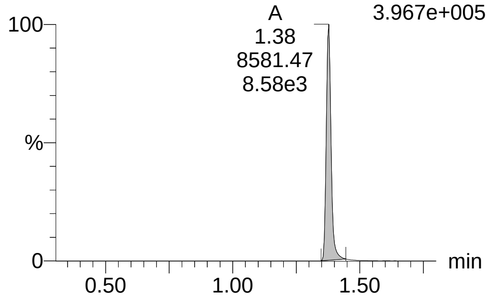

Sample Name: A MP 0809 3-2-2

A MP 0809 55 F1:MRM of 1 channel,ES+
419.868 > 295.05

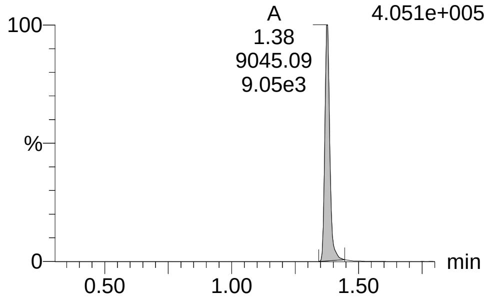

Sample Name: A MP 0809 3-2-3

A MP 0809 56 F1:MRM of 1 channel,ES+
419.868 > 295.05

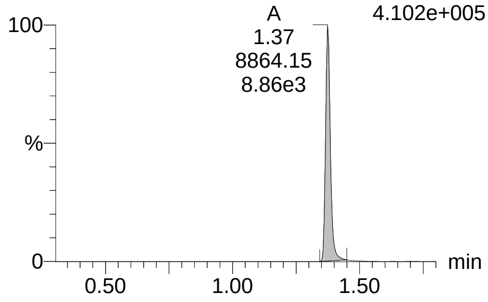

Sample Name: A MP 0809 Blank 6

A MP 0809 57 F1:MRM of 1 channel,ES+
419.868 > 295.05

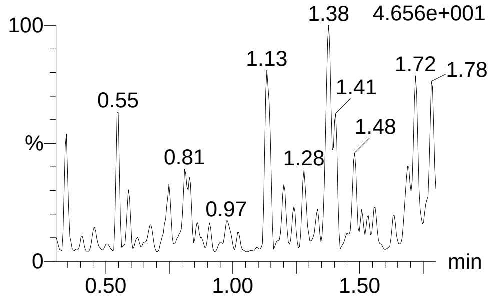

Sample Name: A MP 0809 6-1-1

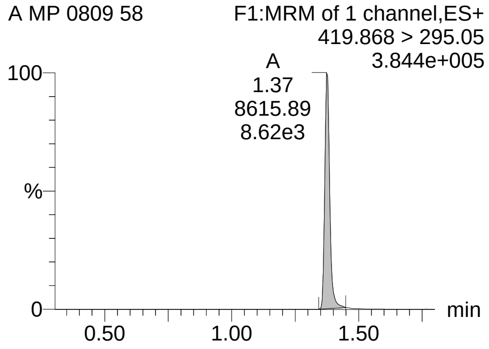

Sample Name: A MP 0809 6-1-2

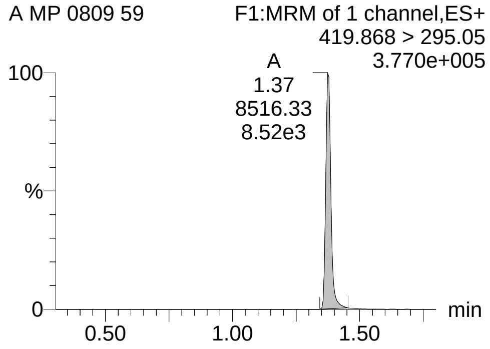

Sample Name: A MP 0809 6-1-3

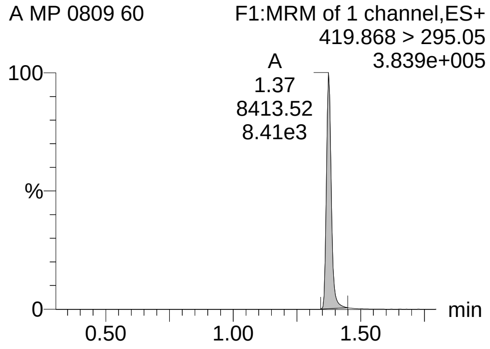

Sample Name: A MP 0809 6-2-1

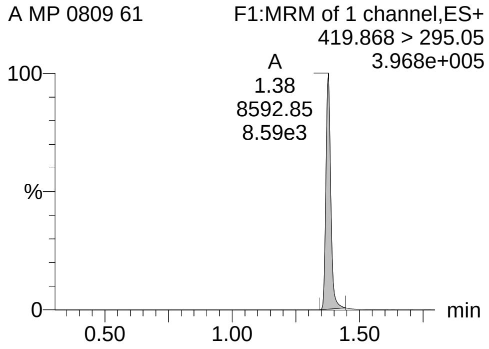

Sample Name: A MP 0809 6-2-2

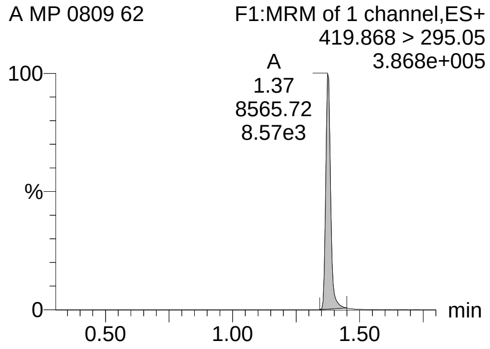

Sample Name: A MP 0809 6-2-3

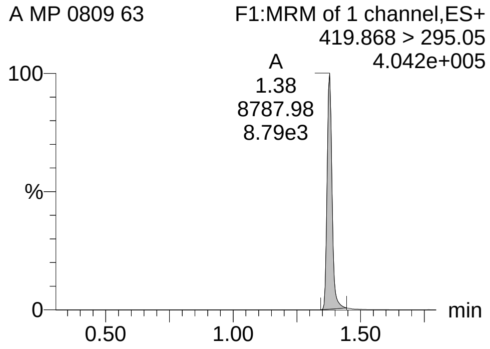

	#	Name	Type	Std. Conc	RT	Area	IS Area	Response	Detecti...	ng/mL	%Dev
1	1	A MP 0809 31-1-1	Stan...	31.000	1.38	402.107		402.107	bb	41.5	33.8
2	2	A MP 0809 31-1-2	Stan...	31.000	1.38	411.210		411.210	bb	42.0	35.5
3	3	A MP 0809 31-1-3	Stan...	31.000	1.38	420.178		420.178	bb	42.5	37.1
4	4	A MP 0809 62-1-1	Stan...	62.000	1.38	641.458		641.458	bb	55.0	-11.2
5	5	A MP 0809 62-1-2	Stan...	62.000	1.37	674.476		674.476	bb	56.9	-8.2
6	6	A MP 0809 62-1-3	Stan...	62.000	1.38	632.529		632.529	bb	54.5	-12.0
7	7	A MP 0809 125-1-1	Stan...	125.000	1.38	1690.431		1690.431	bb	114.5	-8.4
8	8	A MP 0809 125-1-2	Stan...	125.000	1.38	1669.333		1669.333	bb	113.3	-9.4
9	9	A MP 0809 125-1-3	Stan...	125.000	1.38	1551.851		1551.851	bb	106.6	-14.7
10	10	A MP 0809 250-1-1	Stan...	250.000	1.37	3483.929		3483.929	bb	216.1	-13.6
11	11	A MP 0809 250-1-2	Stan...	250.000	1.38	3355.248		3355.248	bb	208.8	-16.5
12	12	A MP 0809 250-1-3	Stan...	250.000	1.37	3406.949		3406.949	bb	211.7	-15.3
13	13	A MP 0809 500-1-1	Stan...	500.000	1.37	7337.505		7337.505	bb	434.4	-13.1
14	14	A MP 0809 500-1-2	Stan...	500.000	1.38	7441.806		7441.806	bb	440.4	-11.9
15	15	A MP 0809 500-1-3	Stan...	500.000	1.38	7404.821		7404.821	bb	438.3	-12.3
16	16	A MP 0809 750-1-1	Stan...	750.000	1.37	14155.195		14155.195	bb	820.7	9.4
17	17	A MP 0809 750-1-2	Stan...	750.000	1.37	14254.726		14254.726	bb	826.4	10.2
18	18	A MP 0809 750-1-3	Stan...	750.000	1.37	14334.868		14334.868	bb	830.9	10.8
19	19	A MP 0809 1000-1-1	Stan...	1000.000	1.37	17861.760		17861.760	bb	1030.8	3.1
20	20	A MP 0809 1000-1-2	Stan...	1000.000	1.37	17732.070		17732.070	bb	1023.4	2.3
21	21	A MP 0809 1000-1-3	Stan...	1000.000	1.37	18117.516		18117.516	bb	1045.3	4.5
22	22	A MP 0809 Blank 1	Blank								
23	23	A MP 0809 0-1-1	Anal...		1.37	8223.114		8223.114	bb	484.6	
24	24	A MP 0809 0-1-2	Anal...		1.37	8056.094		8056.094	bb	475.2	
25	25	A MP 0809 0-1-3	Anal...		1.38	8076.796		8076.796	bb	476.3	
26	26	A MP 0809 0-2-1	Anal...		1.37	8458.085		8458.085	bb	497.9	
27	27	A MP 0809 0-2-2	Anal...		1.38	9036.594		9036.594	bb	530.7	
28	28	A MP 0809 0-2-3	Anal...		1.38	8696.356		8696.356	bb	511.4	
29	29	A MP 0809 Blank 2	Blank								
30	30	A MP 0809 30-1-1	Anal...		1.38	8853.674		8853.674	bb	520.4	
31	31	A MP 0809 30-1-2	Anal...		1.37	9227.857		9227.857	bb	541.6	
32	32	A MP 0809 30-1-3	Anal...		1.37	9136.378		9136.378	bb	536.4	
33	33	A MP 0809 30-2-1	Anal...		1.37	8498.318		8498.318	bb	500.2	
34	34	A MP 0809 30-2-2	Anal...		1.38	8763.412		8763.412	bb	515.2	
35	35	A MP 0809 30-2-3	Anal...		1.37	8768.485		8768.485	bb	515.5	
36	36	A MP 0809 Blank 3	Blank								
37	37	A MP 0809 1-1-1	Anal...		1.38	8664.037		8664.037	bb	509.6	
38	38	A MP 0809 1-1-2	Anal...		1.37	8585.073		8585.073	bb	505.1	
39	39	A MP 0809 1-1-3	Anal...		1.38	8949.646		8949.646	bb	525.8	
40	40	A MP 0809 1-2-1	Anal...		1.38	8556.404		8556.404	bb	503.5	
41	41	A MP 0809 1-2-2	Anal...		1.37	8323.767		8323.767	bb	490.3	
42	42	A MP 0809 1-2-3	Anal...		1.38	8389.962		8389.962	bb	494.1	
43	43	A MP 0809 Blank 4	Blank								
44	44	A MP 0809 2-1-1	Anal...		1.38	8443.240		8443.240	bb	497.1	
45	45	A MP 0809 2-1-2	Anal...		1.38	8701.473		8701.473	bb	511.7	
46	46	A MP 0809 2-1-3	Anal...		1.38	8752.936		8752.936	bb	514.6	
47	47	A MP 0809 2-2-1	Anal...		1.38	8562.783		8562.783	bb	503.9	
48	48	A MP 0809 2-2-2	Anal...		1.38	8600.715		8600.715	bb	506.0	
49	49	A MP 0809 2-2-3	Anal...		1.38	8354.774		8354.774	bb	492.1	
50	50	A MP 0809 Blank 5	Blank								
51	51	A MP 0809 3-1-1	Anal...		1.37	8152.294		8152.294	bb	480.6	
52	52	A MP 0809 3-1-2	Anal...		1.37	8597.622		8597.622	bb	505.8	
53	53	A MP 0809 3-1-3	Anal...								
54	54	A MP 0809 3-2-1	Anal...		1.38	8581.473		8581.473	bb	504.9	
55	55	A MP 0809 3-2-2	Anal...		1.38	9045.090		9045.090	bb	531.2	
56	56	A MP 0809 3-2-3	Anal...		1.37	8864.148		8864.148	bb	520.9	
57	57	A MP 0809 Blank 6	Blank								
58	58	A MP 0809 6-1-1	Anal...		1.37	8615.894		8615.894	bb	506.9	

	#	Name	Type	Std. Conc	RT	Area	IS Area	Response	Detecti...	ng/mL	%Dev
59	59	A MP 0809 6-1-2	Anal...		1.37	8516.327		8516.327	bb	501.2	
60	60	A MP 0809 6-1-3	Anal...		1.37	8413.519		8413.519	bb	495.4	
61	61	A MP 0809 6-2-1	Anal...		1.38	8592.851		8592.851	bb	505.6	
62	62	A MP 0809 6-2-2	Anal...		1.37	8565.722		8565.722	bb	504.0	
63	63	A MP 0809 6-2-3	Anal...		1.38	8787.980		8787.980	bb	516.6	

Method: C:\MassLynx_Projects\Medchem SU.PRO\MethDB\A.mdb 11 Jan 2021 11:59:51
Calibration: 10 Aug 2021 16:06:43

```
Header
Acquired File Name:   A MP 0809 31-1-1
Acquired Date:        10-Aug-2021
Acquired Time:        02:07:30
Job Code:             PSMP - 20210809 - N - 216 - 075 - A
Task Code:
User Name:
Laboratory Name:
Instrument:           ACQ-TQD#QBA320
Conditions:
Submitter:
SampleID:             A MP 0809 1
Bottle Number:        2:79
Description:
Instrument Calibration:
Calibration File: C:\MassLynx\IntelliStart\Results\Unit Mass Resolution\Calib 20161209.cal
Parameters
MS1 Static:
Mass:             20 Da to 1974 Da.
Resolution:       15.0/15.0
Ion Energy:       0.5
Reference File:   Naics2
Acquisition File: STATMS1
MS1 Scanning:
Mass:             15 Da to 2048 Da.
Resolution:       15.0/15.0
Ion Energy:       0.5
Reference File:   Naics2
Acquisition File: SCNMS1
MS1 Scan Speed Compensation:
Scan:             339 to 2000 amu/sec.
Resolution:       15.0/15.0
Ion Energy:       0.5
Reference File:   Naics2
Acquisition File: FASTMS1
MS2 Static:
Mass:             20 Da to 1974 Da.
Resolution:       15.0/15.0
Ion Energy:       0.5
Reference File:   Naics2
Acquisition File: STATMS2
MS2 Scanning:
Mass:             15 Da to 2048 Da.
Resolution:       15.0/15.0
Ion Energy:       0.5
Reference File:   Naics2
Acquisition File: SCNMS2
MS2 Scan Speed Compensation:
Scan:             339 to 10165 amu/sec.
Resolution:       15.0/15.0
Ion Energy:       0.5
Reference File:   Naics2
Acquisition File: FASTMS2
Calibration Time: 10:33
Calibration Date: 12/09/16
Coefficients
MS1 Static:       -0.000000000000*x^4 + 0.000000000843*x^3 + -0.000001360559*x^2 + 1.000940122634*x
 +-0.253266439024
MS2 Static:       -0.000000000000*x^4 + 0.000000000573*x^3 + -0.000001239648*x^2 + 1.000902600483*x
 +-0.192715155436
Function 1:       None
Function 2:       None
Parameters for C:\Documents and Settings\Administrator\Desktop\old desktop\log D\EA.EXP
Data Processing:
SIR / MRM Chromatogram Spike Removal      ON
```

```
SIR / MRM Smoothing           OFF
Smoothing window size (scans) 3
Number of smooths       2
Prescan Statistics:
Initial Average Intensity     21.8092
Initial Average Std Dev       1.7357
Bunch Zero Level        0.0135
Bunch Std Dev                 0.0170
Bunch Threshold               0.1363
Spike Removal Std Dev         1.7258
Method Events:
Initial Stop Flow:      No Change
Initial Switch 2: No Change
Initial Switch 3: No Change
Initial Switch 4: No Change
Initial Infusion: No Change
Initial Flow State:     LC
Initial Flow Rate:      5
Initial Reservoir:      No Action
API Probe Delay Temp:   20
Initial Refill:         No Action
Timed Events Enabled
Event Time  Name        Action
Instrument Parameters - Function 1:
Parameter File - C:\Documents and Settings\Administrator\Desktop\old desktop\log D\log D tune file.
IPR
Polarity    ES+
Calibration Static 2
Capillary (kV)    3.50  3.48
Cone (V)    38.00 28.57
Extractor (V)     3.00  2.20
RF (V)      0.10
Source Temperature (°C) 140   139
Desolvation Temperature (°C)  450   450
Cone Gas Flow (L/Hr)    90    90
Desolvation Gas Flow (L/Hr)   900   901
Collision Gas Flow (mL/Min)   0.20  0.20
LM 1 Resolution   15.50
HM 1 Resolution   14.84
Ion Energy 1      0.30
MS Mode Entrance  50.00
MS Mode Collision Energy      3.00
MS Mode Exit      50.00
MSMS Mode Entrance      -2.00
MSMS Mode Collision Energy    2.00
MSMS Mode Exit    2.00
LM 2 Resolution   15.00
HM 2 Resolution   15.50
Ion Energy 2      1.22
Gain  1.00
Multiplier  -493.82
Active Reservoir  B
Engineers Settings:
MS1 Low Mass Position         518
MS1 High Mass Position        284
MS1 Low Mass Resolution       513
MS1 High Mass Resolution      1732
MS1 Resolution Linearity      834
MS1 High Mass DC Balance      0
MS1 DC Polarity               Positive
MS2 Low Mass Position         519
MS2 High Mass Position        238
MS2 Low Mass Resolution       518
MS2 High Mass Resolution      644
MS2 Resolution Linearity      756
MS2 High Mass DC Balance      -0
MS2 DC Polarity               Positive
HM RF Lens Correction +       0
```

```
HM RF Lens Correction -       0
Inter-scan delays:
Automatic Mode
MS 1 Delay Table:
         R     delay
<=   0.500     0.005
<=   1.200     0.010
<=   2.400     0.015
<=   6.000     0.020
<=  15.000     0.025
<=  25.000     0.028
>   25.000     0.030
MS 2 Delay Table:
         R     delay
<=   2.000     0.005
<=   4.000     0.008
<=   7.000     0.010
<=  10.000     0.012
<=  20.000     0.014
>   20.000     0.016
ACE Experimental Record
Inlet Method File: c:\masslynx_projects\medchem su.pro\acqudb\adme sophie
-------------------   Prerun method parameters   ---------------
Waters ACQUITY QSM
Waters Acquity TUV
 Run Time: 0.20 min
 Wavelength Mode: Single Wavelength
 Lamp On: On
Channel A...
 Comment:
 Wavelength: 254 nm
 Sampling Rate: 20 points/sec
 Data Mode: Absorbance
 Time Constant: 0.1000 sec
 Auto Zero On Wavelength Change: Maintain Baseline
 Auto Zero On Inject Start: Yes
Analog 1...
 Sensitivity: 2.000 AUFS
 Chart Polarity: Positive (+)
 Voltage Offset: 0 mV
 Enable Chart Mark: Yes
 Run Events: Yes
 Pulse Width: 1.0 sec
 Rect Wave Period: 0.2 sec
----------------------------  oOo  ---------------------------
---------------------   Run method parameters   ----------------
Waters ACQUITY QSM
 Solvent A Name: Water
 Solvent B Name: Acetonitrile
 Solvent C Name: water 1 % FA
 Solvent D Name:
 Low Pressure Limit: 0 psi
 High Pressure Limit: 15000 psi
 Seal Wash Period: 5.00 min
 [Gradient Table]
  Time(min) Flow Rate(mL/min) %A %B %C %D Curve
 1. Initial 0.700 90.0 5.0 5.0 0.0 Initial
 2. 0.15 0.700 45.0 50.0 5.0 0.0 6
 3. 1.50 0.700 0.0 95.0 5.0 0.0 6
 4. 1.80 0.700 90.0 5.0 5.0 0.0 1
 Comment: ACQUITY UPLC BEH C18 2.1 x 50 mm
 Flow Ramp Rate: 0.45 min
 D Solvent Selection (if supported): No Change
 System Pressure Data Channel: No
 Flow Rate Data Channel: No
 %A Data Channel: No
 %B Data Channel: No
 %C Data Channel: No
```

```
 %D Data Channel: No
 Primary Data Channel: No
 Accumulator Data Channel: No
 Degasser Data Channel: No
 Gradient Start: At Injection
 Gradient Start Volume: 0 uL
 Gradient Start Time: 0.00 min
 Participate in pre-analysis: No
Waters Acquity TUV
 Run Time: 1.80 min
 Wavelength Mode: Single Wavelength
 Lamp On: On
Channel A...
 Comment:
 Wavelength: 214 nm
 Sampling Rate: 20 points/sec
 Data Mode: Absorbance
 Time Constant: 0.1000 sec
 Auto Zero On Wavelength Change: Maintain Baseline
 Auto Zero On Inject Start: Yes
Analog 1...
 Sensitivity: 2.000 AUFS
 Chart Polarity: Positive (+)
 Voltage Offset: 0 mV
 Enable Chart Mark: Yes
 Run Events: Yes
 Pulse Width: 1.0 sec
 Rect Wave Period: 0.2 sec
Waters ACQUITY FTN AutoSampler
 Run Time: 1.80 min
 Comment: ACQUITY UPLC BEH C18 2.1 x 50 mm
 Load Ahead: Disabled
 Loop Offline: Automatic min
 Wash Solvent Name: Acetonitrile
 Pre-Inject Wash Time: 0.0 sec
 Post-Inject Wash Time: 6.0 sec
 Purge Solvent Name: Water
 Dilution: Disabled
 Dilution Volume: 0 uL
 Delay Time: 0 min
 Dilution Needle Placement: 4.0 mm
 Target Column Temperature: 40.0 C
 Column Temperature Alarm Band: Disabled
 Target Sample Temperature: 15.0 C
 Sample Temperature Alarm Band: Disabled
 Syringe Draw Rate: Automatic
 Needle Placement: Automatic
 Pre-Aspirate Air Gap: Automatic
 Post-Aspirate Air Gap: Automatic
 Column Temperature Data Channel: No
 Room Temperature Data Channel: No
 Sample Temperature Data Channel: No
 Sample Organizer Temperature Data Channel: No
 Sample Pressure Data Channel: No
 Preheater Temperature Data Channel: No
 Seal Force Data Channel: No
 No Injection Mode Enabled: No
 Run Events: No
Sample Run Injection Parameter
Injection Volume (ul)   -   3.00
----------------------------  oOo  -----------------------------
End of experimental record.
-------------------- Waters ACQUITY QSM Postrun Report  ---------------
 Firmware Version: 1.50.237 (May 18 2011)
 Software Version: 1.50.1621
 Checksum: 0xae400516
 Serial Number: M09QSM056N
 Minimum System Pressure: 0.0 psi
```

```
 Maximum System Pressure: 0.0 psi
 Average System Pressure: 0.0 psi
----------------------------  oOo  -----------------------------
------------------- Waters ACQUITY FTN Postrun Report  ---------------
 Software Version: 1.50.1481
 Firmware Version: 1.50.317 (Jul 11 2011)
 Checksum: 0x3e83519d
 Serial Number: M09SDI055N
 Sample Syringe Size: 100.0
 Extension Loop Size: 0.0
 Needle Size: 15.0
 Column Type: ACQUITY UPLC® BEH C18 1.7µm
 Column Serial Number: 02343407715781
 Total Injections on Column: 6739
 Minimum Sample Temperature: 0.0
 Maximum Sample Temperature: 0.0
 Average Sample Temperature: 0.0
 Minimum Column Temperature: 40.0
 Maximum Column Temperature: 40.1
 Average Column Temperature: 0.0
----------------------------  oOo  -----------------------------
------------------- Generic Instrument Postrun Report  ---------------
 Software Version: 1.50.2530
 Firmware Version: 1.50.2182 (May 11 2011)
 Checksum: 0xc09b9cb2
 Serial Number: J08UPT460M
 Lamp On/Off Event: No
 Lamp Life: 823.00
 Lamp Serial Number: 000296721
 Flow Cell Type: Other
 Flow Cell Path Length: 0.00 mm
 Flow Cell Volume: 0.00 microliters
 Flow Cell Serial Number: 1
 Flow Cell Part Number: 1
 Optics Temperature Stabilzation Setting: unknown
----------------------------  oOo  -----------------------------
------------------- Waters ACQUITY QSM Postrun Report  ---------------
 Firmware Version: 1.50.237 (May 18 2011)
 Software Version: 1.50.1621
 Checksum: 0xae400516
 Serial Number: M09QSM056N
 Minimum System Pressure: 0.0 psi
 Maximum System Pressure: 0.0 psi
 Average System Pressure: 0.0 psi
----------------------------  oOo  -----------------------------
Function 1
Scans in function:          338
Cycle time (secs):          Automatic
Inter Scan Delay (secs):    Automatic
Inter Channel Delay (secs):Automatic
Span (Da):                  0.500
Start and End Time(mins):    0.300 to 1.800
Ionization mode:            ES+
Data type:                  SIR or MRM data
Function type:              MRM of 1 channel
Chan Reaction               Dwell(secs) Cone Volt. Col.Energy Delay(secs) Compound Formula|Mass Comm
ents
1  : 419.87 > 295.05           0.260       30.0       18.0       Auto     EA       418.9        Inte
lliStart Generated
Function 2
Scans in function:          2161
Function type:              Diode Array
Wavelength range (nm):       214 to 214
```

Dataset: Untitled
Last Altered: Monday, January 18, 2021 11:54:53 Romance Standard Time
Printed: Monday, January 18, 2021 11:55:44 Romance Standard Time

Method: C:\MassLynx_Projects\Medchem SU.PRO\MethDB\A.mdb 11 Jan 2021 10:59:51
Calibration: 18 Jan 2021 11:54:53

Compound name: A
No Calibration
Response type: External Std, Area
Curve type: Linear, Origin: Exclude, Weighting: 1/x, Axis trans: None

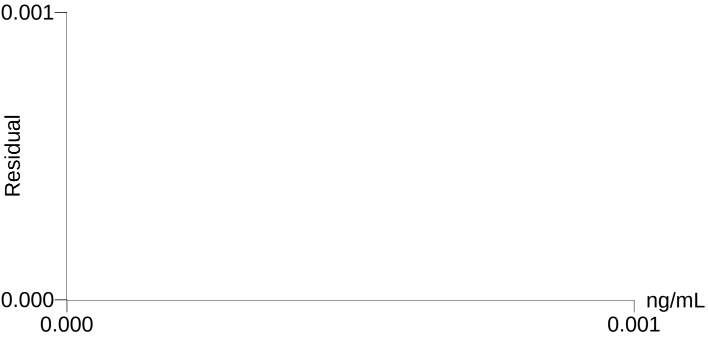

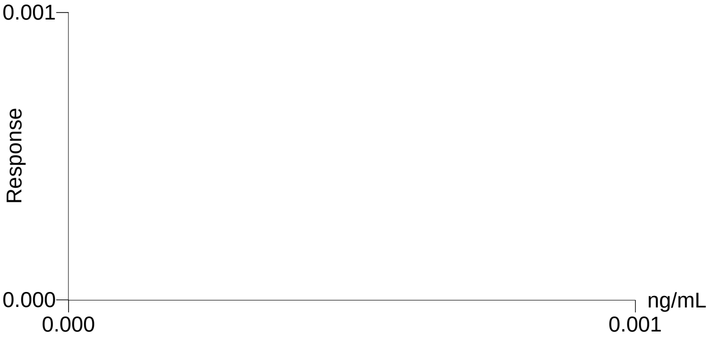

Dataset: Untitled
Last Altered: Monday, January 18, 2021 11:54:53 Romance Standard Time
Printed: Monday, January 18, 2021 11:55:44 Romance Standard Time

Method: C:\MassLynx_Projects\Medchem SU.PRO\MethDB\A.mdb 11 Jan 2021 10:59:51
Calibration: 18 Jan 2021 11:54:53

Sample Name: A MSH 0-1-1

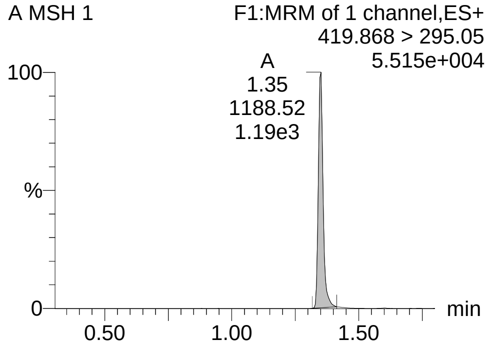

Sample Name: A MSH 0-1-2

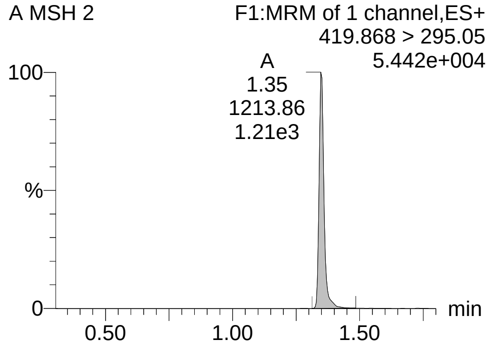

Sample Name: A MSH 0-1-3

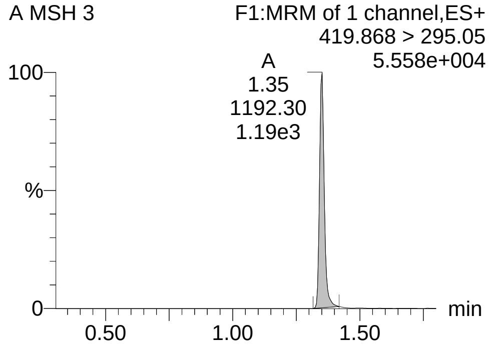

Sample Name: A MSH 0-2-1

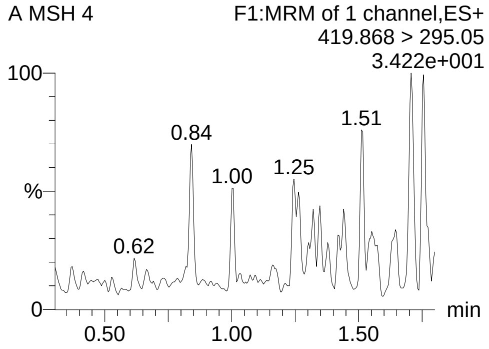

Sample Name: A MSH 0-2-2

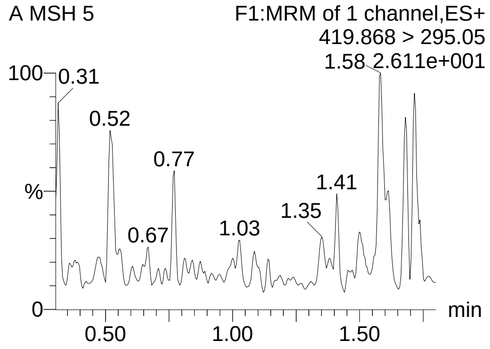

Sample Name: A MSH 0-2-3

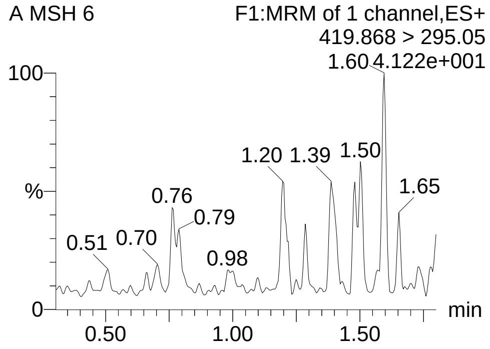

Sample Name: A MSH Blank 1

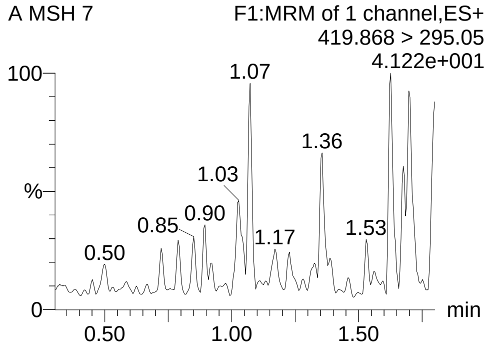

Sample Name: A MSH 15-1-1

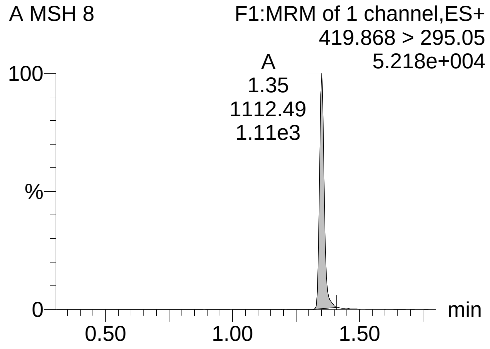

Sample Name: A MSH 15-1-2

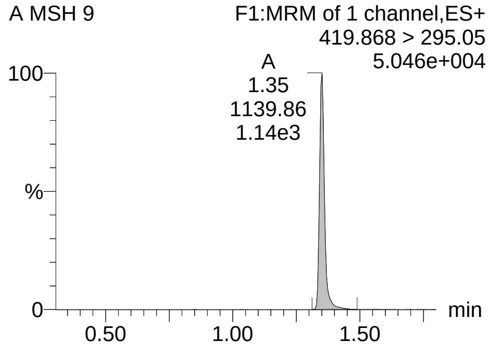

Sample Name: A MSH 15-1-3

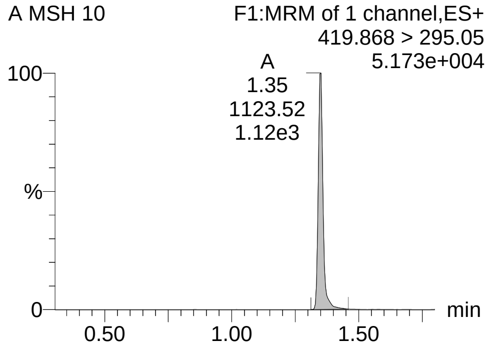

Sample Name: A MSH 15-2-1

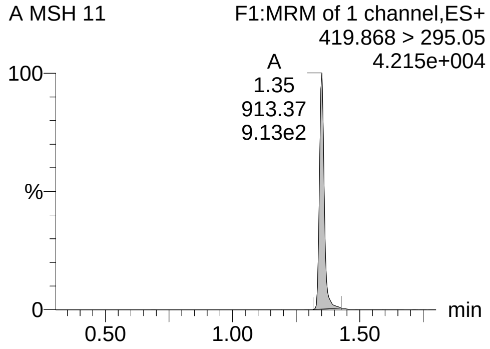

Sample Name: A MSH 15-2-2

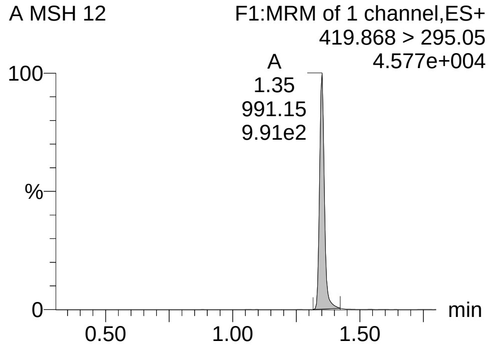

Sample Name: A MSH 15-2-3
A MSH 13 F1:MRM of 1 channel,ES+
419.868 > 295.05
4.248e+004

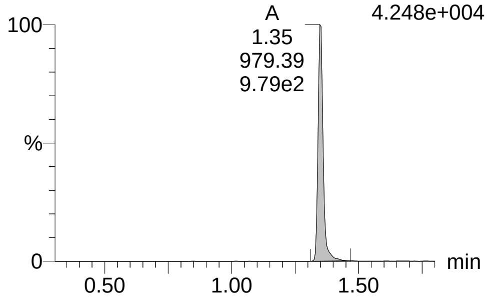

Sample Name: A MSH Blank 2
A MSH 14 F1:MRM of 1 channel,ES+
419.868 > 295.05
2.778e+001

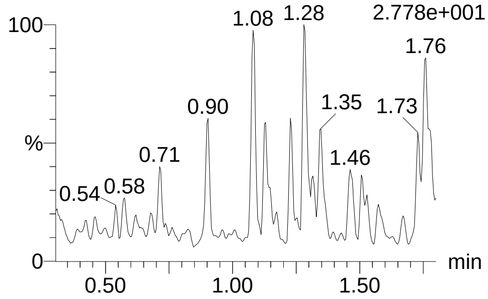

Sample Name: A MSH 30-1-1
A MSH 15 F1:MRM of 1 channel,ES+
419.868 > 295.05
4.408e+004

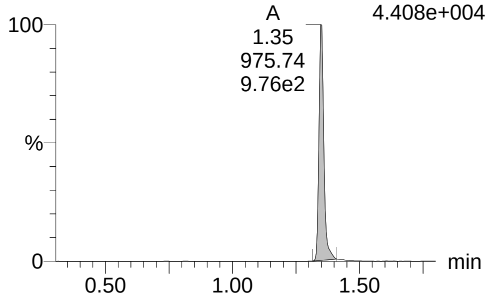

Sample Name: A MSH 30-1-2
A MSH 16 F1:MRM of 1 channel,ES+
419.868 > 295.05
4.908e+004

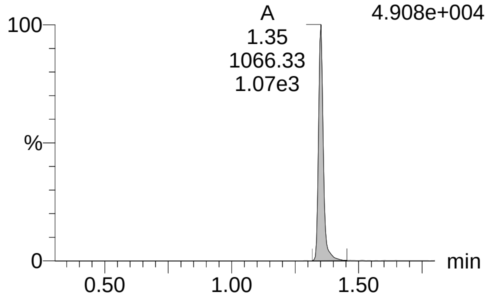

Sample Name: A MSH 30-1-3
A MSH 17 F1:MRM of 1 channel,ES+
419.868 > 295.05
5.044e+004

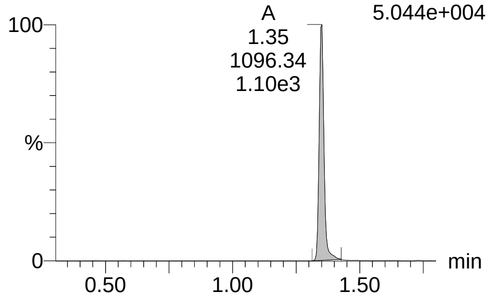

Sample Name: A MSH 30-2-1
A MSH 18 F1:MRM of 1 channel,ES+
419.868 > 295.05
4.819e+004

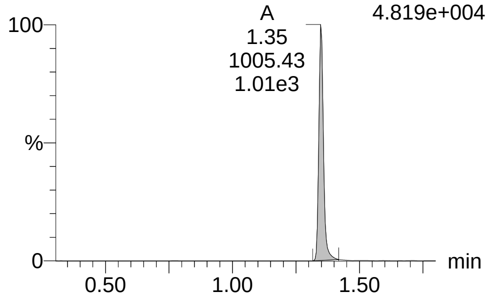

Sample Name: A MSH 30-2-2
A MSH 19 F1:MRM of 1 channel,ES+
419.868 > 295.05
4.551e+004

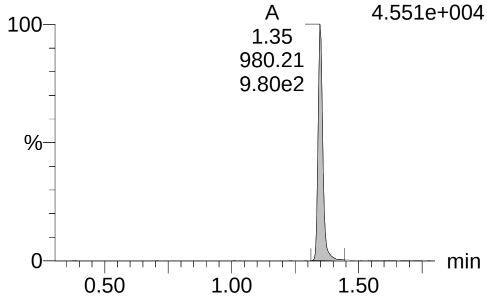

Sample Name: A MSH 30-2-3
A MSH 20 F1:MRM of 1 channel,ES+
419.868 > 295.05
4.405e+004

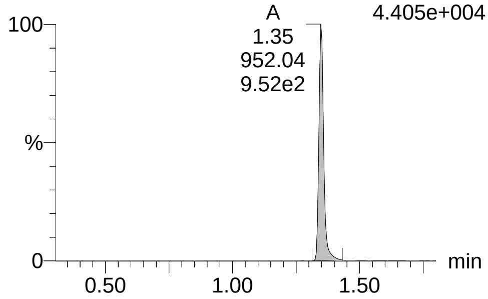

Sample Name: A MSH Blank 3
A MSH 21 F1:MRM of 1 channel,ES+
419.868 > 295.05
3.511e+001

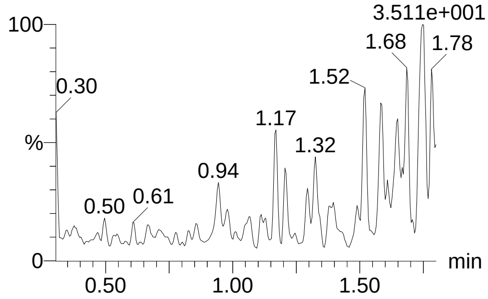

Sample Name: A MSH 1-1-1
A MSH 22 F1:MRM of 1 channel,ES+
419.868 > 295.05
4.137e+004

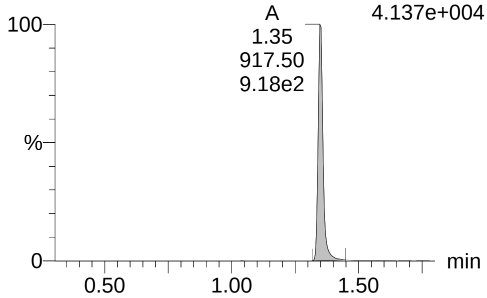

Sample Name: A MSH 1-1-2
A MSH 23 F1:MRM of 1 channel,ES+
419.868 > 295.05
3.900e+004

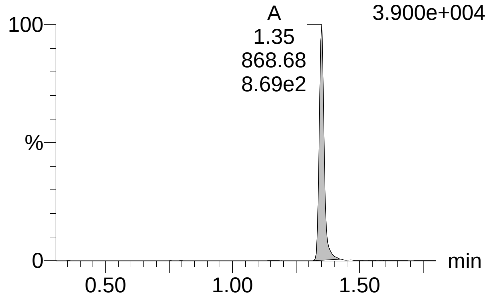

Sample Name: A MSH 1-1-3
A MSH 24 F1:MRM of 1 channel,ES+
419.868 > 295.05
4.193e+004

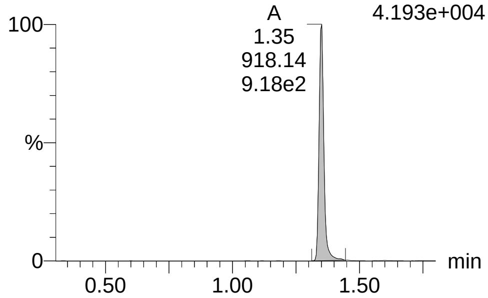

Sample Name: A MSH 1-2-1
A MSH 25 F1:MRM of 1 channel,ES+
419.868 > 295.05
3.933e+004

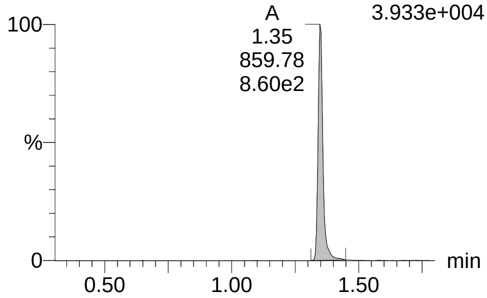

Sample Name: A MSH 1-2-2
A MSH 26 F1:MRM of 1 channel,ES+
419.868 > 295.05
3.911e+004

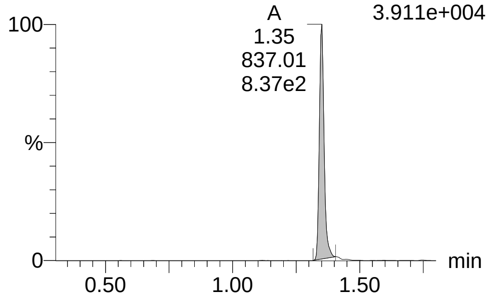

Sample Name: A MSH 1-2-3
A MSH 27 F1:MRM of 1 channel,ES+
419.868 > 295.05
4.166e+004

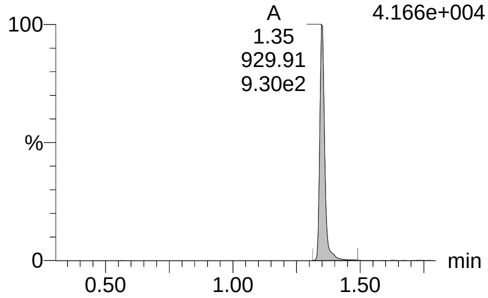

Sample Name: A MSH Blank 4
A MSH 28 F1:MRM of 1 channel,ES+
419.868 > 295.05
5.467e+001
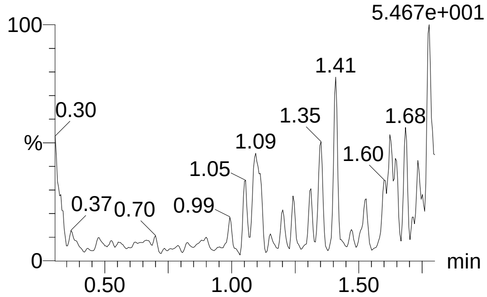

Sample Name: A MSH 2-1-1
A MSH 29 F1:MRM of 1 channel,ES+
419.868 > 295.05
4.574e+004
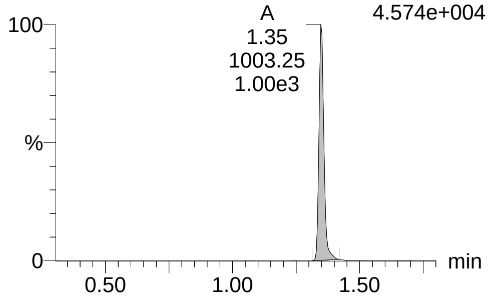

Sample Name: A MSH 2-1-2
A MSH 30 F1:MRM of 1 channel,ES+
419.868 > 295.05
4.150e+004
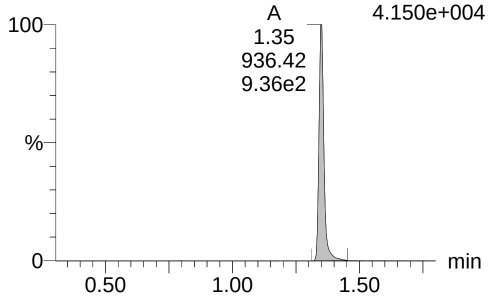

Sample Name: A MSH 2-1-3
A MSH 31 F1:MRM of 1 channel,ES+
419.868 > 295.05
4.770e+004
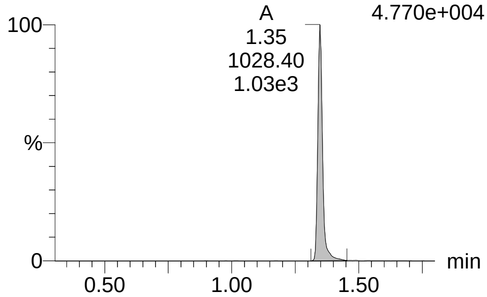

Sample Name: A MSH 2-2-1
A MSH 32 F1:MRM of 1 channel,ES+
419.868 > 295.05
3.450e+004
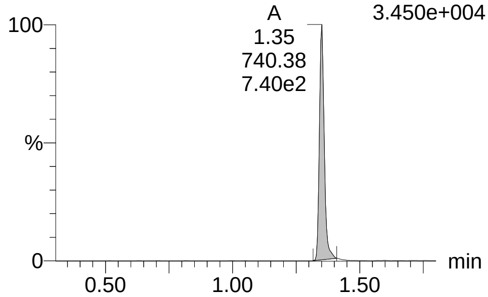

Sample Name: A MSH 2-2-2
A MSH 33 F1:MRM of 1 channel,ES+
419.868 > 295.05
3.557e+004
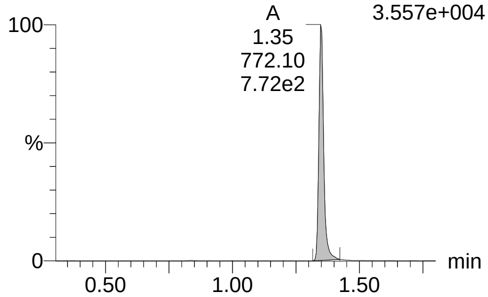

Sample Name: A MSH 2-2-3
A MSH 34 F1:MRM of 1 channel,ES+
419.868 > 295.05
3.077e+004
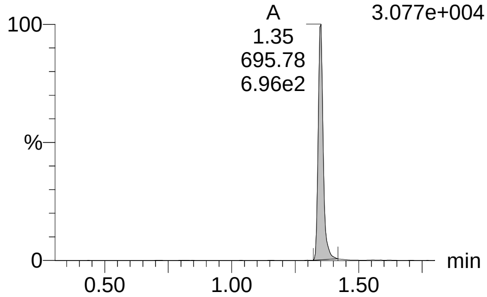

Sample Name: A MSH Blank 5
A MSH 35 F1:MRM of 1 channel,ES+
419.868 > 295.05
5.478e+001
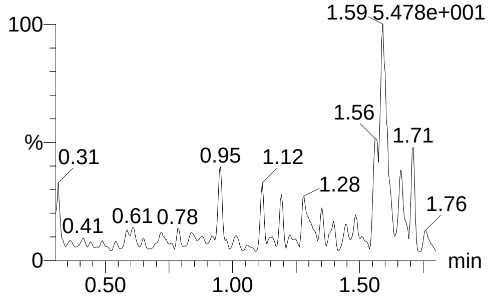

Sample Name: A MSH 4-1-1
A MSH 36 F1:MRM of 1 channel,ES+
419.868 > 295.05
4.091e+004
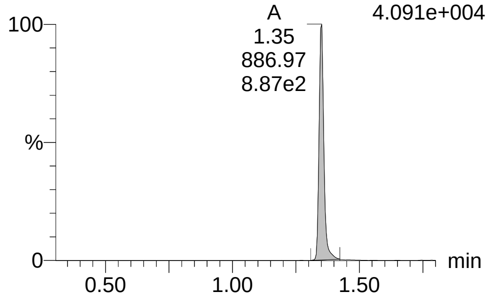

Sample Name: A MSH 4-1-2
A MSH 37 F1:MRM of 1 channel,ES+
419.868 > 295.05
4.413e+004
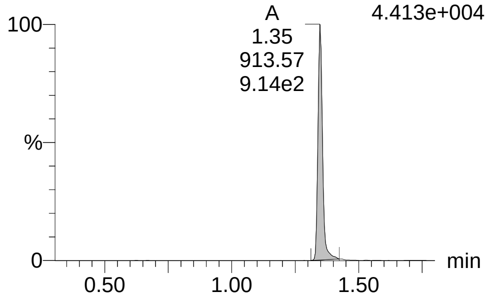

Sample Name: A MSH 4-1-3
A MSH 38 F1:MRM of 1 channel,ES+
419.868 > 295.05
3.108e+004
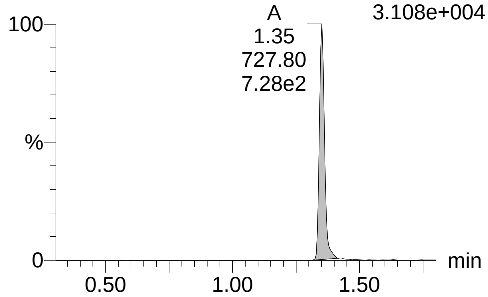

Sample Name: A MSH 4-2-1
A MSH 39 F1:MRM of 1 channel,ES+
419.868 > 295.05
2.787e+004
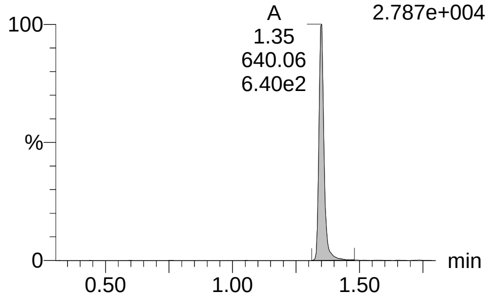

Sample Name: A MSH 4-2-2
A MSH 40 F1:MRM of 1 channel,ES+
419.868 > 295.05
2.814e+004
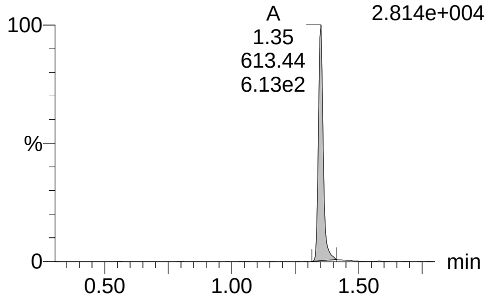

Sample Name: A MSH 4-2-3
A MSH 41 F1:MRM of 1 channel,ES+
419.868 > 295.05
3.159e+004
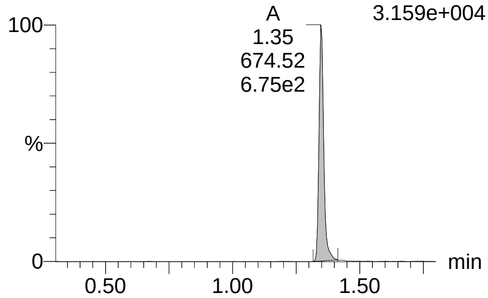

Sample Name: A MSH Blank 6
A MSH 42 F1:MRM of 1 channel,ES+
419.868 > 295.05
3.267e+001
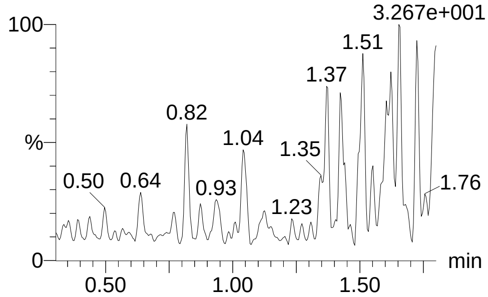

Dataset: Untitled
Last Altered: Monday, January 18, 2021 11:54:53 Romance Standard Time
Printed: Monday, January 18, 2021 11:55:44 Romance Standard Time

Sample Name: A MSH 6-1-1
A MSH 43 F1:MRM of 1 channel,ES+
419.868 > 295.05
3.202e+004

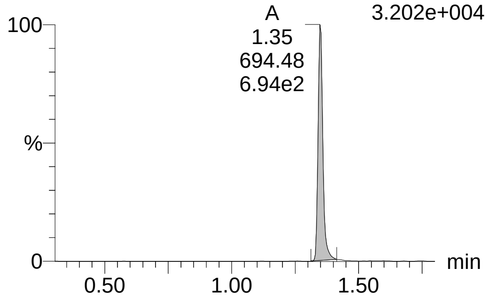

Sample Name: A MSH 6-1-2
A MSH 44 F1:MRM of 1 channel,ES+
419.868 > 295.05
3.038e+004

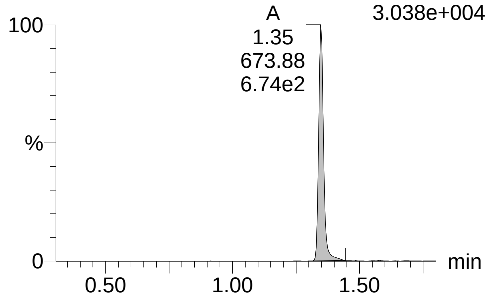

Sample Name: A MSH 6-1-3
A MSH 45 F1:MRM of 1 channel,ES+
419.868 > 295.05
3.002e+004

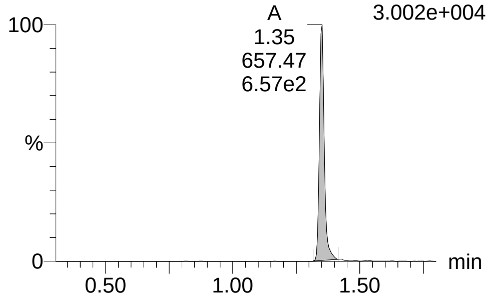

Sample Name: A MSH 6-2-1
A MSH 46 F1:MRM of 1 channel,ES+
419.868 > 295.05
2.699e+004

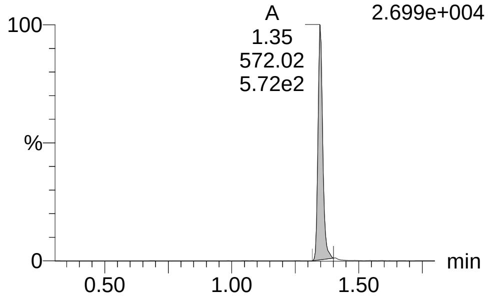

Sample Name: A MSH 6-2-2
A MSH 47 F1:MRM of 1 channel,ES+
419.868 > 295.05
2.342e+004

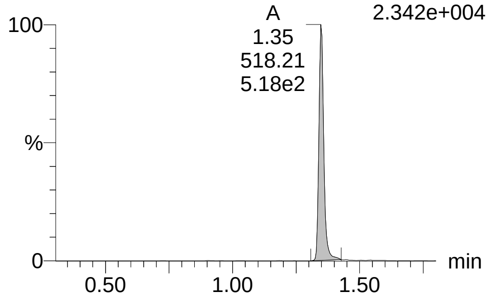

Sample Name: A MSH 6-2-3
A MSH 48 F1:MRM of 1 channel,ES+
419.868 > 295.05
2.672e+004

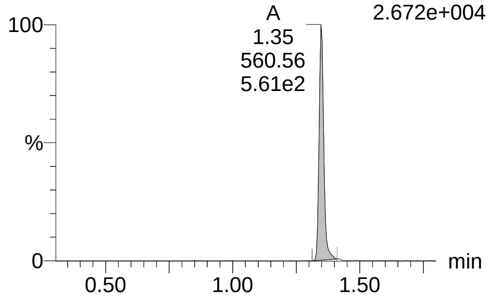

Sample Name: A MSH Blank 7
A MSH 49 F1:MRM of 1 channel,ES+
419.868 > 295.05
3.722e+001

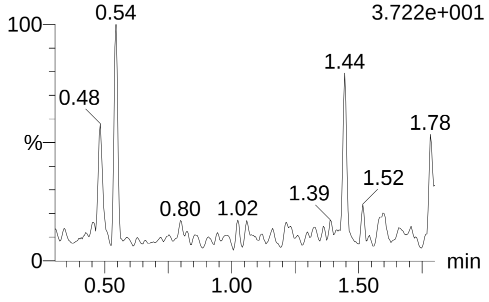

Sample Name: A MSH 24-1-1
A MSH 70 F1:MRM of 1 channel,ES+
419.868 > 295.05
2.444e+004

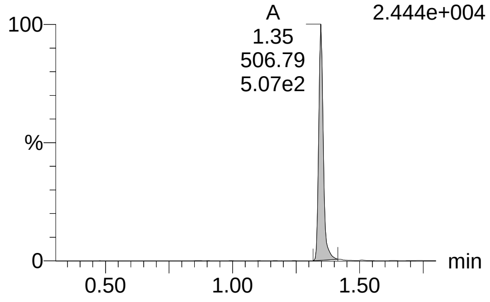

Sample Name: A MSH 24-1-2
A MSH 71 F1:MRM of 1 channel,ES+
419.868 > 295.05
2.615e+004

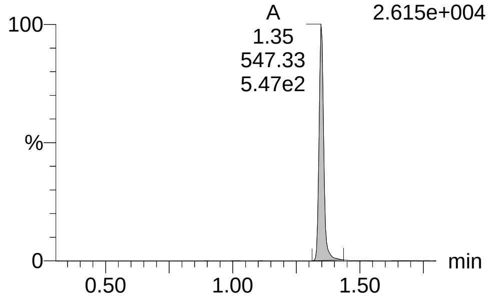

Sample Name: A MSH 24-1-3
A MSH 72 F1:MRM of 1 channel,ES+
419.868 > 295.05
2.377e+004

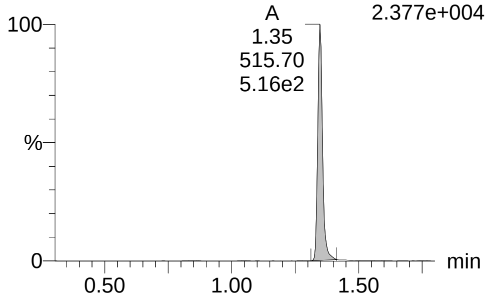

Sample Name: A MSH 24-2-1
A MSH 73 F1:MRM of 1 channel,ES+
419.868 > 295.05
2.303e+004

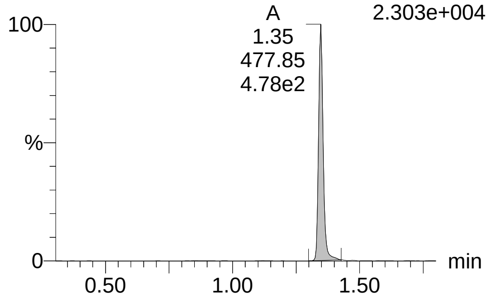

Sample Name: A MSH 24-2-2
A MSH 74 F1:MRM of 1 channel,ES+
419.868 > 295.05
2.104e+004

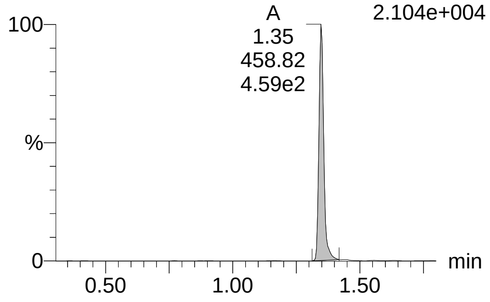

Sample Name: A MSH 24-2-3
A MSH 75 F1:MRM of 1 channel,ES+
419.868 > 295.05
2.382e+004

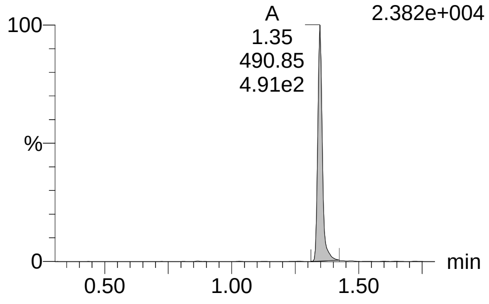

	#	Name	Type	Std. Conc	RT	Area	IS Area	Response	Detecti...	ng/mL	%Dev
1	1	A MSH 0-1-1	Anal...		1.35	1188.518		1188.518	bb		
2	2	A MSH 0-1-2	Anal...		1.35	1213.861		1213.861	bb		
3	3	A MSH 0-1-3	Anal...		1.35	1192.295		1192.295	bb		
4	4	A MSH 0-2-1	Anal...								
5	5	A MSH 0-2-2	Anal...								
6	6	A MSH 0-2-3	Anal...								
7	7	A MSH Blank 1	Blank								
8	8	A MSH 15-1-1	Anal...		1.35	1112.486		1112.486	bb		
9	9	A MSH 15-1-2	Anal...		1.35	1139.862		1139.862	bb		
10	10	A MSH 15-1-3	Anal...		1.35	1123.516		1123.516	bb		
11	11	A MSH 15-2-1	Anal...		1.35	913.365		913.365	bb		
12	12	A MSH 15-2-2	Anal...		1.35	991.147		991.147	bb		
13	13	A MSH 15-2-3	Anal...		1.35	979.387		979.387	bb		
14	14	A MSH Blank 2	Blank								
15	15	A MSH 30-1-1	Anal...		1.35	975.743		975.743	bb		
16	16	A MSH 30-1-2	Anal...		1.35	1066.325		1066.325	bb		
17	17	A MSH 30-1-3	Anal...		1.35	1096.340		1096.340	bb		
18	18	A MSH 30-2-1	Anal...		1.35	1005.427		1005.427	bb		
19	19	A MSH 30-2-2	Anal...		1.35	980.210		980.210	bb		
20	20	A MSH 30-2-3	Anal...		1.35	952.038		952.038	bb		
21	21	A MSH Blank 3	Blank								
22	22	A MSH 1-1-1	Anal...		1.35	917.503		917.503	bb		
23	23	A MSH 1-1-2	Anal...		1.35	868.685		868.685	bb		
24	24	A MSH 1-1-3	Anal...		1.35	918.138		918.138	bb		
25	25	A MSH 1-2-1	Anal...		1.35	859.782		859.782	bb		
26	26	A MSH 1-2-2	Anal...		1.35	837.005		837.005	bb		
27	27	A MSH 1-2-3	Anal...		1.35	929.915		929.915	bb		
28	28	A MSH Blank 4	Blank								
29	29	A MSH 2-1-1	Anal...		1.35	1003.246		1003.246	bb		
30	30	A MSH 2-1-2	Anal...		1.35	936.425		936.425	bb		
31	31	A MSH 2-1-3	Anal...		1.35	1028.403		1028.403	bb		
32	32	A MSH 2-2-1	Anal...		1.35	740.377		740.377	bb		
33	33	A MSH 2-2-2	Anal...		1.35	772.104		772.104	bb		
34	34	A MSH 2-2-3	Anal...		1.35	695.778		695.778	bb		
35	35	A MSH Blank 5	Blank								
36	36	A MSH 4-1-1	Anal...		1.35	886.971		886.971	bb		
37	37	A MSH 4-1-2	Anal...		1.35	913.571		913.571	bb		
38	38	A MSH 4-1-3	Anal...		1.35	727.796		727.796	bb		
39	39	A MSH 4-2-1	Anal...		1.35	640.058		640.058	bb		
40	40	A MSH 4-2-2	Anal...		1.35	613.442		613.442	bb		
41	41	A MSH 4-2-3	Anal...		1.35	674.522		674.522	bb		
42	42	A MSH Blank 6	Blank								
43	43	A MSH 6-1-1	Anal...		1.35	694.484		694.484	bb		
44	44	A MSH 6-1-2	Anal...		1.35	673.878		673.878	bb		
45	45	A MSH 6-1-3	Anal...		1.35	657.468		657.468	bb		
46	46	A MSH 6-2-1	Anal...		1.35	572.024		572.024	bb		
47	47	A MSH 6-2-2	Anal...		1.35	518.209		518.209	bb		
48	48	A MSH 6-2-3	Anal...		1.35	560.557		560.557	bb		
49	49	A MSH Blank 7	Blank								
50	50	A MSH 24-1-1	Anal...		1.35	506.786		506.786	bb		
51	51	A MSH 24-1-2	Anal...		1.35	547.331		547.331	bb		
52	52	A MSH 24-1-3	Anal...		1.35	515.698		515.698	bb		
53	53	A MSH 24-2-1	Anal...		1.35	477.848		477.848	bb		
54	54	A MSH 24-2-2	Anal...		1.35	458.824		458.824	bb		
55	55	A MSH 24-2-3	Anal...		1.35	490.852		490.852	bb		

Method: C:\MassLynx_Projects\Medchem SU.PRO\MethDB\A.mdb 11 Jan 2021 10:59:51
Calibration: 18 Jan 2021 11:54:53

```
Header
Acquired File Name:   A MSH 0-1-1
Acquired Date:        12-Jan-2021
Acquired Time:        20:53:33
Job Code:             MSH 20210112 - 002 - 026 - 75 - A
Task Code:
User Name:
Laboratory Name:
Instrument:           ACQ-TQD#QBA320
Conditions:
Submitter:
SampleID:             A MSH 1
Bottle Number:        2:64
Description:
Instrument Calibration:
Calibration File: C:\MassLynx\IntelliStart\Results\Unit Mass Resolution\Calib 20161209.cal
Parameters
MS1 Static:
Mass:             20 Da to 1974 Da.
Resolution:       15.0/15.0
Ion Energy:       0.5
Reference File:   Naics2
Acquisition File: STATMS1
MS1 Scanning:
Mass:             15 Da to 2048 Da.
Resolution:       15.0/15.0
Ion Energy:       0.5
Reference File:   Naics2
Acquisition File: SCNMS1
MS1 Scan Speed Compensation:
Scan:             339 to 2000 amu/sec.
Resolution:       15.0/15.0
Ion Energy:       0.5
Reference File:   Naics2
Acquisition File: FASTMS1
MS2 Static:
Mass:             20 Da to 1974 Da.
Resolution:       15.0/15.0
Ion Energy:       0.5
Reference File:   Naics2
Acquisition File: STATMS2
MS2 Scanning:
Mass:             15 Da to 2048 Da.
Resolution:       15.0/15.0
Ion Energy:       0.5
Reference File:   Naics2
Acquisition File: SCNMS2
MS2 Scan Speed Compensation:
Scan:             339 to 10165 amu/sec.
Resolution:       15.0/15.0
Ion Energy:       0.5
Reference File:   Naics2
Acquisition File: FASTMS2
Calibration Time: 10:33
Calibration Date: 12/09/16
Coefficients
MS1 Static:       -0.000000000000*x^4 + 0.000000000843*x^3 + -0.000001360559*x^2 + 1.000940122634*x
 +-0.253266439024
MS2 Static:       -0.000000000000*x^4 + 0.000000000573*x^3 + -0.000001239648*x^2 + 1.000902600483*x
 +-0.192715155436
Function 1:       None
Function 2:       None
Parameters for C:\Documents and Settings\Administrator\Desktop\old desktop\log D\EA.EXP
Data Processing:
SIR / MRM Chromatogram Spike Removal      ON
```

```
SIR / MRM Smoothing           OFF
Smoothing window size (scans) 3
Number of smooths       2
Prescan Statistics:
Initial Average Intensity     22.0947
Initial Average Std Dev       1.7772
Bunch Zero Level        0.0032
Bunch Std Dev                 0.0089
Bunch Threshold               0.0716
Spike Removal Std Dev         1.7691
Method Events:
Initial Stop Flow:      No Change
Initial Switch 2: No Change
Initial Switch 3: No Change
Initial Switch 4: No Change
Initial Infusion: No Change
Initial Flow State:     LC
Initial Flow Rate:      5
Initial Reservoir:      No Action
API Probe Delay Temp:   20
Initial Refill:         No Action
Timed Events Enabled
Event Time  Name        Action
Instrument Parameters - Function 1:
Parameter File - C:\Documents and Settings\Administrator\Desktop\old desktop\log D\log D tune file.
IPR
Polarity    ES+
Calibration Static 2
Capillary (kV)    3.50  3.48
Cone (V)    38.00 33.46
Extractor (V)     3.00  2.20
RF (V)      0.10
Source Temperature (°C) 140   138
Desolvation Temperature (°C)  450   450
Cone Gas Flow (L/Hr)    90    90
Desolvation Gas Flow (L/Hr)   900   900
Collision Gas Flow (mL/Min)   0.20  0.20
LM 1 Resolution   15.50
HM 1 Resolution   14.84
Ion Energy 1      0.30
MS Mode Entrance  50.00
MS Mode Collision Energy      3.00
MS Mode Exit      50.00
MSMS Mode Entrance      -2.00
MSMS Mode Collision Energy    2.00
MSMS Mode Exit    2.00
LM 2 Resolution   15.00
HM 2 Resolution   15.50
Ion Energy 2      1.22
Gain  1.00
Multiplier  -495.12
Active Reservoir  B
Engineers Settings:
MS1 Low Mass Position         518
MS1 High Mass Position        284
MS1 Low Mass Resolution       513
MS1 High Mass Resolution      1732
MS1 Resolution Linearity      834
MS1 High Mass DC Balance      0
MS1 DC Polarity               Positive
MS2 Low Mass Position         519
MS2 High Mass Position        238
MS2 Low Mass Resolution       518
MS2 High Mass Resolution      644
MS2 Resolution Linearity      756
MS2 High Mass DC Balance      -0
MS2 DC Polarity               Positive
HM RF Lens Correction +       0
```

```
HM RF Lens Correction -       0
Inter-scan delays:
Automatic Mode
MS 1 Delay Table:
         R     delay
<=   0.500     0.005
<=   1.200     0.010
<=   2.400     0.015
<=   6.000     0.020
<=  15.000     0.025
<=  25.000     0.028
>   25.000     0.030
MS 2 Delay Table:
         R     delay
<=   2.000     0.005
<=   4.000     0.008
<=   7.000     0.010
<=  10.000     0.012
<=  20.000     0.014
>   20.000     0.016
ACE Experimental Record
Inlet Method File: c:\masslynx_projects\medchem su.pro\acqudb\adme sophie
-------------------   Prerun method parameters   ---------------
Waters ACQUITY QSM
Waters Acquity TUV
 Run Time: 0.20 min
 Wavelength Mode: Single Wavelength
 Lamp On: On
Channel A...
 Comment:
 Wavelength: 254 nm
 Sampling Rate: 20 points/sec
 Data Mode: Absorbance
 Time Constant: 0.1000 sec
 Auto Zero On Wavelength Change: Maintain Baseline
 Auto Zero On Inject Start: Yes
Analog 1...
 Sensitivity: 2.000 AUFS
 Chart Polarity: Positive (+)
 Voltage Offset: 0 mV
 Enable Chart Mark: Yes
 Run Events: Yes
 Pulse Width: 1.0 sec
 Rect Wave Period: 0.2 sec
----------------------------  oOo  ---------------------------
---------------------   Run method parameters   ----------------
Waters ACQUITY QSM
 Solvent A Name: Water
 Solvent B Name: Acetonitrile
 Solvent C Name: water 1 % FA
 Solvent D Name:
 Low Pressure Limit: 0 psi
 High Pressure Limit: 15000 psi
 Seal Wash Period: 5.00 min
 [Gradient Table]
  Time(min) Flow Rate(mL/min) %A %B %C %D Curve
 1. Initial 0.700 90.0 5.0 5.0 0.0 Initial
 2. 0.15 0.700 45.0 50.0 5.0 0.0 6
 3. 1.50 0.700 0.0 95.0 5.0 0.0 6
 4. 1.80 0.700 90.0 5.0 5.0 0.0 1
 Comment: ACQUITY UPLC BEH C18 2.1 x 50 mm
 Flow Ramp Rate: 0.45 min
 D Solvent Selection (if supported): No Change
 System Pressure Data Channel: No
 Flow Rate Data Channel: No
 %A Data Channel: No
 %B Data Channel: No
 %C Data Channel: No
```

```
 %D Data Channel: No
 Primary Data Channel: No
 Accumulator Data Channel: No
 Degasser Data Channel: No
 Gradient Start: At Injection
 Gradient Start Volume: 0 uL
 Gradient Start Time: 0.00 min
 Participate in pre-analysis: No
Waters Acquity TUV
 Run Time: 1.80 min
 Wavelength Mode: Single Wavelength
 Lamp On: On
Channel A...
 Comment:
 Wavelength: 214 nm
 Sampling Rate: 20 points/sec
 Data Mode: Absorbance
 Time Constant: 0.1000 sec
 Auto Zero On Wavelength Change: Maintain Baseline
 Auto Zero On Inject Start: Yes
Analog 1...
 Sensitivity: 2.000 AUFS
 Chart Polarity: Positive (+)
 Voltage Offset: 0 mV
 Enable Chart Mark: Yes
 Run Events: Yes
 Pulse Width: 1.0 sec
 Rect Wave Period: 0.2 sec
Waters ACQUITY FTN AutoSampler
 Run Time: 1.80 min
 Comment: ACQUITY UPLC BEH C18 2.1 x 50 mm
 Load Ahead: Disabled
 Loop Offline: Automatic min
 Wash Solvent Name: Acetonitrile
 Pre-Inject Wash Time: 0.0 sec
 Post-Inject Wash Time: 6.0 sec
 Purge Solvent Name: Water
 Dilution: Disabled
 Dilution Volume: 0 uL
 Delay Time: 0 min
 Dilution Needle Placement: 4.0 mm
 Target Column Temperature: 40.0 C
 Column Temperature Alarm Band: Disabled
 Target Sample Temperature: 15.0 C
 Sample Temperature Alarm Band: Disabled
 Syringe Draw Rate: Automatic
 Needle Placement: Automatic
 Pre-Aspirate Air Gap: Automatic
 Post-Aspirate Air Gap: Automatic
 Column Temperature Data Channel: No
 Room Temperature Data Channel: No
 Sample Temperature Data Channel: No
 Sample Organizer Temperature Data Channel: No
 Sample Pressure Data Channel: No
 Preheater Temperature Data Channel: No
 Seal Force Data Channel: No
 No Injection Mode Enabled: No
 Run Events: No
Sample Run Injection Parameter
Injection Volume (ul)   -   3.00
----------------------------  oOo  -----------------------------
End of experimental record.
-------------------- Waters ACQUITY QSM Postrun Report  ---------------
 Firmware Version: 1.50.237 (May 18 2011)
 Software Version: 1.50.1621
 Checksum: 0xae400516
 Serial Number: M09QSM056N
 Minimum System Pressure: 0.0 psi
```

```
 Maximum System Pressure: 0.0 psi
 Average System Pressure: 0.0 psi
----------------------------  oOo  ----------------------------
------------------- Waters ACQUITY FTN Postrun Report  ---------------
 Software Version: 1.50.1481
 Firmware Version: 1.50.317 (Jul 11 2011)
 Checksum: 0x3e83519d
 Serial Number: M09SDI055N
 Sample Syringe Size: 100.0
 Extension Loop Size: 0.0
 Needle Size: 15.0
 Column Type: ACQUITY UPLC® BEH C18 1.7µm
 Column Serial Number: 02343407715781
 Total Injections on Column: 1145
 Minimum Sample Temperature: 0.0
 Maximum Sample Temperature: 0.0
 Average Sample Temperature: 0.0
 Minimum Column Temperature: 30.0
 Maximum Column Temperature: 41.4
 Average Column Temperature: 0.0
----------------------------  oOo  ----------------------------
------------------- Generic Instrument Postrun Report  ---------------
 Software Version: 1.50.2530
 Firmware Version: 1.50.2182 (May 11 2011)
 Checksum: 0xc09b9cb2
 Serial Number: J08UPT460M
 Lamp On/Off Event: No
 Lamp Life: 100.00
 Lamp Serial Number: 000296721
 Flow Cell Type: Other
 Flow Cell Path Length: 0.00 mm
 Flow Cell Volume: 0.00 microliters
 Flow Cell Serial Number: 1
 Flow Cell Part Number: 1
 Optics Temperature Stabilzation Setting: unknown
----------------------------  oOo  ----------------------------
------------------- Waters ACQUITY QSM Postrun Report  ---------------
 Firmware Version: 1.50.237 (May 18 2011)
 Software Version: 1.50.1621
 Checksum: 0xae400516
 Serial Number: M09QSM056N
 Minimum System Pressure: 0.0 psi
 Maximum System Pressure: 0.0 psi
 Average System Pressure: 0.0 psi
----------------------------  oOo  ----------------------------
Function 1
Scans in function:          338
Cycle time (secs):          Automatic
Inter Scan Delay (secs):    Automatic
Inter Channel Delay (secs):Automatic
Span (Da):                  2.000
Start and End Time(mins):    0.300 to 1.800
Ionization mode:            ES+
Data type:                  SIR or MRM data
Function type:              MRM of 1 channel
Chan Reaction               Dwell(secs) Cone Volt. Col.Energy Delay(secs) Compound Formula|Mass Comm
ents
1  : 419.87 > 295.05           0.260       30.0       18.0       Auto     EA       418.9        Inte
lliStart Generated
Function 2
Scans in function:          2161
Function type:              Diode Array
Wavelength range (nm):       214 to 214
```

Dataset: Untitled

Last Altered: Wednesday, August 18, 2021 15:58:43 Romance Daylight Time

Printed: Wednesday, August 18, 2021 15:59:15 Romance Daylight Time

Method: C:\MassLynx_Projects\Medchem SU.PRO\MethDB\A.mdb 11 Jan 2021 11:59:51
Calibration: 18 Aug 2021 15:58:43

Compound name: A
No Calibration
Response type: External Std, Area
Curve type: Linear, Origin: Exclude, Weighting: 1/x, Axis trans: None

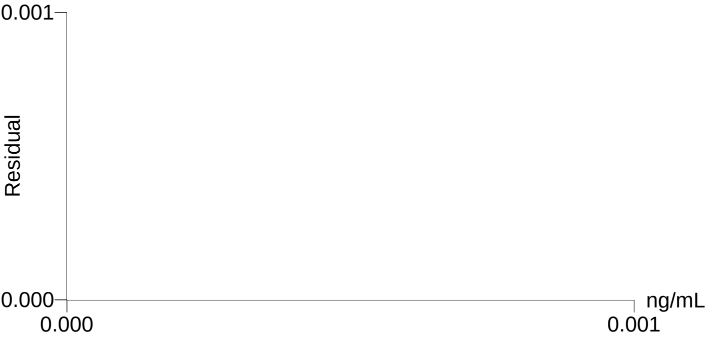

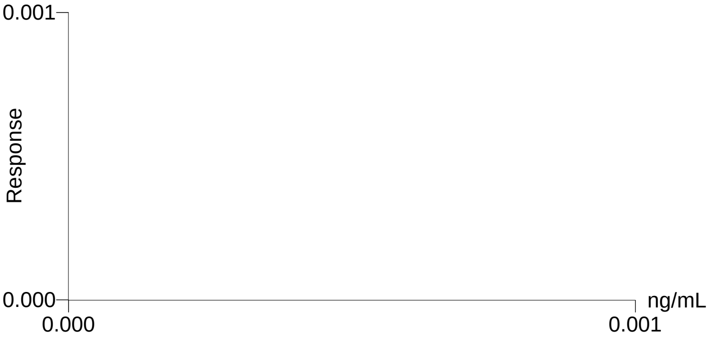

Method: C:\MassLynx_Projects\Medchem SU.PRO\MethDB\A.mdb 11 Jan 2021 11:59:51
Calibration: 18 Aug 2021 15:58:43

Sample Name: A MSH 2 0-1-1

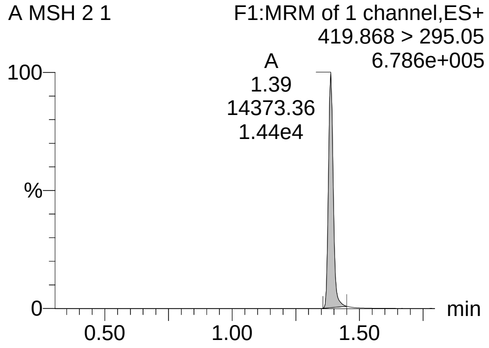

Sample Name: A MSH 2 0-1-2

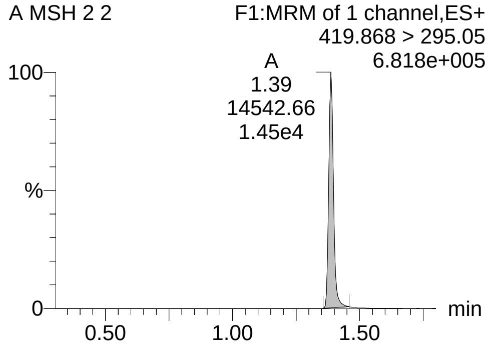

Sample Name: A MSH 2 0-1-3

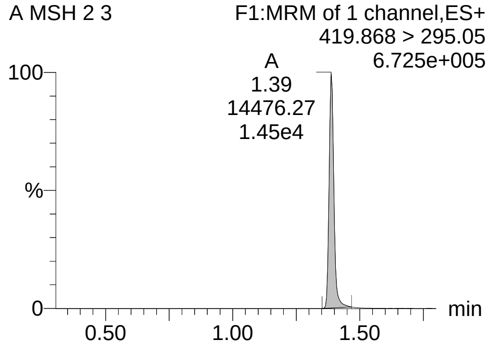

Sample Name: A MSH 2 0-2-1

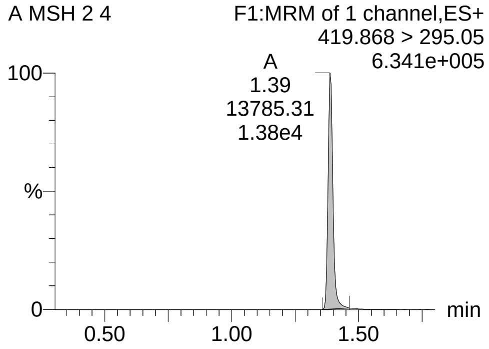

Sample Name: A MSH 2 0-2-2

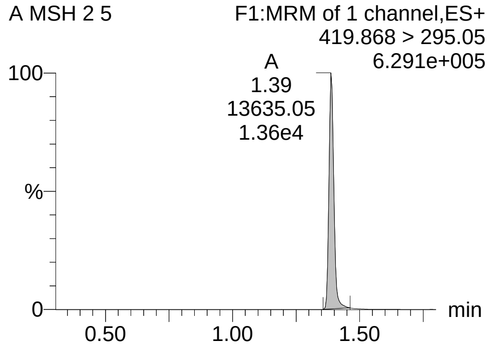

Sample Name: A MSH 2 0-2-3

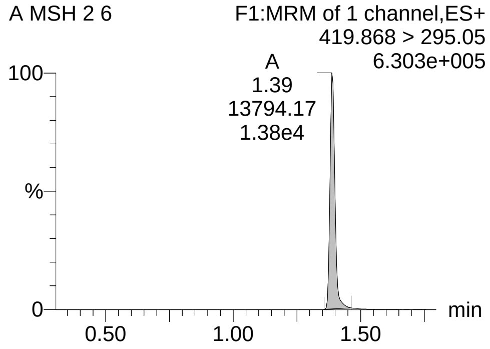

Sample Name: A MSH 2 Blank 1

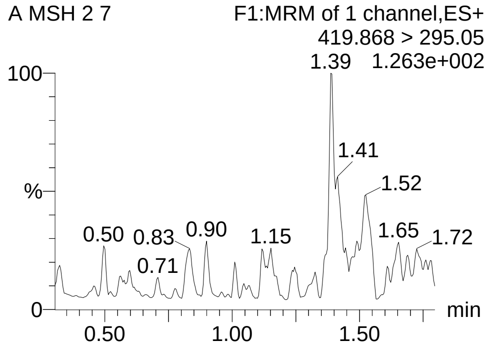

Sample Name: A MSH 2 15-1-1

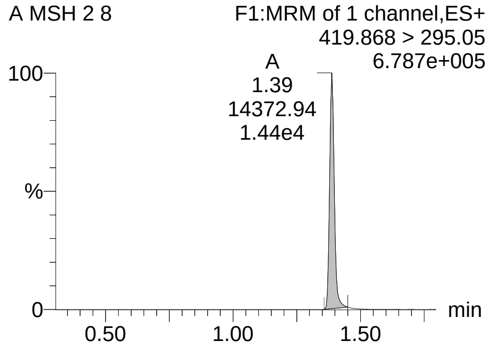

Sample Name: A MSH 2 15-1-2

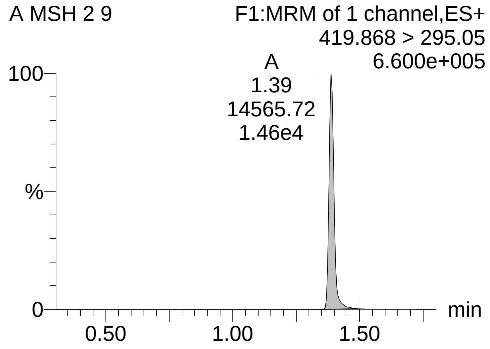

Sample Name: A MSH 2 15-1-3

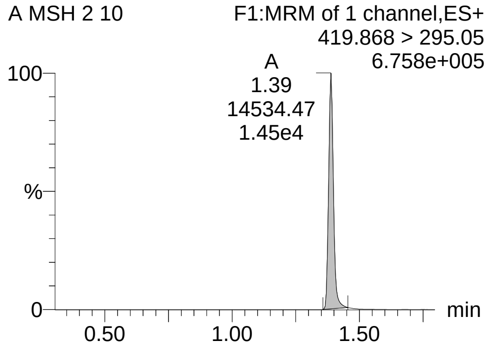

Sample Name: A MSH 2 15-2-1

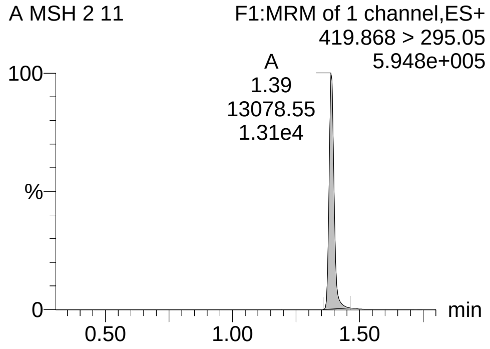

Sample Name: A MSH 2 15-2-2

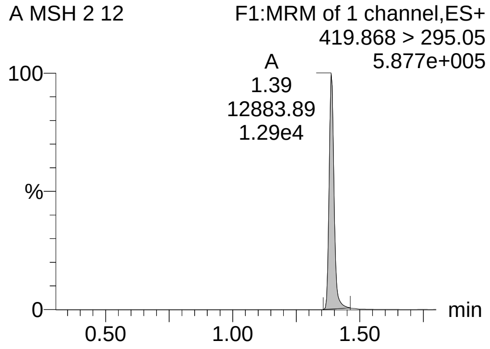

Sample Name: A MSH 2 15-2-3
A MSH 2 13 F1:MRM of 1 channel,ES+
419.868 > 295.05

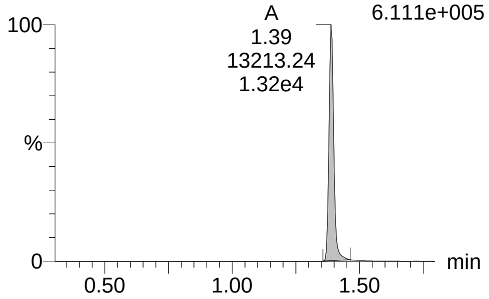

Sample Name: A MSH 2 Blank 2
A MSH 2 14 F1:MRM of 1 channel,ES+
419.868 > 295.05

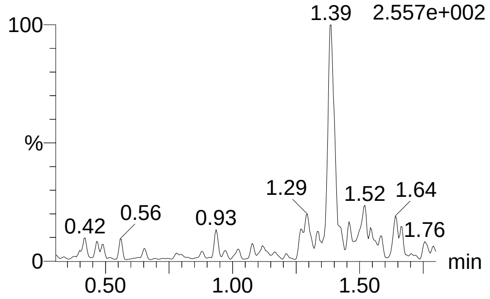

Sample Name: A MSH 2 30-1-1
A MSH 2 15 F1:MRM of 1 channel,ES+
419.868 > 295.05

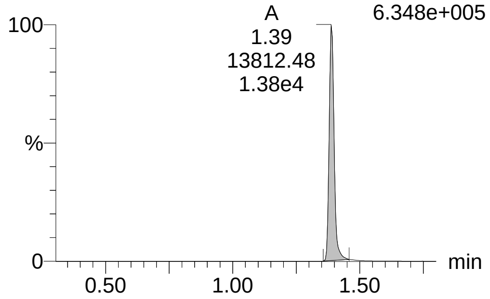

Sample Name: A MSH 2 30-1-2
A MSH 2 16 F1:MRM of 1 channel,ES+
419.868 > 295.05

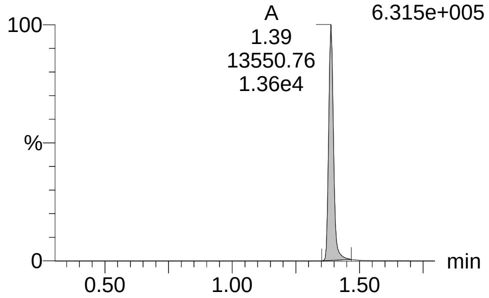

Sample Name: A MSH 2 30-1-3
A MSH 2 17 F1:MRM of 1 channel,ES+
419.868 > 295.05

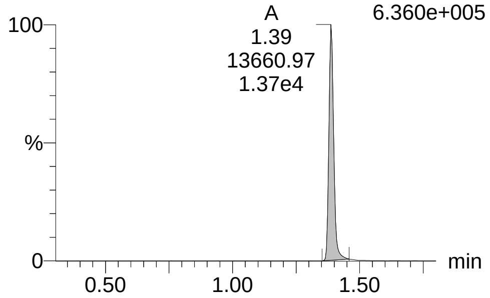

Sample Name: A MSH 2 30-2-1
A MSH 2 18 F1:MRM of 1 channel,ES+
419.868 > 295.05

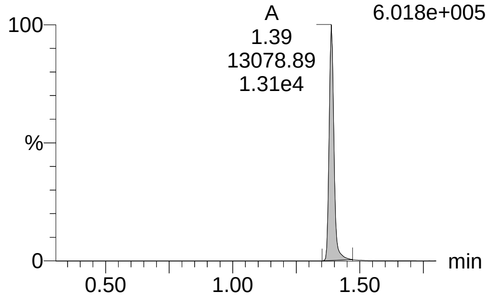

Sample Name: A MSH 2 30-2-2
A MSH 2 19 F1:MRM of 1 channel,ES+
419.868 > 295.05

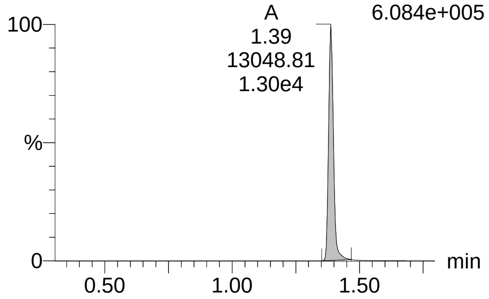

Sample Name: A MSH 2 30-2-3
A MSH 2 20 F1:MRM of 1 channel,ES+
419.868 > 295.05

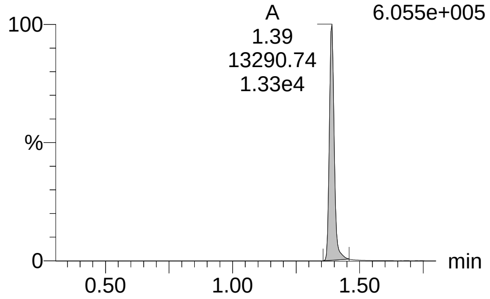

Sample Name: A MSH 2 Blank 3
A MSH 2 21 F1:MRM of 1 channel,ES+
419.868 > 295.05

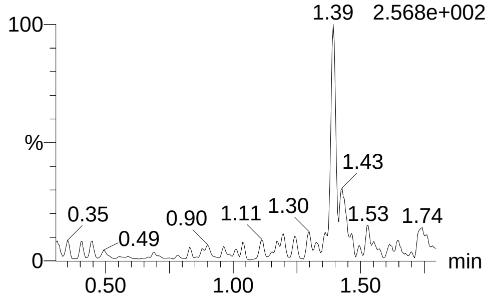

Sample Name: A MSH 2 1-1-1
A MSH 2 22 F1:MRM of 1 channel,ES+
419.868 > 295.05

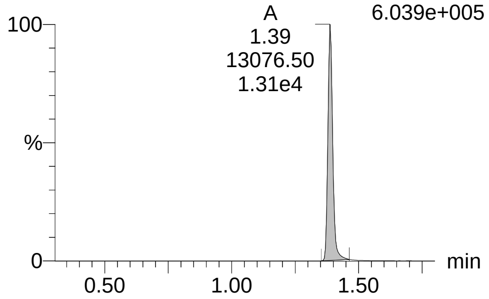

Sample Name: A MSH 2 1-1-2
A MSH 2 23 F1:MRM of 1 channel,ES+
419.868 > 295.05

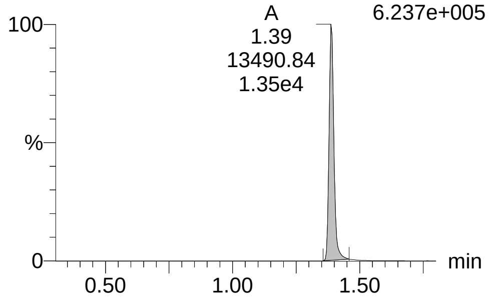

Sample Name: A MSH 2 1-1-3
A MSH 2 24 F1:MRM of 1 channel,ES+
419.868 > 295.05

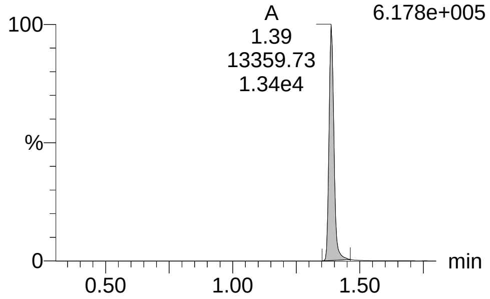

Sample Name: A MSH 2 1-2-1
A MSH 2 25 F1:MRM of 1 channel,ES+
419.868 > 295.05

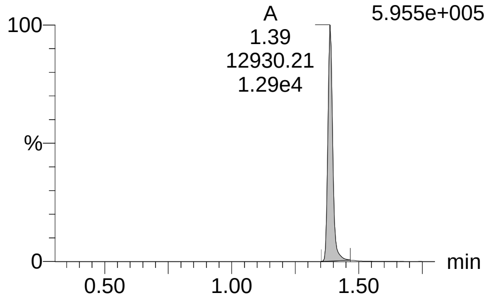

Sample Name: A MSH 2 1-2-2
A MSH 2 26 F1:MRM of 1 channel,ES+
419.868 > 295.05

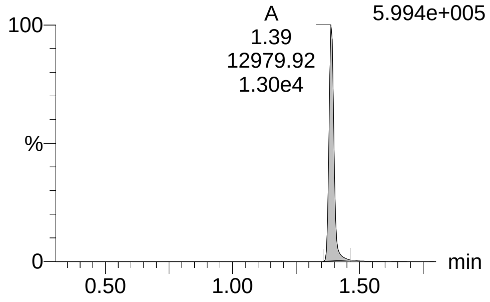

Sample Name: A MSH 2 1-2-3
A MSH 2 27 F1:MRM of 1 channel,ES+
419.868 > 295.05

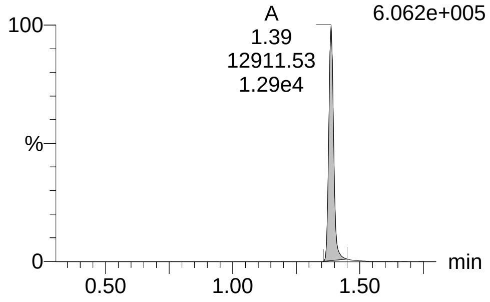

Dataset: Untitled
Last Altered: Wednesday, August 18, 2021 15:58:43 Romance Daylight Time
Printed: Wednesday, August 18, 2021 15:59:15 Romance Daylight Time

Sample Name: A MSH 2 Blank 4
A MSH 2 28 F1:MRM of 1 channel,ES+
419.868 > 295.05

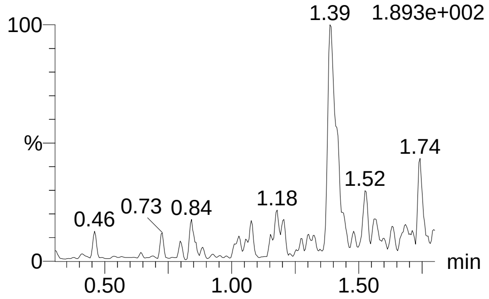

Sample Name: A MSH 2 2-1-1
A MSH 2 29 F1:MRM of 1 channel,ES+
419.868 > 295.05

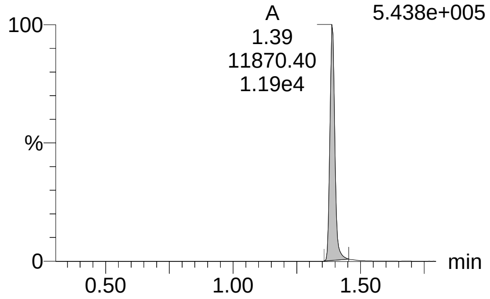

Sample Name: A MSH 2 2-1-2
A MSH 2 30 F1:MRM of 1 channel,ES+
419.868 > 295.05

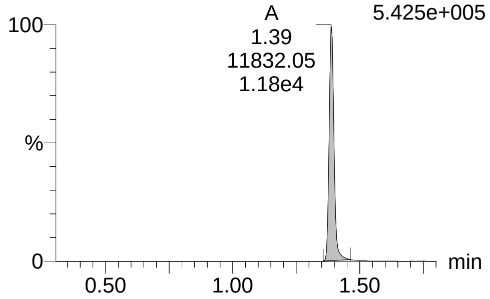

Sample Name: A MSH 2 2-1-3
A MSH 2 31 F1:MRM of 1 channel,ES+
419.868 > 295.05

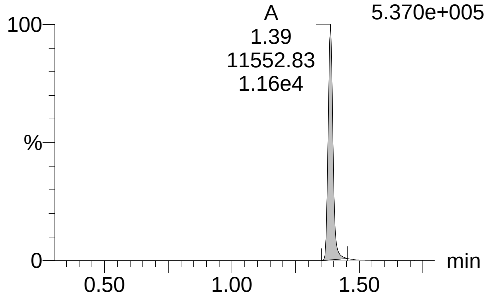

Sample Name: A MSH 2 2-2-1
A MSH 2 32 F1:MRM of 1 channel,ES+
419.868 > 295.05

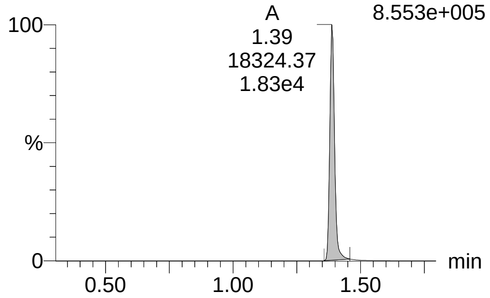

Sample Name: A MSH 2 2-2-2
A MSH 2 33 F1:MRM of 1 channel,ES+
419.868 > 295.05

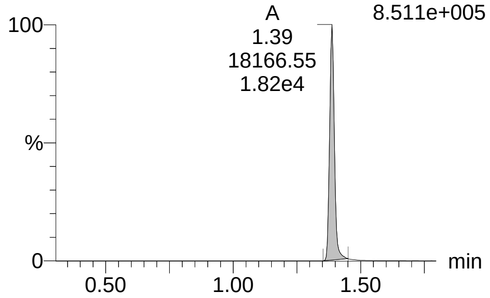

Sample Name: A MSH 2 2-2-3
A MSH 2 34 F1:MRM of 1 channel,ES+
419.868 > 295.05

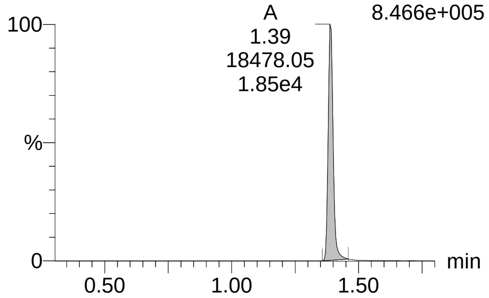

Sample Name: A MSH 2 Blank 5
A MSH 2 35 F1:MRM of 1 channel,ES+
419.868 > 295.05

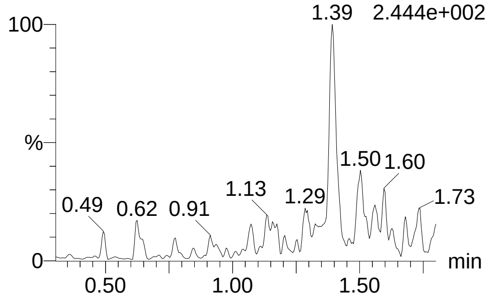

Sample Name: A MSH 2 4-1-1
A MSH 2 36 F1:MRM of 1 channel,ES+
419.868 > 295.05

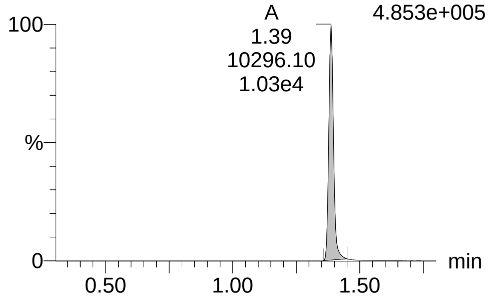

Sample Name: A MSH 2 4-1-2
A MSH 2 37 F1:MRM of 1 channel,ES+
419.868 > 295.05

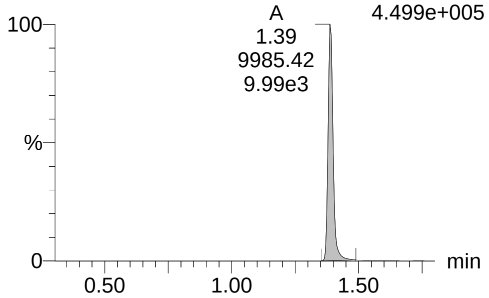

Sample Name: A MSH 2 4-1-3
A MSH 2 38 F1:MRM of 1 channel,ES+
419.868 > 295.05

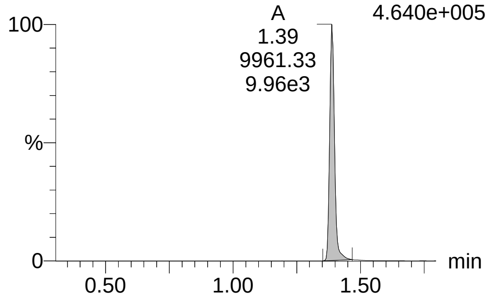

Sample Name: A MSH 2 4-2-1
A MSH 2 39 F1:MRM of 1 channel,ES+
419.868 > 295.05

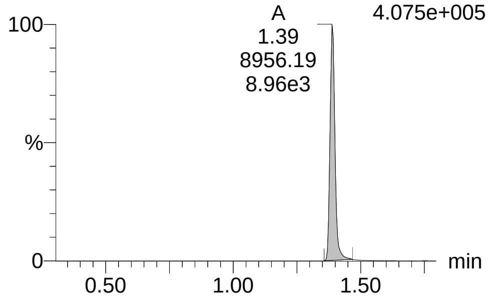

Sample Name: A MSH 2 4-2-2
A MSH 2 40 F1:MRM of 1 channel,ES+
419.868 > 295.05

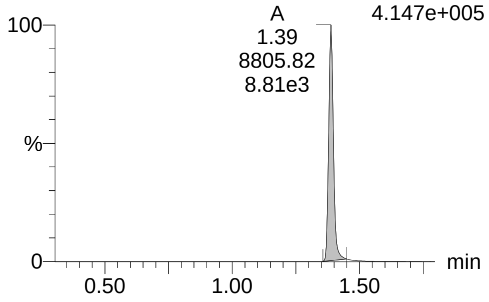

Sample Name: A MSH 2 4-2-3
A MSH 2 41 F1:MRM of 1 channel,ES+
419.868 > 295.05

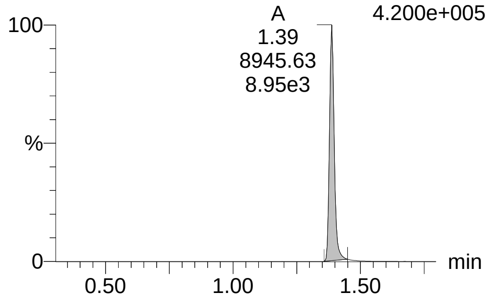

Sample Name: A MSH 2 Blank 6
A MSH 2 42 F1:MRM of 1 channel,ES+
419.868 > 295.05

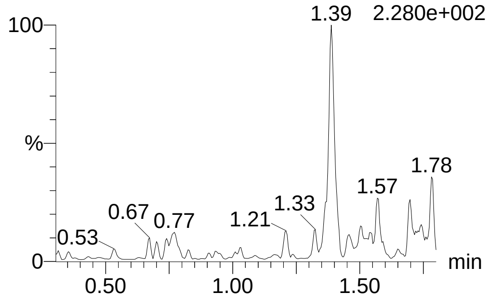

Sample Name: A MSH 2 6-1-1
A MSH 2 43 F1:MRM of 1 channel,ES+
419.868 > 295.05

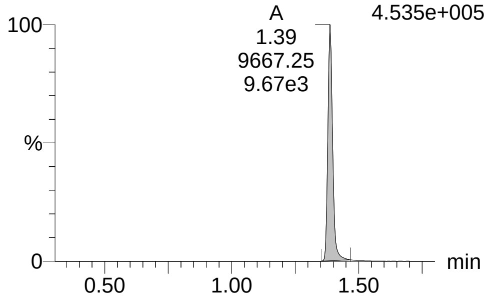

Sample Name: A MSH 2 6-1-2
A MSH 2 44 F1:MRM of 1 channel,ES+
419.868 > 295.05

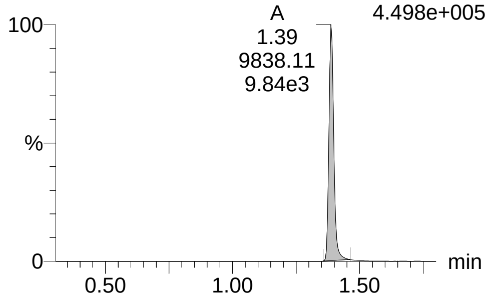

Sample Name: A MSH 2 6-1-3
A MSH 2 45 F1:MRM of 1 channel,ES+
419.868 > 295.05

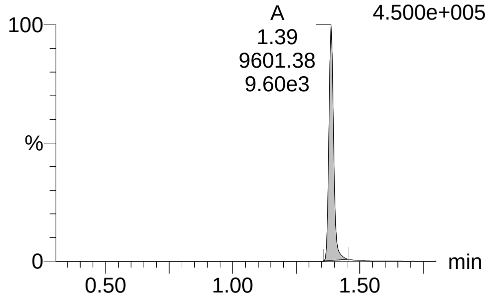

Sample Name: A MSH 2 6-2-1
A MSH 2 46 F1:MRM of 1 channel,ES+
419.868 > 295.05

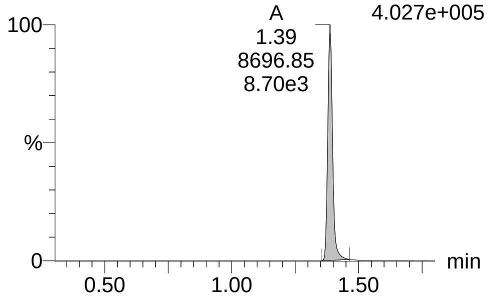

Sample Name: A MSH 2 6-2-2
A MSH 2 47 F1:MRM of 1 channel,ES+
419.868 > 295.05

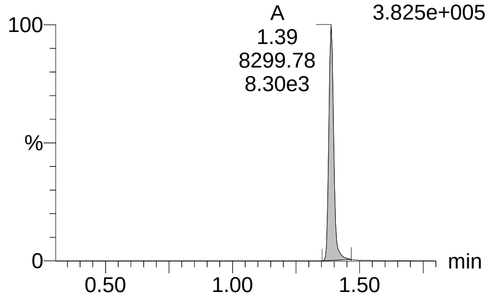

Sample Name: A MSH 2 6-2-3
A MSH 2 48 F1:MRM of 1 channel,ES+
419.868 > 295.05

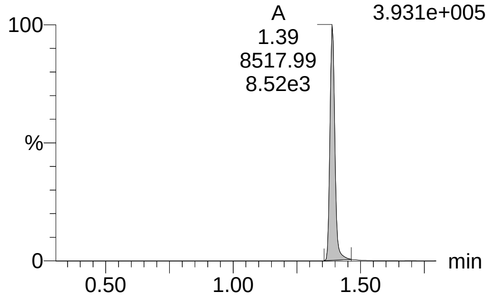

Sample Name: A MSH 2 24-1-1
A MSH 2 70 F1:MRM of 1 channel,ES+
419.868 > 295.05

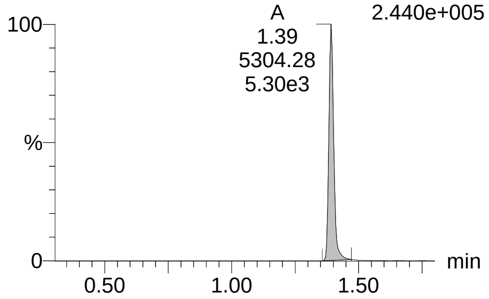

Sample Name: A MSH 2 24-1-2
A MSH 2 71 F1:MRM of 1 channel,ES+
419.868 > 295.05

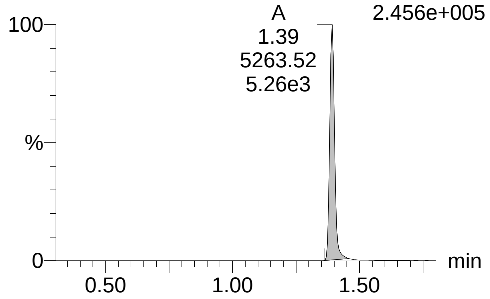

Sample Name: A MSH 2 24-1-3
A MSH 2 72 F1:MRM of 1 channel,ES+
419.868 > 295.05

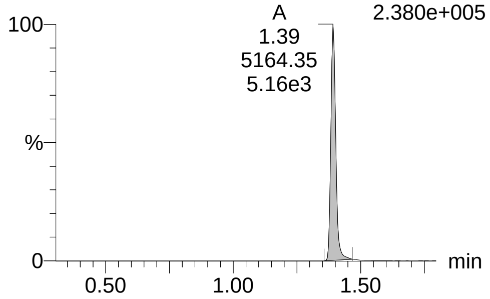

Sample Name: A MSH 2 24-2-1
A MSH 2 73 F1:MRM of 1 channel,ES+
419.868 > 295.05

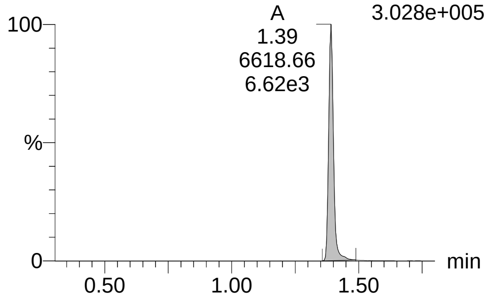

Sample Name: A MSH 2 24-2-2
A MSH 2 74 F1:MRM of 1 channel,ES+
419.868 > 295.05

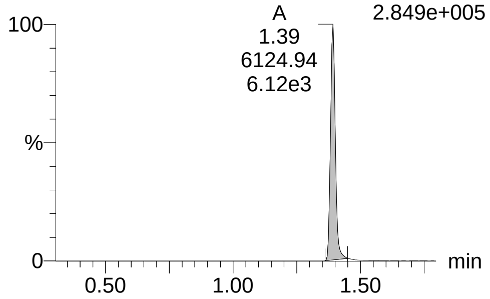

Sample Name: A MSH 2 24-2-3
A MSH 2 75 F1:MRM of 1 channel,ES+
419.868 > 295.05

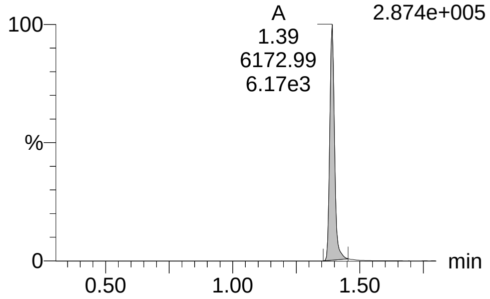

Sample Name: A MSH 2 Blank 7
A MSH 2 49 F1:MRM of 1 channel,ES+
419.868 > 295.05

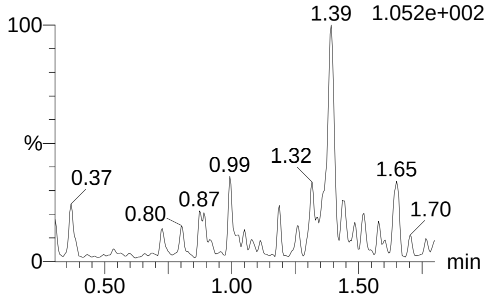

Dataset: Untitled
Last Altered: Wednesday, August 18, 2021 15:58:43 Romance Daylight Time
Printed: Wednesday, August 18, 2021 15:59:15 Romance Daylight Time

	#	Name	Type	Std. Conc	RT	Area	IS Area	Response	Detecti...	ng/mL	%Dev
1	1	A MSH 2 0-1-1	Anal...		1.39	14373.364		14373.364	bb		
2	2	A MSH 2 0-1-2	Anal...		1.39	14542.664		14542.664	bb		
3	3	A MSH 2 0-1-3	Anal...		1.39	14476.271		14476.271	bb		
4	4	A MSH 2 0-2-1	Anal...		1.39	13785.313		13785.313	bb		
5	5	A MSH 2 0-2-2	Anal...		1.39	13635.054		13635.054	bb		
6	6	A MSH 2 0-2-3	Anal...		1.39	13794.165		13794.165	bb		
7	7	A MSH 2 Blank 1	Blank								
8	8	A MSH 2 15-1-1	Anal...		1.39	14372.942		14372.942	bb		
9	9	A MSH 2 15-1-2	Anal...		1.39	14565.716		14565.716	bb		
10	10	A MSH 2 15-1-3	Anal...		1.39	14534.468		14534.468	bb		
11	11	A MSH 2 15-2-1	Anal...		1.39	13078.553		13078.553	bb		
12	12	A MSH 2 15-2-2	Anal...		1.39	12883.891		12883.891	bb		
13	13	A MSH 2 15-2-3	Anal...		1.39	13213.239		13213.239	bb		
14	14	A MSH 2 Blank 2	Blank								
15	15	A MSH 2 30-1-1	Anal...		1.39	13812.484		13812.484	bb		
16	16	A MSH 2 30-1-2	Anal...		1.39	13550.757		13550.757	bb		
17	17	A MSH 2 30-1-3	Anal...		1.39	13660.965		13660.965	bb		
18	18	A MSH 2 30-2-1	Anal...		1.39	13078.886		13078.886	bb		
19	19	A MSH 2 30-2-2	Anal...		1.39	13048.814		13048.814	bb		
20	20	A MSH 2 30-2-3	Anal...		1.39	13290.737		13290.737	bb		
21	21	A MSH 2 Blank 3	Blank								
22	22	A MSH 2 1-1-1	Anal...		1.39	13076.497		13076.497	bb		
23	23	A MSH 2 1-1-2	Anal...		1.39	13490.843		13490.843	bb		
24	24	A MSH 2 1-1-3	Anal...		1.39	13359.727		13359.727	bb		
25	25	A MSH 2 1-2-1	Anal...		1.39	12930.208		12930.208	bb		
26	26	A MSH 2 1-2-2	Anal...		1.39	12979.922		12979.922	bb		
27	27	A MSH 2 1-2-3	Anal...		1.39	12911.531		12911.531	bb		
28	28	A MSH 2 Blank 4	Blank								
29	29	A MSH 2 2-1-1	Anal...		1.39	11870.403		11870.403	bb		
30	30	A MSH 2 2-1-2	Anal...		1.39	11832.053		11832.053	bb		
31	31	A MSH 2 2-1-3	Anal...		1.39	11552.827		11552.827	bb		
32	32	A MSH 2 2-2-1	Anal...		1.39	18324.371		18324.371	bb		
33	33	A MSH 2 2-2-2	Anal...		1.39	18166.551		18166.551	bb		
34	34	A MSH 2 2-2-3	Anal...		1.39	18478.051		18478.051	bb		
35	35	A MSH 2 Blank 5	Blank								
36	36	A MSH 2 4-1-1	Anal...		1.39	10296.097		10296.097	bb		
37	37	A MSH 2 4-1-2	Anal...		1.39	9985.418		9985.418	bb		
38	38	A MSH 2 4-1-3	Anal...		1.39	9961.325		9961.325	bb		
39	39	A MSH 2 4-2-1	Anal...		1.39	8956.191		8956.191	bb		
40	40	A MSH 2 4-2-2	Anal...		1.39	8805.820		8805.820	bb		
41	41	A MSH 2 4-2-3	Anal...		1.39	8945.634		8945.634	bb		
42	42	A MSH 2 Blank 6	Blank								
43	43	A MSH 2 6-1-1	Anal...		1.39	9667.248		9667.248	bb		
44	44	A MSH 2 6-1-2	Anal...		1.39	9838.114		9838.114	bb		
45	45	A MSH 2 6-1-3	Anal...		1.39	9601.378		9601.378	bb		
46	46	A MSH 2 6-2-1	Anal...		1.39	8696.854		8696.854	bb		
47	47	A MSH 2 6-2-2	Anal...		1.39	8299.785		8299.785	bb		
48	48	A MSH 2 6-2-3	Anal...		1.39	8517.988		8517.988	bb		
49	49	A MSH 2 24-1-1	Anal...		1.39	5304.283		5304.283	bb		
50	50	A MSH 2 24-1-2	Anal...		1.39	5263.518		5263.518	bb		
51	51	A MSH 2 24-1-3	Anal...		1.39	5164.347		5164.347	bb		
52	52	A MSH 2 24-2-1	Anal...		1.39	6618.657		6618.657	bb		
53	53	A MSH 2 24-2-2	Anal...		1.39	6124.943		6124.943	bb		
54	54	A MSH 2 24-2-3	Anal...		1.39	6172.987		6172.987	bb		
55	55	A MSH 2 Blank 7	Blank								

Method: C:\MassLynx_Projects\Medchem SU.PRO\MethDB\A.mdb 11 Jan 2021 11:59:51
Calibration: 18 Aug 2021 15:58:43

```
Header
Acquired File Name:   A MSH 2 0-1-1
Acquired Date:        17-Aug-2021
Acquired Time:        21:01:17
Job Code:             MSH 20210817 - N - 216 - 75 - A
Task Code:
User Name:
Laboratory Name:
Instrument:           ACQ-TQD#QBA320
Conditions:
Submitter:
SampleID:             A MSH 2 1
Bottle Number:        2:64
Description:
Instrument Calibration:
Calibration File: C:\MassLynx\IntelliStart\Results\Unit Mass Resolution\Calib 20161209.cal
Parameters
MS1 Static:
Mass:             20 Da to 1974 Da.
Resolution:       15.0/15.0
Ion Energy:       0.5
Reference File:   Naics2
Acquisition File: STATMS1
MS1 Scanning:
Mass:             15 Da to 2048 Da.
Resolution:       15.0/15.0
Ion Energy:       0.5
Reference File:   Naics2
Acquisition File: SCNMS1
MS1 Scan Speed Compensation:
Scan:             339 to 2000 amu/sec.
Resolution:       15.0/15.0
Ion Energy:       0.5
Reference File:   Naics2
Acquisition File: FASTMS1
MS2 Static:
Mass:             20 Da to 1974 Da.
Resolution:       15.0/15.0
Ion Energy:       0.5
Reference File:   Naics2
Acquisition File: STATMS2
MS2 Scanning:
Mass:             15 Da to 2048 Da.
Resolution:       15.0/15.0
Ion Energy:       0.5
Reference File:   Naics2
Acquisition File: SCNMS2
MS2 Scan Speed Compensation:
Scan:             339 to 10165 amu/sec.
Resolution:       15.0/15.0
Ion Energy:       0.5
Reference File:   Naics2
Acquisition File: FASTMS2
Calibration Time: 10:33
Calibration Date: 12/09/16
Coefficients
MS1 Static:       -0.000000000000*x^4 + 0.000000000843*x^3 + -0.000001360559*x^2 + 1.000940122634*x
 +-0.253266439024
MS2 Static:       -0.000000000000*x^4 + 0.000000000573*x^3 + -0.000001239648*x^2 + 1.000902600483*x
 +-0.192715155436
Function 1:       None
Function 2:       None
Parameters for C:\Documents and Settings\Administrator\Desktop\old desktop\log D\EA.EXP
Data Processing:
SIR / MRM Chromatogram Spike Removal      ON
```

```
SIR / MRM Smoothing           OFF
Smoothing window size (scans) 3
Number of smooths       2
Prescan Statistics:
Initial Average Intensity     21.8547
Initial Average Std Dev       2.0631
Bunch Zero Level        0.0016
Bunch Std Dev                 0.0064
Bunch Threshold               0.0511
Spike Removal Std Dev         1.7324
Method Events:
Initial Stop Flow:      No Change
Initial Switch 2: No Change
Initial Switch 3: No Change
Initial Switch 4: No Change
Initial Infusion: No Change
Initial Flow State:     LC
Initial Flow Rate:      5
Initial Reservoir:      No Action
API Probe Delay Temp:   20
Initial Refill:         No Action
Timed Events Enabled
Event Time  Name        Action
Instrument Parameters - Function 1:
Parameter File - C:\Documents and Settings\Administrator\Desktop\old desktop\log D\log D tune file.
IPR
Polarity    ES+
Calibration Static 2
Capillary (kV)    3.50  3.48
Cone (V)    38.00 32.48
Extractor (V)     3.00  2.20
RF (V)      0.10
Source Temperature (°C) 140   139
Desolvation Temperature (°C)  450   450
Cone Gas Flow (L/Hr)    90    90
Desolvation Gas Flow (L/Hr)   900   901
Collision Gas Flow (mL/Min)   0.20  0.20
LM 1 Resolution   15.50
HM 1 Resolution   14.84
Ion Energy 1      0.30
MS Mode Entrance  50.00
MS Mode Collision Energy      3.00
MS Mode Exit      50.00
MSMS Mode Entrance      -2.00
MSMS Mode Collision Energy    2.00
MSMS Mode Exit    2.00
LM 2 Resolution   15.00
HM 2 Resolution   15.50
Ion Energy 2      1.22
Gain  1.00
Multiplier  -494.34
Active Reservoir  B
Engineers Settings:
MS1 Low Mass Position         518
MS1 High Mass Position        284
MS1 Low Mass Resolution       513
MS1 High Mass Resolution      1732
MS1 Resolution Linearity      834
MS1 High Mass DC Balance      0
MS1 DC Polarity               Positive
MS2 Low Mass Position         519
MS2 High Mass Position        238
MS2 Low Mass Resolution       518
MS2 High Mass Resolution      644
MS2 Resolution Linearity      756
MS2 High Mass DC Balance      -0
MS2 DC Polarity               Positive
HM RF Lens Correction +       0
```

```
HM RF Lens Correction -       0
Inter-scan delays:
Automatic Mode
MS 1 Delay Table:
         R     delay
<=   0.500     0.005
<=   1.200     0.010
<=   2.400     0.015
<=   6.000     0.020
<=  15.000     0.025
<=  25.000     0.028
>   25.000     0.030
MS 2 Delay Table:
         R     delay
<=   2.000     0.005
<=   4.000     0.008
<=   7.000     0.010
<=  10.000     0.012
<=  20.000     0.014
>   20.000     0.016
ACE Experimental Record
Inlet Method File: c:\masslynx_projects\medchem su.pro\acqudb\adme sophie
-------------------   Prerun method parameters   ---------------
Waters ACQUITY QSM
Waters Acquity TUV
 Run Time: 0.20 min
 Wavelength Mode: Single Wavelength
 Lamp On: On
Channel A...
 Comment:
 Wavelength: 254 nm
 Sampling Rate: 20 points/sec
 Data Mode: Absorbance
 Time Constant: 0.1000 sec
 Auto Zero On Wavelength Change: Maintain Baseline
 Auto Zero On Inject Start: Yes
Analog 1...
 Sensitivity: 2.000 AUFS
 Chart Polarity: Positive (+)
 Voltage Offset: 0 mV
 Enable Chart Mark: Yes
 Run Events: Yes
 Pulse Width: 1.0 sec
 Rect Wave Period: 0.2 sec
----------------------------  oOo  ----------------------------
---------------------   Run method parameters   ----------------
Waters ACQUITY QSM
 Solvent A Name: Water
 Solvent B Name: Acetonitrile
 Solvent C Name: water 1 % FA
 Solvent D Name:
 Low Pressure Limit: 0 psi
 High Pressure Limit: 15000 psi
 Seal Wash Period: 5.00 min
 [Gradient Table]
  Time(min) Flow Rate(mL/min) %A %B %C %D Curve
 1. Initial 0.700 90.0 5.0 5.0 0.0 Initial
 2. 0.15 0.700 45.0 50.0 5.0 0.0 6
 3. 1.50 0.700 0.0 95.0 5.0 0.0 6
 4. 1.80 0.700 90.0 5.0 5.0 0.0 1
 Comment: ACQUITY UPLC BEH C18 2.1 x 50 mm
 Flow Ramp Rate: 0.45 min
 D Solvent Selection (if supported): No Change
 System Pressure Data Channel: No
 Flow Rate Data Channel: No
 %A Data Channel: No
 %B Data Channel: No
 %C Data Channel: No
```

```
 %D Data Channel: No
 Primary Data Channel: No
 Accumulator Data Channel: No
 Degasser Data Channel: No
 Gradient Start: At Injection
 Gradient Start Volume: 0 uL
 Gradient Start Time: 0.00 min
 Participate in pre-analysis: No
Waters Acquity TUV
 Run Time: 1.80 min
 Wavelength Mode: Single Wavelength
 Lamp On: On
Channel A...
 Comment:
 Wavelength: 214 nm
 Sampling Rate: 20 points/sec
 Data Mode: Absorbance
 Time Constant: 0.1000 sec
 Auto Zero On Wavelength Change: Maintain Baseline
 Auto Zero On Inject Start: Yes
Analog 1...
 Sensitivity: 2.000 AUFS
 Chart Polarity: Positive (+)
 Voltage Offset: 0 mV
 Enable Chart Mark: Yes
 Run Events: Yes
 Pulse Width: 1.0 sec
 Rect Wave Period: 0.2 sec
Waters ACQUITY FTN AutoSampler
 Run Time: 1.80 min
 Comment: ACQUITY UPLC BEH C18 2.1 x 50 mm
 Load Ahead: Disabled
 Loop Offline: Automatic min
 Wash Solvent Name: Acetonitrile
 Pre-Inject Wash Time: 0.0 sec
 Post-Inject Wash Time: 6.0 sec
 Purge Solvent Name: Water
 Dilution: Disabled
 Dilution Volume: 0 uL
 Delay Time: 0 min
 Dilution Needle Placement: 4.0 mm
 Target Column Temperature: 40.0 C
 Column Temperature Alarm Band: Disabled
 Target Sample Temperature: 15.0 C
 Sample Temperature Alarm Band: Disabled
 Syringe Draw Rate: Automatic
 Needle Placement: Automatic
 Pre-Aspirate Air Gap: Automatic
 Post-Aspirate Air Gap: Automatic
 Column Temperature Data Channel: No
 Room Temperature Data Channel: No
 Sample Temperature Data Channel: No
 Sample Organizer Temperature Data Channel: No
 Sample Pressure Data Channel: No
 Preheater Temperature Data Channel: No
 Seal Force Data Channel: No
 No Injection Mode Enabled: No
 Run Events: No
Sample Run Injection Parameter
Injection Volume (ul)   -   3.00
----------------------------  oOo  ----------------------------
End of experimental record.
-------------------- Waters ACQUITY QSM Postrun Report  ---------------
 Firmware Version: 1.50.237 (May 18 2011)
 Software Version: 1.50.1621
 Checksum: 0xae400516
 Serial Number: M09QSM056N
 Minimum System Pressure: 0.0 psi
```

```
 Maximum System Pressure: 0.0 psi
 Average System Pressure: 0.0 psi
----------------------------  oOo  ----------------------------
------------------- Waters ACQUITY FTN Postrun Report  ---------------
 Software Version: 1.50.1481
 Firmware Version: 1.50.317 (Jul 11 2011)
 Checksum: 0x3e83519d
 Serial Number: M09SDI055N
 Sample Syringe Size: 100.0
 Extension Loop Size: 0.0
 Needle Size: 15.0
 Column Type: ACQUITY UPLC® BEH C18 1.7µm
 Column Serial Number: 02343407715781
 Total Injections on Column: 7742
 Minimum Sample Temperature: 0.0
 Maximum Sample Temperature: 0.0
 Average Sample Temperature: 0.0
 Minimum Column Temperature: 40.0
 Maximum Column Temperature: 40.2
 Average Column Temperature: 0.0
----------------------------  oOo  ----------------------------
------------------- Generic Instrument Postrun Report  ---------------
 Software Version: 1.50.2530
 Firmware Version: 1.50.2182 (May 11 2011)
 Checksum: 0xc09b9cb2
 Serial Number: J08UPT460M
 Lamp On/Off Event: No
 Lamp Life: 903.00
 Lamp Serial Number: 000296721
 Flow Cell Type: Other
 Flow Cell Path Length: 0.00 mm
 Flow Cell Volume: 0.00 microliters
 Flow Cell Serial Number: 1
 Flow Cell Part Number: 1
 Optics Temperature Stabilzation Setting: unknown
----------------------------  oOo  ----------------------------
------------------- Waters ACQUITY QSM Postrun Report  ---------------
 Firmware Version: 1.50.237 (May 18 2011)
 Software Version: 1.50.1621
 Checksum: 0xae400516
 Serial Number: M09QSM056N
 Minimum System Pressure: 0.0 psi
 Maximum System Pressure: 0.0 psi
 Average System Pressure: 0.0 psi
----------------------------  oOo  ----------------------------
Function 1
Scans in function:          337
Cycle time (secs):          Automatic
Inter Scan Delay (secs):    Automatic
Inter Channel Delay (secs):Automatic
Span (Da):                  0.500
Start and End Time(mins):    0.300 to 1.800
Ionization mode:            ES+
Data type:                  SIR or MRM data
Function type:              MRM of 1 channel
Chan Reaction               Dwell(secs) Cone Volt. Col.Energy Delay(secs) Compound Formula|Mass Comm
ents
1  : 419.87 > 295.05           0.260       30.0       18.0       Auto      EA       418.9        Inte
lliStart Generated
Function 2
Scans in function:          2161
Function type:              Diode Array
Wavelength range (nm):       214 to 214
```

Dataset: Untitled
Last Altered: Wednesday, August 18, 2021 16:17:53 Romance Daylight Time
Printed: Wednesday, August 18, 2021 16:18:18 Romance Daylight Time

Method: C:\MassLynx_Projects\Medchem SU.PRO\MethDB\A.mdb 11 Jan 2021 11:59:51
Calibration: 18 Aug 2021 16:17:53

Compound name: A
No Calibration
Response type: External Std, Area
Curve type: Linear, Origin: Exclude, Weighting: 1/x, Axis trans: None

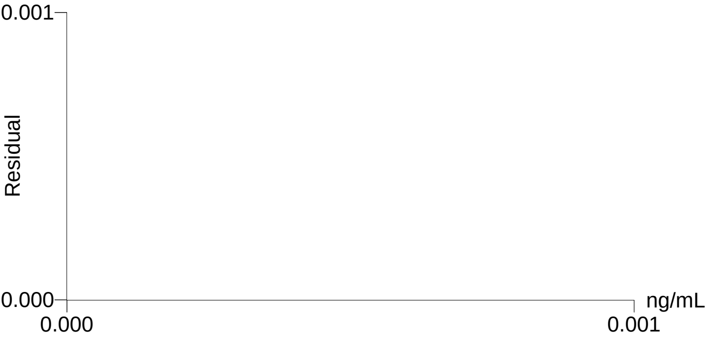

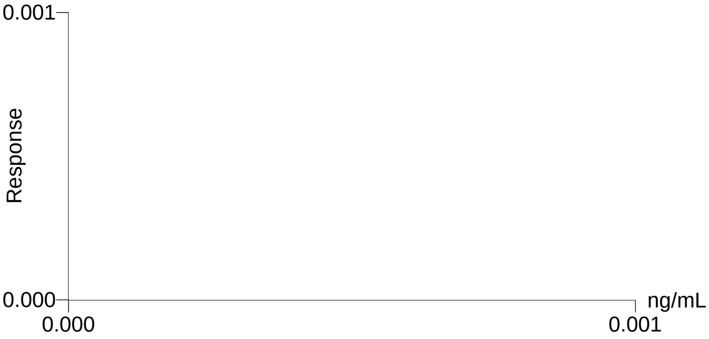

Dataset: Untitled
Last Altered: Wednesday, August 18, 2021 16:17:53 Romance Daylight Time
Printed: Wednesday, August 18, 2021 16:18:18 Romance Daylight Time

Method: C:\MassLynx_Projects\Medchem SU.PRO\MethDB\A.mdb 11 Jan 2021 11:59:51
Calibration: 18 Aug 2021 16:17:53

Sample Name: A MSM 2 0-1-1

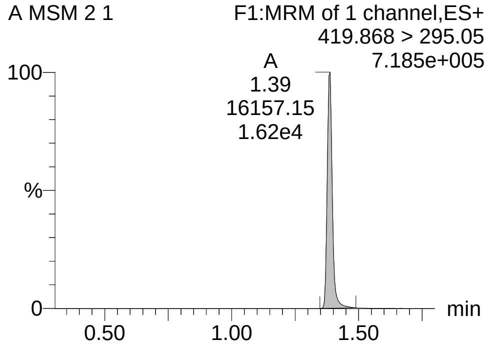

Sample Name: A MSM 2 0-1-2

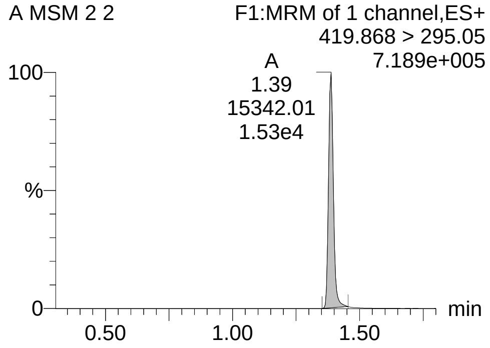

Sample Name: A MSM 2 0-1-3

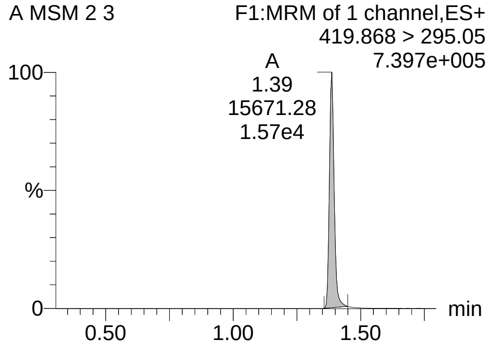

Sample Name: A MSM 2 Blank 1

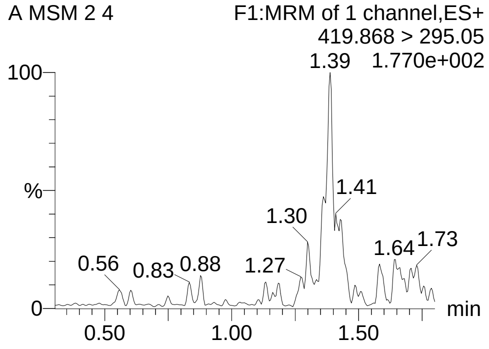

Sample Name: A MSM 2 15-1-1

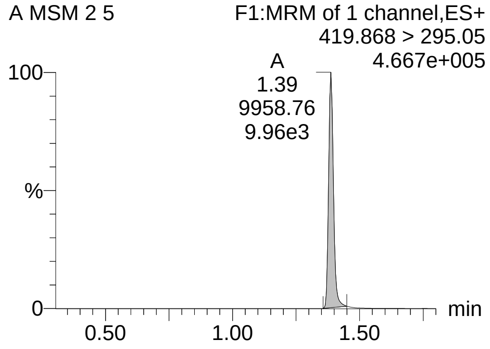

Sample Name: A MSM 2 15-1-2

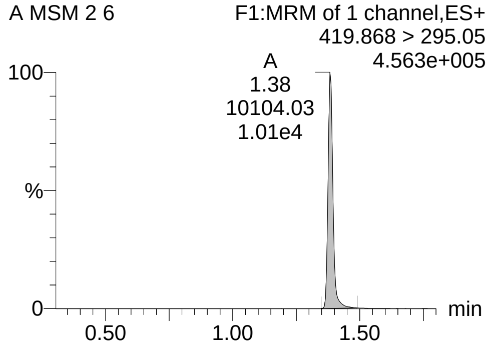

Sample Name: A MSM 2 15-1-3

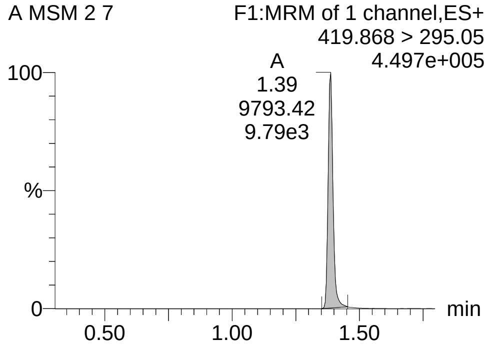

Sample Name: A MSM 2 Blank 2

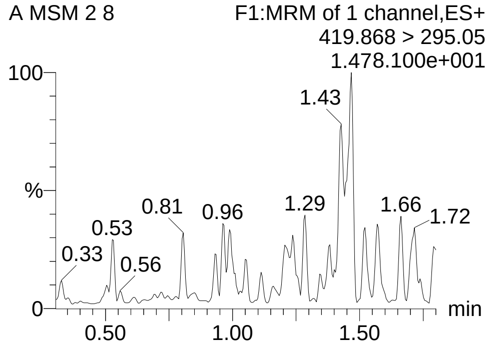

Sample Name: A MSM 2 30-1-1

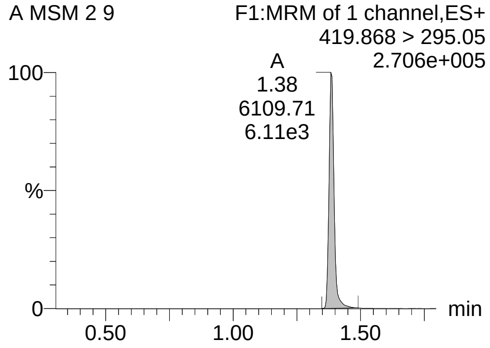

Sample Name: A MSM 2 30-1-2

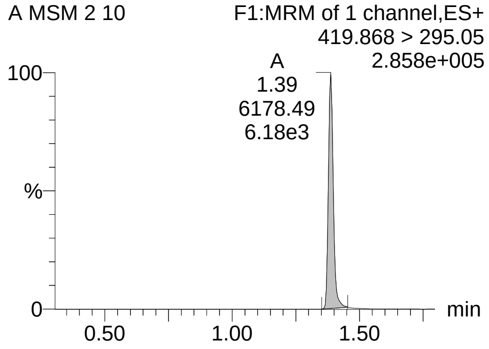

Sample Name: A MSM 2 30-1-3

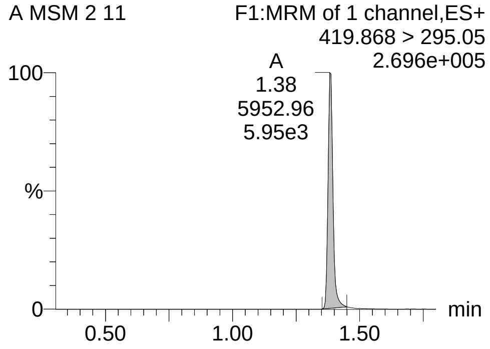

Sample Name: A MSM 2 Blank 3

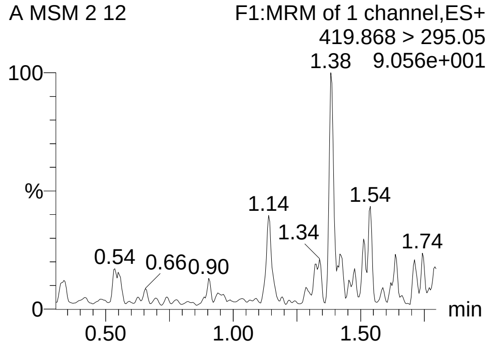

Dataset: Untitled
Last Altered: Wednesday, August 18, 2021 16:17:53 Romance Daylight Time
Printed: Wednesday, August 18, 2021 16:18:18 Romance Daylight Time

Sample Name: A MSM 2 1-1-1

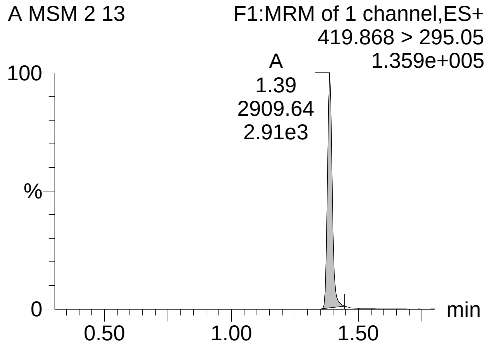

Sample Name: A MSM 2 1-1-2

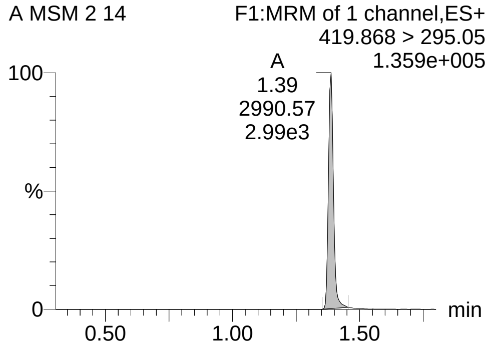

Sample Name: A MSM 2 1-1-3

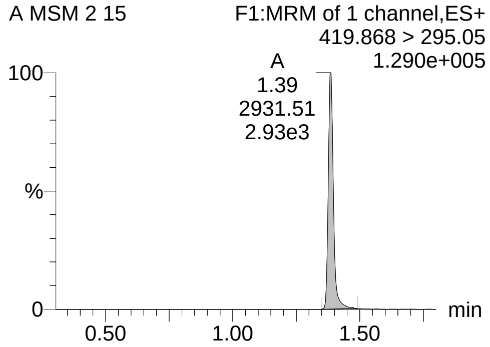

Sample Name: A MSM 3 1-1-1

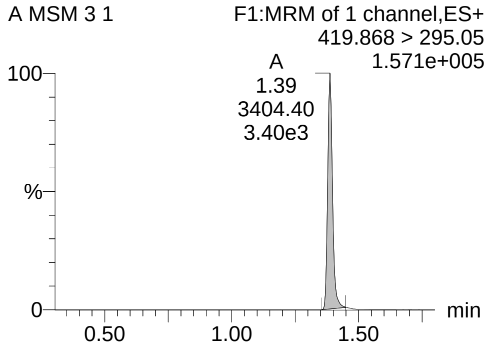

Sample Name: A MSM 3 1-1-2

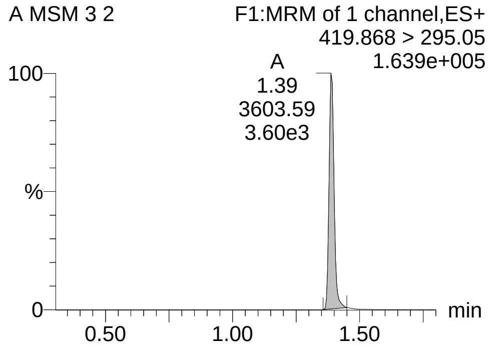

Sample Name: A MSM 3 1-1-3

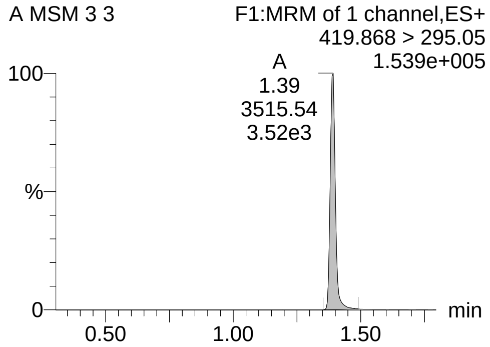

Sample Name: A MSM 2 Blank 4

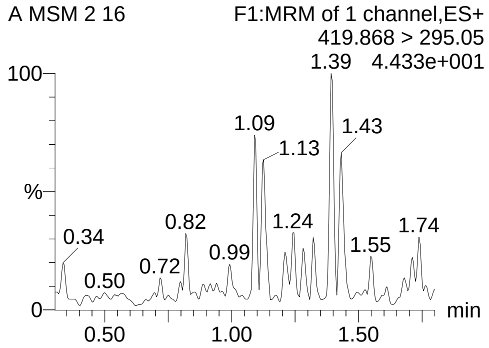

Sample Name: A MSM 2 2-1-1

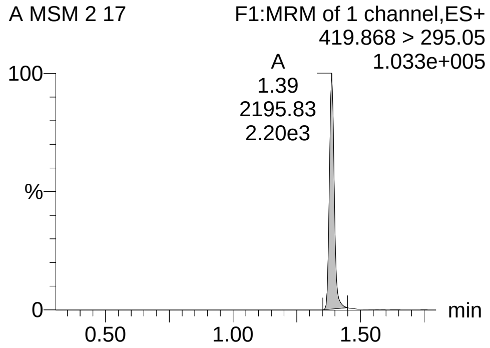

Sample Name: A MSM 2 2-1-2

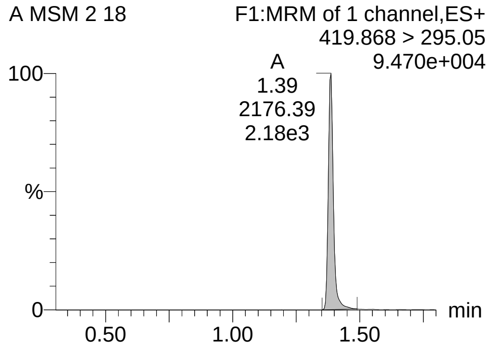

Sample Name: A MSM 2 2-1-3

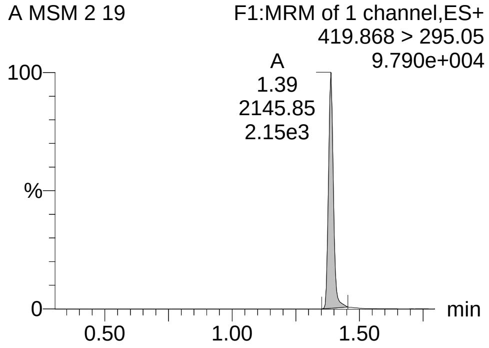

Sample Name: A MSM 2 Blank 5

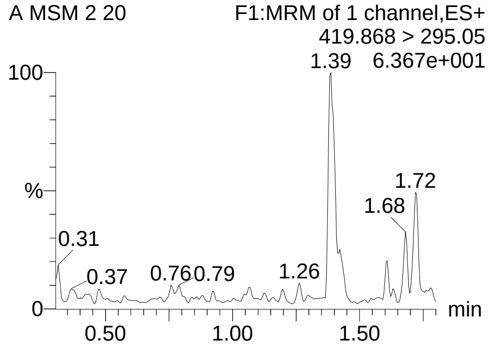

Sample Name: A MSM 2 4-1-1

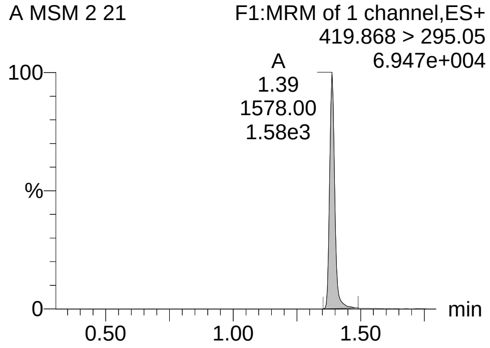

Sample Name: A MSM 2 4-1-2

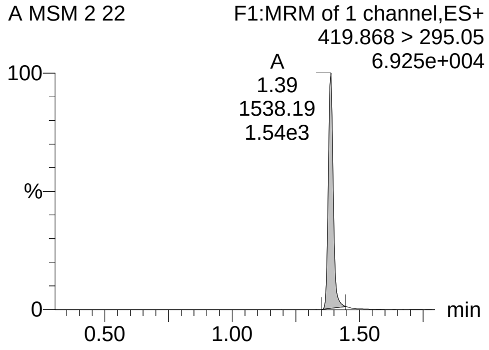

Sample Name: A MSM 2 4-1-3

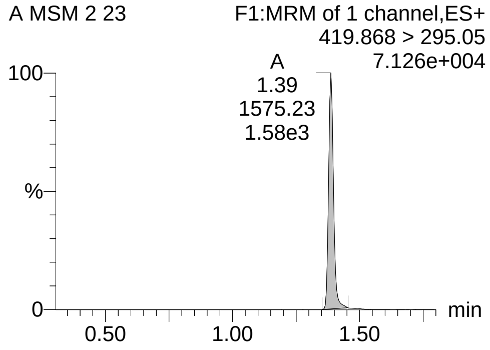

Sample Name: A MSM 2 Blank 6

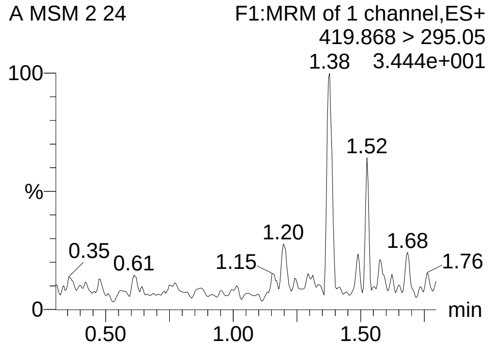

Dataset: Untitled
Last Altered: Wednesday, August 18, 2021 16:17:53 Romance Daylight Time
Printed: Wednesday, August 18, 2021 16:18:18 Romance Daylight Time

Sample Name: A MSM 2 6-1-1

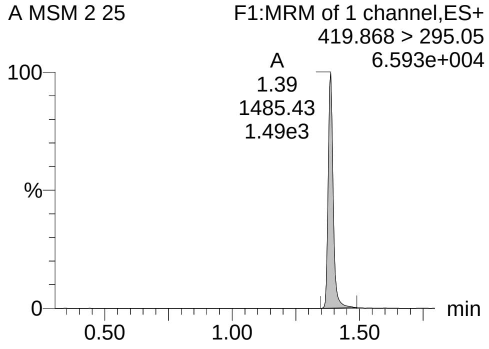

Sample Name: A MSM 2 6-1-2

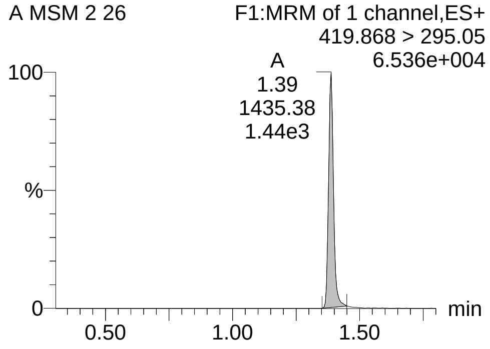

Sample Name: A MSM 2 6-1-3

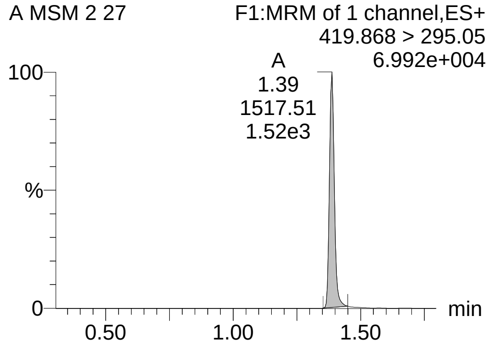

Sample Name: A MSM 2 Blank 7

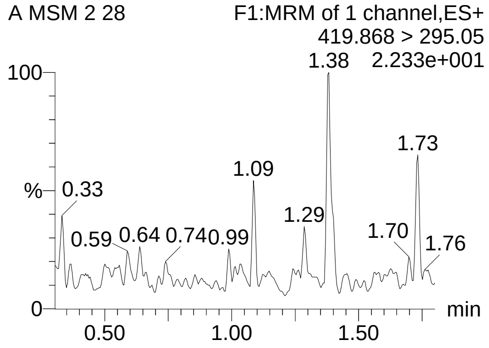

Sample Name: A MSM 2 24-1-1

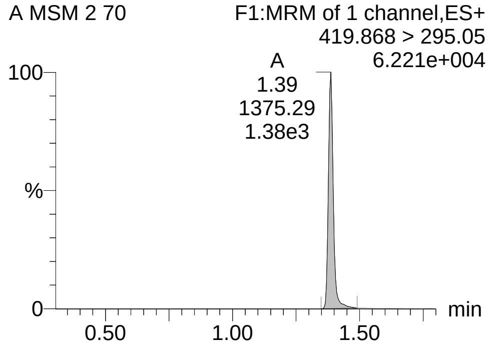

Sample Name: A MSM 2 24-1-2

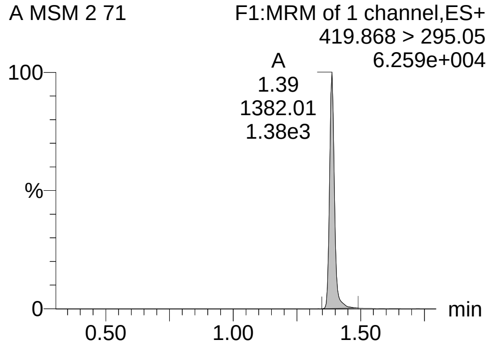

Sample Name: A MSM 2 24-1-3

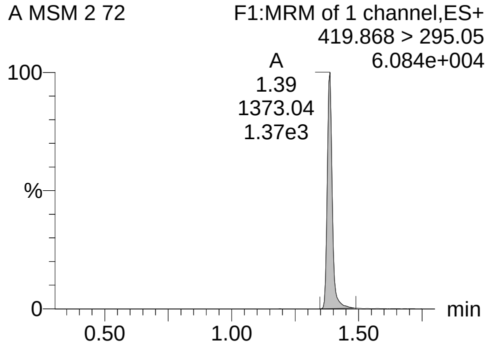

Dataset: Untitled
Last Altered: Wednesday, August 18, 2021 16:17:53 Romance Daylight Time
Printed: Wednesday, August 18, 2021 16:18:18 Romance Daylight Time

	#	Name	Type	Std. Conc	RT	Area	IS Area	Response	Detecti...	ng/mL	%Dev
1	1	A MSM 2 0-1-1	Anal...		1.39	16157.149		16157.149	bb		
2	2	A MSM 2 0-1-2	Anal...		1.39	15342.006		15342.006	bb		
3	3	A MSM 2 0-1-3	Anal...		1.39	15671.279		15671.279	bb		
4	4	A MSM 2 Blank 1	Blank								
5	5	A MSM 2 15-1-1	Anal...		1.39	9958.758		9958.758	bb		
6	6	A MSM 2 15-1-2	Anal...		1.38	10104.032		10104.032	bb		
7	7	A MSM 2 15-1-3	Anal...		1.39	9793.418		9793.418	bb		
8	8	A MSM 2 Blank 2	Blank								
9	9	A MSM 2 30-1-1	Anal...		1.38	6109.706		6109.706	bb		
10	10	A MSM 2 30-1-2	Anal...		1.39	6178.494		6178.494	bb		
11	11	A MSM 2 30-1-3	Anal...		1.38	5952.958		5952.958	bb		
12	12	A MSM 2 Blank 3	Blank								
13	13	A MSM 2 1-1-1	Anal...		1.39	2909.640		2909.640	bb		
14	14	A MSM 2 1-1-2	Anal...		1.39	2990.573		2990.573	bb		
15	15	A MSM 2 1-1-3	Anal...		1.39	2931.514		2931.514	bb		
16	16	A MSM 3 1-1-1	Anal...		1.39	3404.404		3404.404	bb		
17	17	A MSM 3 1-1-2	Anal...		1.39	3603.594		3603.594	bb		
18	18	A MSM 3 1-1-3	Anal...		1.39	3515.543		3515.543	bb		
19	19	A MSM 2 Blank 4	Blank								
20	20	A MSM 2 2-1-1	Anal...		1.39	2195.831		2195.831	bb		
21	21	A MSM 2 2-1-2	Anal...		1.39	2176.388		2176.388	bb		
22	22	A MSM 2 2-1-3	Anal...		1.39	2145.854		2145.854	bb		
23	23	A MSM 2 Blank 5	Blank								
24	24	A MSM 2 4-1-1	Anal...		1.39	1577.997		1577.997	bb		
25	25	A MSM 2 4-1-2	Anal...		1.39	1538.186		1538.186	bb		
26	26	A MSM 2 4-1-3	Anal...		1.39	1575.230		1575.230	bb		
27	27	A MSM 2 Blank 6	Blank								
28	28	A MSM 2 6-1-1	Anal...		1.39	1485.430		1485.430	bb		
29	29	A MSM 2 6-1-2	Anal...		1.39	1435.381		1435.381	bb		
30	30	A MSM 2 6-1-3	Anal...		1.39	1517.512		1517.512	bb		
31	31	A MSM 2 Blank 7	Blank								
32	32	A MSM 2 24-1-1	Anal...		1.39	1375.290		1375.290	bb		
33	33	A MSM 2 24-1-2	Anal...		1.39	1382.014		1382.014	bb		
34	34	A MSM 2 24-1-3	Anal...		1.39	1373.035		1373.035	bb		

Method: C:\MassLynx_Projects\Medchem SU.PRO\MethDB\A.mdb 11 Jan 2021 11:59:51
Calibration: 18 Aug 2021 16:17:53

```
Header
Acquired File Name:   A MSM 2 0-1-1
Acquired Date:        16-Aug-2021
Acquired Time:        21:04:16
Job Code:             MSM 20210816 - N - 216 - 75 - A
Task Code:
User Name:
Laboratory Name:
Instrument:           ACQ-TQD#QBA320
Conditions:
Submitter:
SampleID:             A MSM 2 1
Bottle Number:        2:64
Description:
Instrument Calibration:
Calibration File: C:\MassLynx\IntelliStart\Results\Unit Mass Resolution\Calib 20161209.cal
Parameters
MS1 Static:
Mass:             20 Da to 1974 Da.
Resolution:       15.0/15.0
Ion Energy:       0.5
Reference File:   Naics2
Acquisition File: STATMS1
MS1 Scanning:
Mass:             15 Da to 2048 Da.
Resolution:       15.0/15.0
Ion Energy:       0.5
Reference File:   Naics2
Acquisition File: SCNMS1
MS1 Scan Speed Compensation:
Scan:             339 to 2000 amu/sec.
Resolution:       15.0/15.0
Ion Energy:       0.5
Reference File:   Naics2
Acquisition File: FASTMS1
MS2 Static:
Mass:             20 Da to 1974 Da.
Resolution:       15.0/15.0
Ion Energy:       0.5
Reference File:   Naics2
Acquisition File: STATMS2
MS2 Scanning:
Mass:             15 Da to 2048 Da.
Resolution:       15.0/15.0
Ion Energy:       0.5
Reference File:   Naics2
Acquisition File: SCNMS2
MS2 Scan Speed Compensation:
Scan:             339 to 10165 amu/sec.
Resolution:       15.0/15.0
Ion Energy:       0.5
Reference File:   Naics2
Acquisition File: FASTMS2
Calibration Time: 10:33
Calibration Date: 12/09/16
Coefficients
MS1 Static:       -0.000000000000*x^4 + 0.000000000843*x^3 + -0.000001360559*x^2 + 1.000940122634*x
 +-0.253266439024
MS2 Static:       -0.000000000000*x^4 + 0.000000000573*x^3 + -0.000001239648*x^2 + 1.000902600483*x
 +-0.192715155436
Function 1:       None
Function 2:       None
Parameters for C:\Documents and Settings\Administrator\Desktop\old desktop\log D\EA.EXP
Data Processing:
SIR / MRM Chromatogram Spike Removal      ON
```

```
SIR / MRM Smoothing           OFF
Smoothing window size (scans) 3
Number of smooths       2
Prescan Statistics:
Initial Average Intensity     21.8777
Initial Average Std Dev       1.7606
Bunch Zero Level        0.0018
Bunch Std Dev                 0.0066
Bunch Threshold               0.0526
Spike Removal Std Dev         1.7559
Method Events:
Initial Stop Flow:      No Change
Initial Switch 2: No Change
Initial Switch 3: No Change
Initial Switch 4: No Change
Initial Infusion: No Change
Initial Flow State:     LC
Initial Flow Rate:      5
Initial Reservoir:      No Action
API Probe Delay Temp:   20
Initial Refill:         No Action
Timed Events Enabled
Event Time  Name        Action
Instrument Parameters - Function 1:
Parameter File - C:\Documents and Settings\Administrator\Desktop\old desktop\log D\log D tune file.
IPR
Polarity    ES+
Calibration Static 2
Capillary (kV)    3.50  3.48
Cone (V)    38.00 31.01
Extractor (V)     3.00  2.20
RF (V)      0.10
Source Temperature (°C) 140   138
Desolvation Temperature (°C)  450   440
Cone Gas Flow (L/Hr)    90    90
Desolvation Gas Flow (L/Hr)   900   900
Collision Gas Flow (mL/Min)   0.20  0.20
LM 1 Resolution   15.50
HM 1 Resolution   14.84
Ion Energy 1      0.30
MS Mode Entrance  50.00
MS Mode Collision Energy      3.00
MS Mode Exit      50.00
MSMS Mode Entrance      -2.00
MSMS Mode Collision Energy    2.00
MSMS Mode Exit    2.00
LM 2 Resolution   15.00
HM 2 Resolution   15.50
Ion Energy 2      1.22
Gain  1.00
Multiplier  -493.56
Active Reservoir  B
Engineers Settings:
MS1 Low Mass Position         518
MS1 High Mass Position        284
MS1 Low Mass Resolution       513
MS1 High Mass Resolution      1732
MS1 Resolution Linearity      834
MS1 High Mass DC Balance      0
MS1 DC Polarity               Positive
MS2 Low Mass Position         519
MS2 High Mass Position        238
MS2 Low Mass Resolution       518
MS2 High Mass Resolution      644
MS2 Resolution Linearity      756
MS2 High Mass DC Balance      -0
MS2 DC Polarity               Positive
HM RF Lens Correction +       0
```

```
HM RF Lens Correction -       0
Inter-scan delays:
Automatic Mode
MS 1 Delay Table:
         R     delay
<=   0.500     0.005
<=   1.200     0.010
<=   2.400     0.015
<=   6.000     0.020
<=  15.000     0.025
<=  25.000     0.028
>   25.000     0.030
MS 2 Delay Table:
         R     delay
<=   2.000     0.005
<=   4.000     0.008
<=   7.000     0.010
<=  10.000     0.012
<=  20.000     0.014
>   20.000     0.016
ACE Experimental Record
Inlet Method File: c:\masslynx_projects\medchem su.pro\acqudb\adme sophie
-------------------   Prerun method parameters   ---------------
Waters ACQUITY QSM
Waters Acquity TUV
 Run Time: 0.20 min
 Wavelength Mode: Single Wavelength
 Lamp On: On
Channel A...
 Comment:
 Wavelength: 254 nm
 Sampling Rate: 20 points/sec
 Data Mode: Absorbance
 Time Constant: 0.1000 sec
 Auto Zero On Wavelength Change: Maintain Baseline
 Auto Zero On Inject Start: Yes
Analog 1...
 Sensitivity: 2.000 AUFS
 Chart Polarity: Positive (+)
 Voltage Offset: 0 mV
 Enable Chart Mark: Yes
 Run Events: Yes
 Pulse Width: 1.0 sec
 Rect Wave Period: 0.2 sec
----------------------------  oOo  ---------------------------
---------------------   Run method parameters   ----------------
Waters ACQUITY QSM
 Solvent A Name: Water
 Solvent B Name: Acetonitrile
 Solvent C Name: water 1 % FA
 Solvent D Name:
 Low Pressure Limit: 0 psi
 High Pressure Limit: 15000 psi
 Seal Wash Period: 5.00 min
 [Gradient Table]
  Time(min) Flow Rate(mL/min) %A %B %C %D Curve
 1. Initial 0.700 90.0 5.0 5.0 0.0 Initial
 2. 0.15 0.700 45.0 50.0 5.0 0.0 6
 3. 1.50 0.700 0.0 95.0 5.0 0.0 6
 4. 1.80 0.700 90.0 5.0 5.0 0.0 1
 Comment: ACQUITY UPLC BEH C18 2.1 x 50 mm
 Flow Ramp Rate: 0.45 min
 D Solvent Selection (if supported): No Change
 System Pressure Data Channel: No
 Flow Rate Data Channel: No
 %A Data Channel: No
 %B Data Channel: No
 %C Data Channel: No
```

```
 %D Data Channel: No
 Primary Data Channel: No
 Accumulator Data Channel: No
 Degasser Data Channel: No
 Gradient Start: At Injection
 Gradient Start Volume: 0 uL
 Gradient Start Time: 0.00 min
 Participate in pre-analysis: No
Waters Acquity TUV
 Run Time: 1.80 min
 Wavelength Mode: Single Wavelength
 Lamp On: On
Channel A...
 Comment:
 Wavelength: 214 nm
 Sampling Rate: 20 points/sec
 Data Mode: Absorbance
 Time Constant: 0.1000 sec
 Auto Zero On Wavelength Change: Maintain Baseline
 Auto Zero On Inject Start: Yes
Analog 1...
 Sensitivity: 2.000 AUFS
 Chart Polarity: Positive (+)
 Voltage Offset: 0 mV
 Enable Chart Mark: Yes
 Run Events: Yes
 Pulse Width: 1.0 sec
 Rect Wave Period: 0.2 sec
Waters ACQUITY FTN AutoSampler
 Run Time: 1.80 min
 Comment: ACQUITY UPLC BEH C18 2.1 x 50 mm
 Load Ahead: Disabled
 Loop Offline: Automatic min
 Wash Solvent Name: Acetonitrile
 Pre-Inject Wash Time: 0.0 sec
 Post-Inject Wash Time: 6.0 sec
 Purge Solvent Name: Water
 Dilution: Disabled
 Dilution Volume: 0 uL
 Delay Time: 0 min
 Dilution Needle Placement: 4.0 mm
 Target Column Temperature: 40.0 C
 Column Temperature Alarm Band: Disabled
 Target Sample Temperature: 15.0 C
 Sample Temperature Alarm Band: Disabled
 Syringe Draw Rate: Automatic
 Needle Placement: Automatic
 Pre-Aspirate Air Gap: Automatic
 Post-Aspirate Air Gap: Automatic
 Column Temperature Data Channel: No
 Room Temperature Data Channel: No
 Sample Temperature Data Channel: No
 Sample Organizer Temperature Data Channel: No
 Sample Pressure Data Channel: No
 Preheater Temperature Data Channel: No
 Seal Force Data Channel: No
 No Injection Mode Enabled: No
 Run Events: No
Sample Run Injection Parameter
Injection Volume (ul)   -   3.00
----------------------------  oOo  -----------------------------
End of experimental record.
-------------------- Waters ACQUITY QSM Postrun Report  ---------------
 Firmware Version: 1.50.237 (May 18 2011)
 Software Version: 1.50.1621
 Checksum: 0xae400516
 Serial Number: M09QSM056N
 Minimum System Pressure: 0.0 psi
```

```
 Maximum System Pressure: 0.0 psi
 Average System Pressure: 0.0 psi
----------------------------  oOo  -----------------------------
------------------- Waters ACQUITY FTN Postrun Report  ---------------
 Software Version: 1.50.1481
 Firmware Version: 1.50.317 (Jul 11 2011)
 Checksum: 0x3e83519d
 Serial Number: M09SDI055N
 Sample Syringe Size: 100.0
 Extension Loop Size: 0.0
 Needle Size: 15.0
 Column Type: ACQUITY UPLC® BEH C18 1.7µm
 Column Serial Number: 02343407715781
 Total Injections on Column: 7503
 Minimum Sample Temperature: 0.0
 Maximum Sample Temperature: 0.0
 Average Sample Temperature: 0.0
 Minimum Column Temperature: 40.0
 Maximum Column Temperature: 40.1
 Average Column Temperature: 0.0
----------------------------  oOo  -----------------------------
------------------- Generic Instrument Postrun Report  ---------------
 Software Version: 1.50.2530
 Firmware Version: 1.50.2182 (May 11 2011)
 Checksum: 0xc09b9cb2
 Serial Number: J08UPT460M
 Lamp On/Off Event: No
 Lamp Life: 879.00
 Lamp Serial Number: 000296721
 Flow Cell Type: Other
 Flow Cell Path Length: 0.00 mm
 Flow Cell Volume: 0.00 microliters
 Flow Cell Serial Number: 1
 Flow Cell Part Number: 1
 Optics Temperature Stabilzation Setting: unknown
----------------------------  oOo  -----------------------------
------------------- Waters ACQUITY QSM Postrun Report  ---------------
 Firmware Version: 1.50.237 (May 18 2011)
 Software Version: 1.50.1621
 Checksum: 0xae400516
 Serial Number: M09QSM056N
 Minimum System Pressure: 0.0 psi
 Maximum System Pressure: 0.0 psi
 Average System Pressure: 0.0 psi
----------------------------  oOo  -----------------------------
Function 1
Scans in function:          338
Cycle time (secs):          Automatic
Inter Scan Delay (secs):    Automatic
Inter Channel Delay (secs):Automatic
Span (Da):                  0.500
Start and End Time(mins):    0.300 to 1.800
Ionization mode:            ES+
Data type:                  SIR or MRM data
Function type:              MRM of 1 channel
Chan Reaction               Dwell(secs) Cone Volt. Col.Energy Delay(secs) Compound Formula|Mass Comm
ents
1  : 419.87 > 295.05           0.260       30.0       18.0       Auto      EA       418.9        Inte
lliStart Generated
Function 2
Scans in function:          2161
Function type:              Diode Array
Wavelength range (nm):       214 to 214
```

Method: C:\MassLynx_Projects\Medchem SU.PRO\MethDB\A.mdb 11 Jan 2021 10:59:51
Calibration: 11 Jan 2021 11:00:12

Compound name: A
No Calibration
Response type: External Std, Area
Curve type: Linear, Origin: Exclude, Weighting: 1/x, Axis trans: None

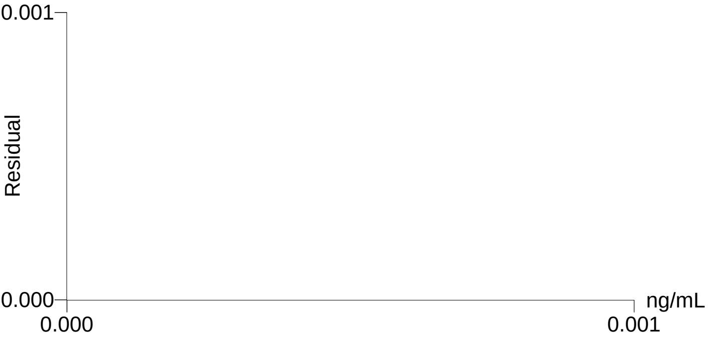

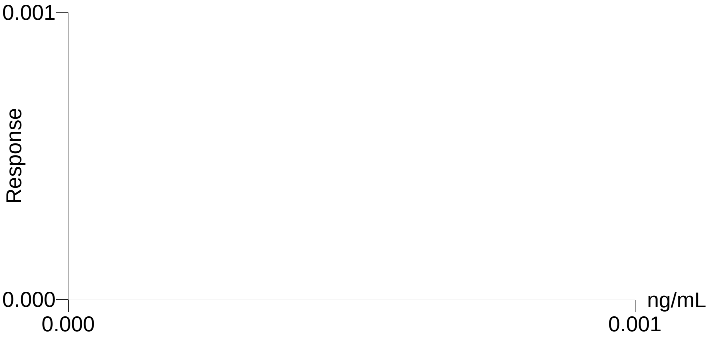

Dataset: Untitled
Last Altered: Monday, January 11, 2021 11:02:27 Romance Standard Time
Printed: Monday, January 11, 2021 11:02:34 Romance Standard Time

Method: C:\MassLynx_Projects\Medchem SU.PRO\MethDB\A.mdb 11 Jan 2021 10:59:51
Calibration: 11 Jan 2021 11:00:12

Sample Name: A MSM 0-1-1

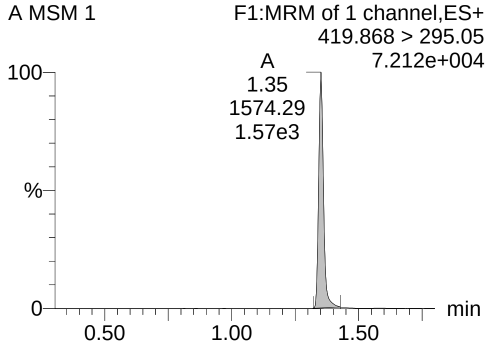

Sample Name: A MSM 0-1-2

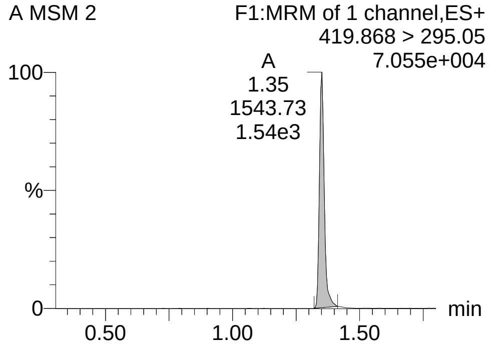

Sample Name: A MSM 0-1-3

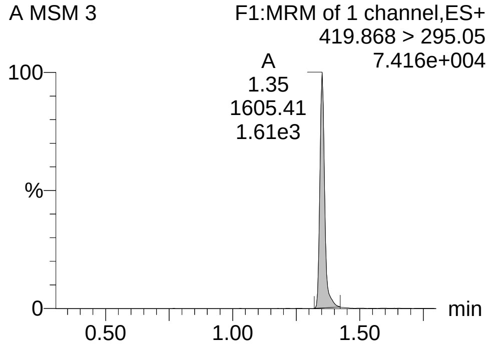

Sample Name: A MSM 0-2-1

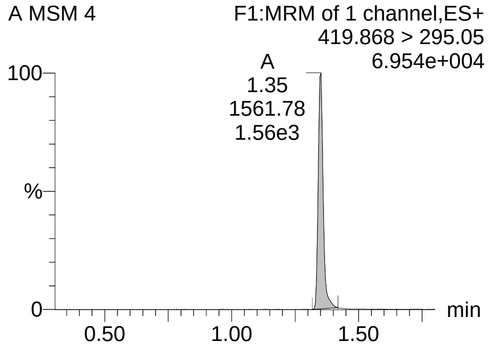

Sample Name: A MSM 0-2-2

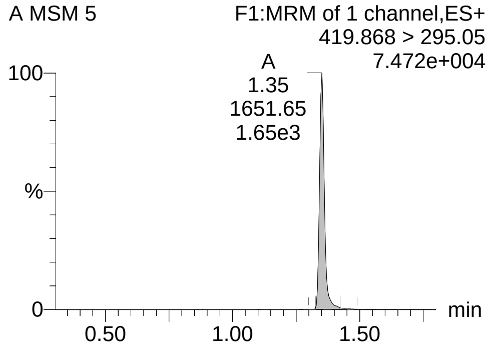

Sample Name: A MSM 0-2-3

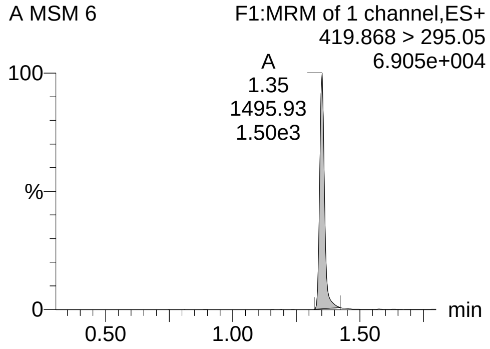

Sample Name: A MSM Blank 1

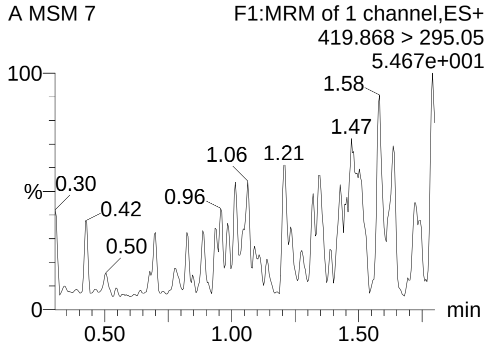

Sample Name: A MSM 15-1-1

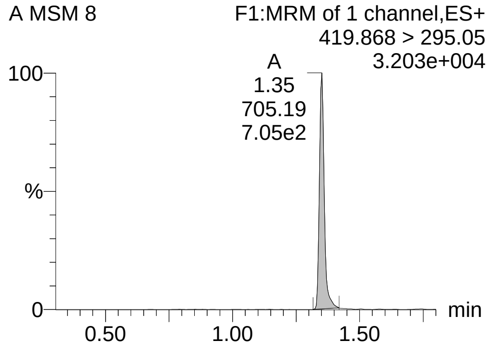

Sample Name: A MSM 15-1-2

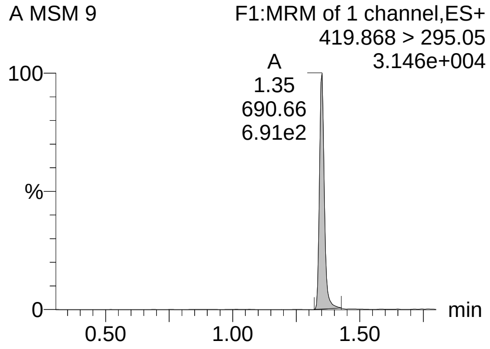

Sample Name: A MSM 15-1-3

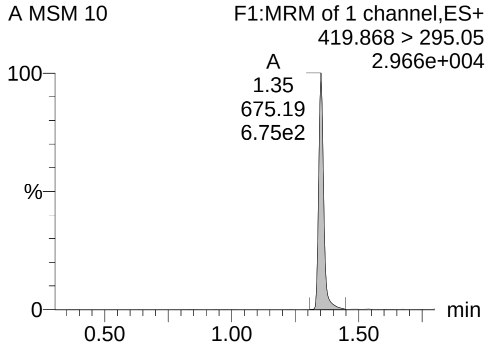

Sample Name: A MSM 15-2-1

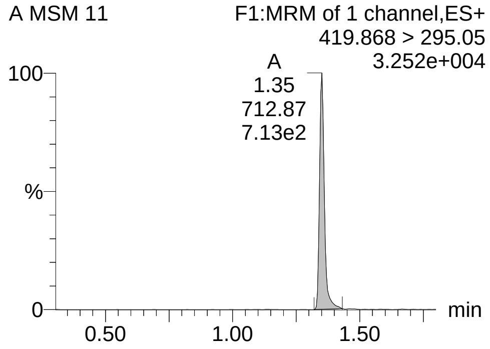

Sample Name: A MSM 15-2-2

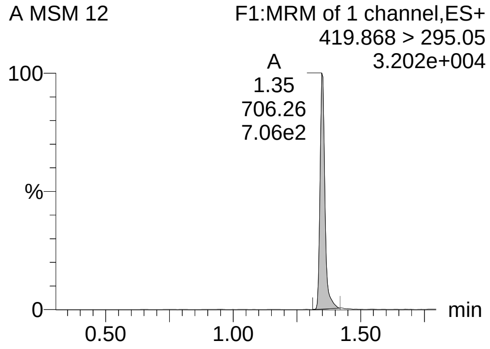

Sample Name: A MSM 15-2-3

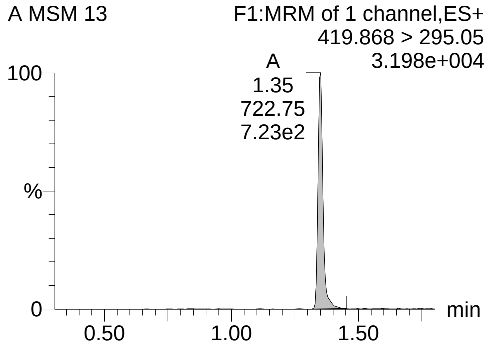

Sample Name: A MSM Blank 2

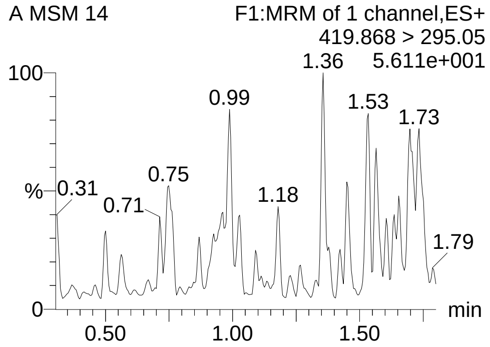

Sample Name: A MSM 30-1-1

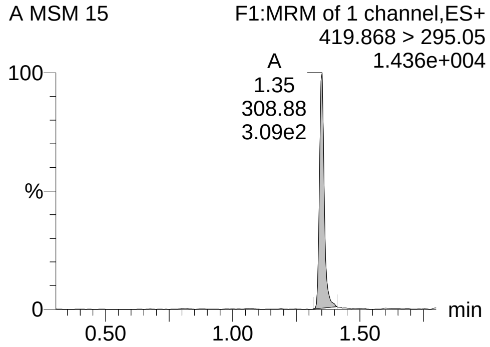

Sample Name: A MSM 30-1-2

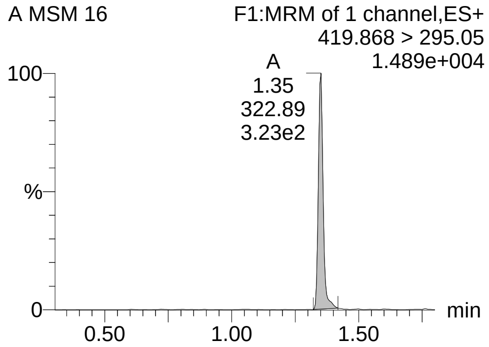

Sample Name: A MSM 30-1-3

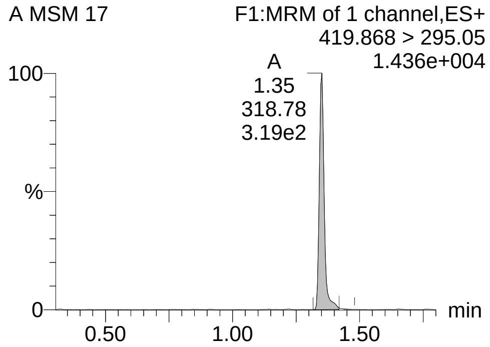

Sample Name: A MSM 30-2-1

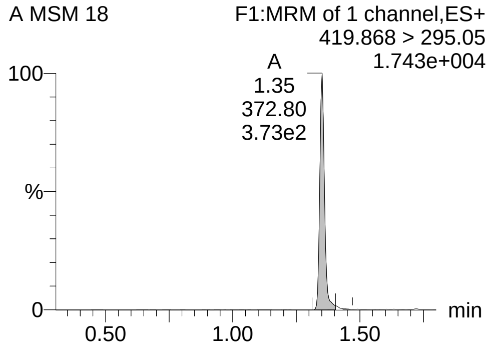

Sample Name: A MSM 30-2-2

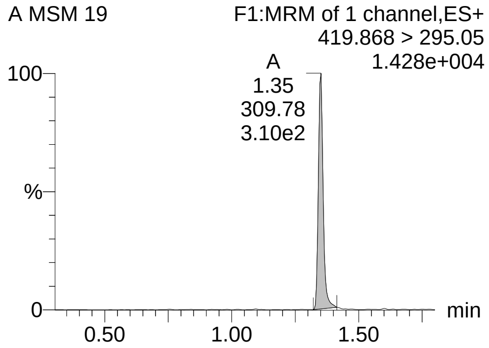

Sample Name: A MSM 30-2-3

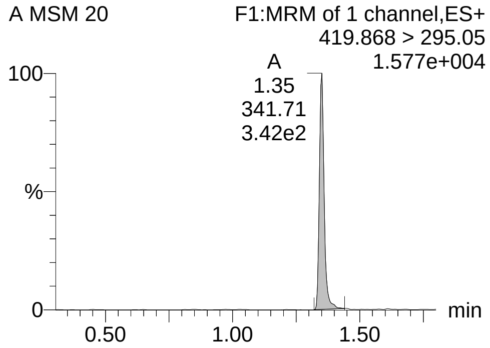

Sample Name: A MSM Blank 3

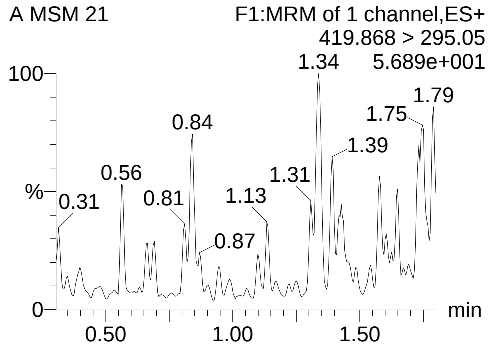

Sample Name: A MSM 1-1-1

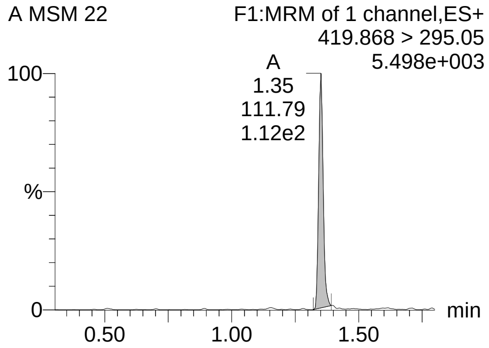

Sample Name: A MSM 1-1-2

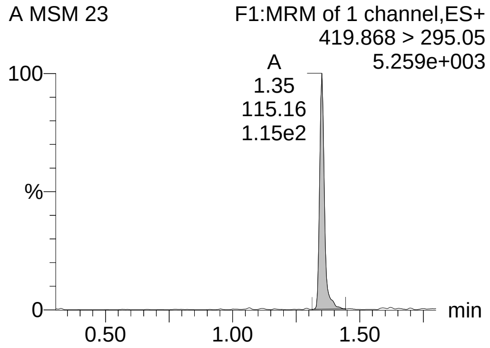

Sample Name: A MSM 1-1-3

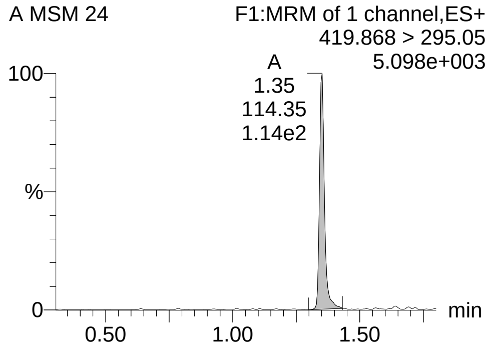

Sample Name: A MSM 1-2-1

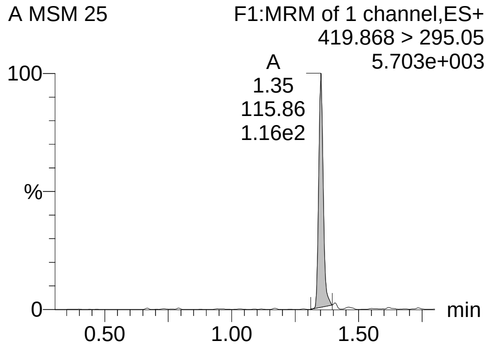

Sample Name: A MSM 1-2-2

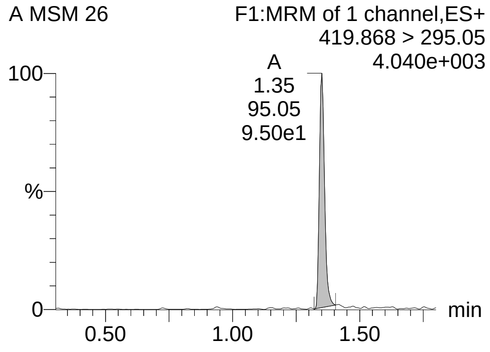

Sample Name: A MSM 1-2-3

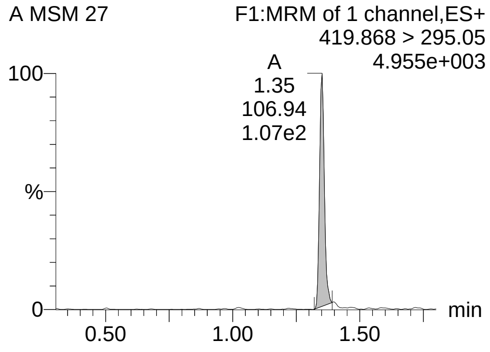

Sample Name: A MSM Blank 4

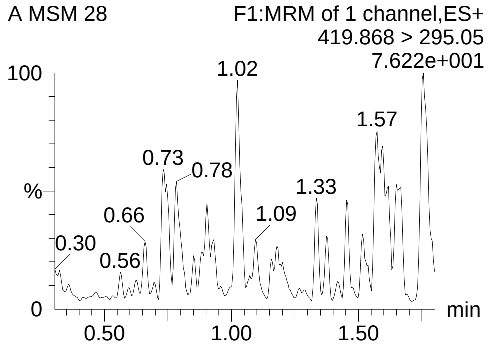

Sample Name: A MSM 2-1-1

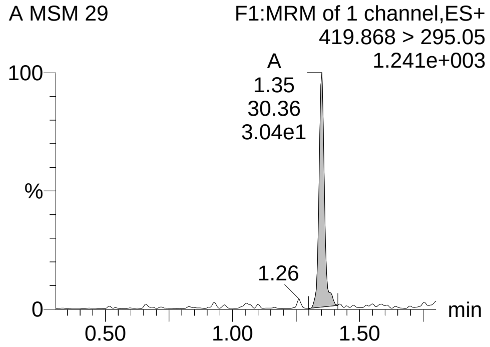

Sample Name: A MSM 2-1-2

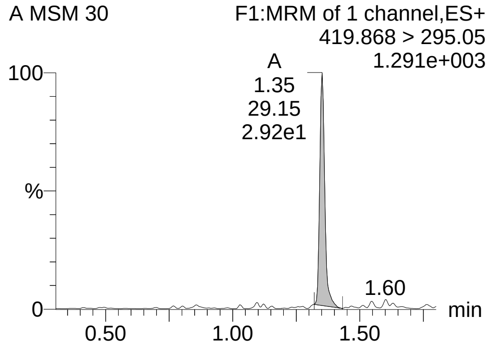

Sample Name: A MSM 2-1-3

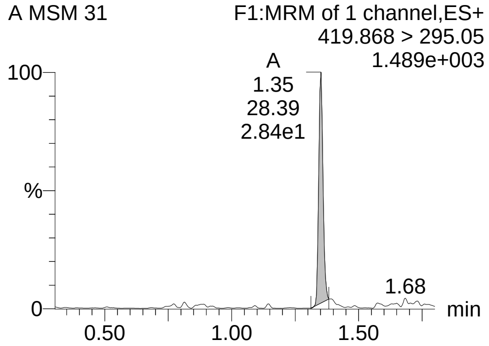

Sample Name: A MSM 2-2-1

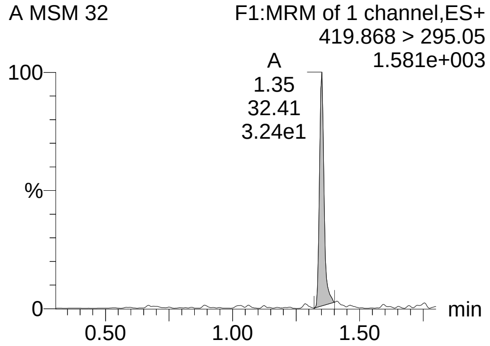

Sample Name: A MSM 2-2-2

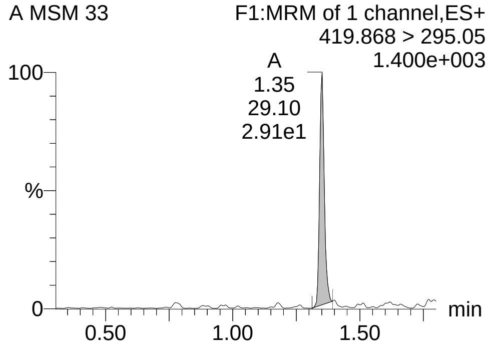

Sample Name: A MSM 2-2-3

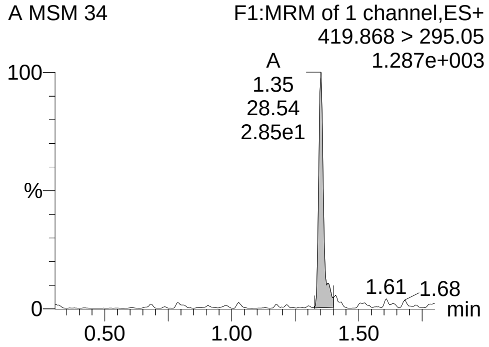

Sample Name: A MSM Blank 5

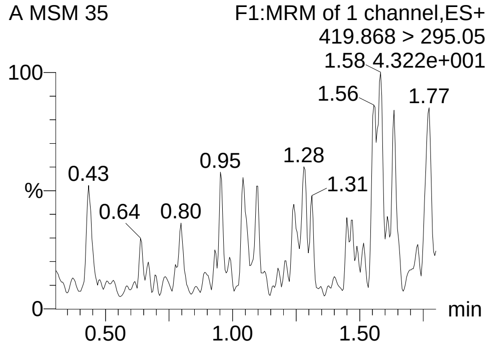

Sample Name: A MSM 4-1-1

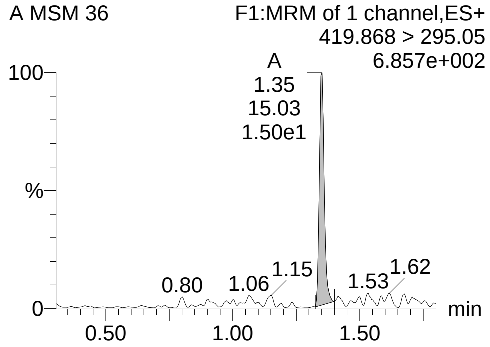

Sample Name: A MSM 4-1-2

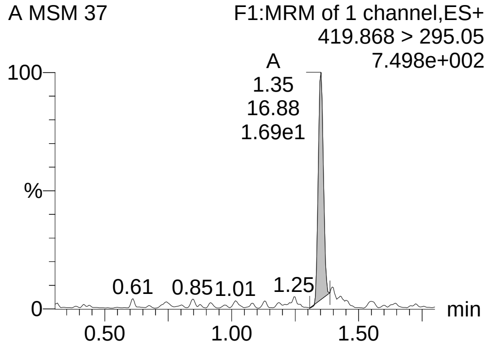

Sample Name: A MSM 4-1-3

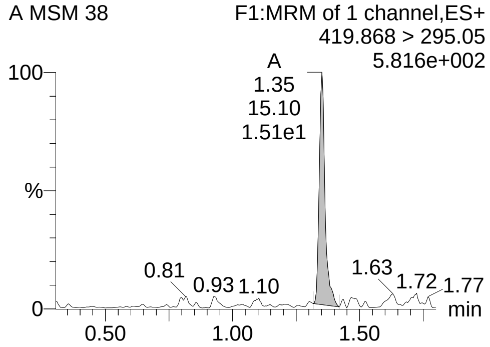

Sample Name: A MSM 4-2-1

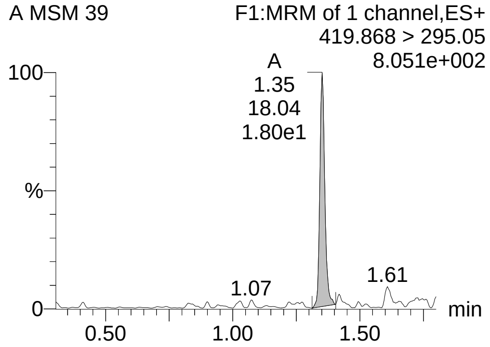

Sample Name: A MSM 4-2-2

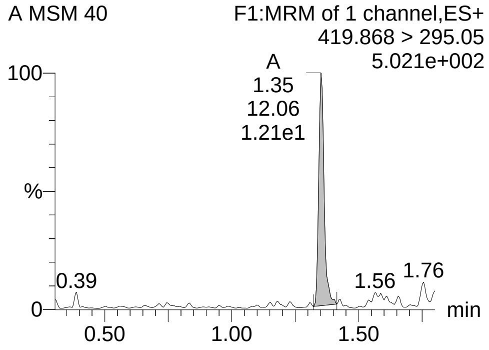

Sample Name: A MSM 4-2-3

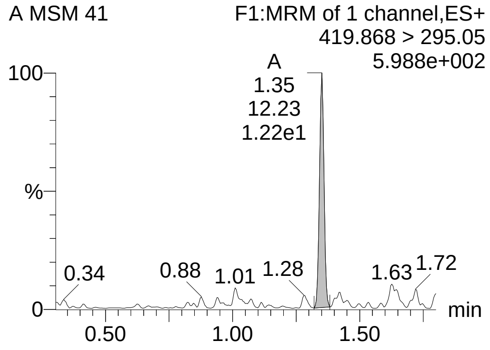

Sample Name: A MSM Blank 6

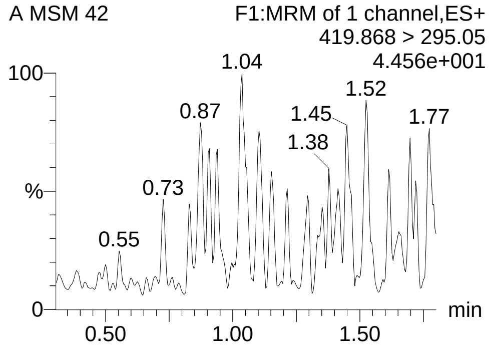

Dataset: Untitled
Last Altered: Monday, January 11, 2021 11:02:27 Romance Standard Time
Printed: Monday, January 11, 2021 11:02:34 Romance Standard Time

Sample Name: A MSM 6-1-1
A MSM 43 F1:MRM of 1 channel,ES+
419.868 > 295.05
1.123e+003
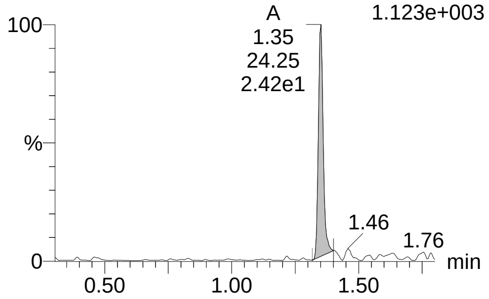

Sample Name: A MSM 6-1-2
A MSM 44 F1:MRM of 1 channel,ES+
419.868 > 295.05
1.083e+003
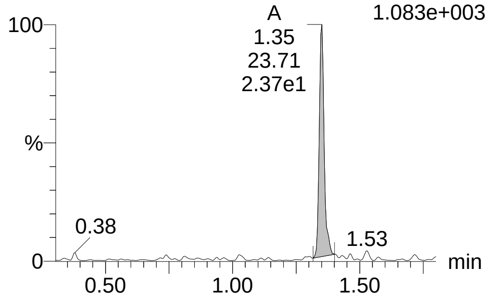

Sample Name: A MSM 6-1-3
A MSM 45 F1:MRM of 1 channel,ES+
419.868 > 295.05
1.125e+003
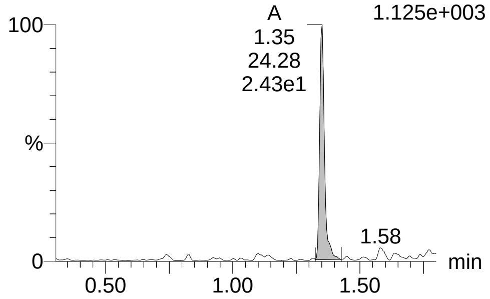

Sample Name: A MSM 6-2-1
A MSM 46 F1:MRM of 1 channel,ES+
419.868 > 295.05
7.276e+002
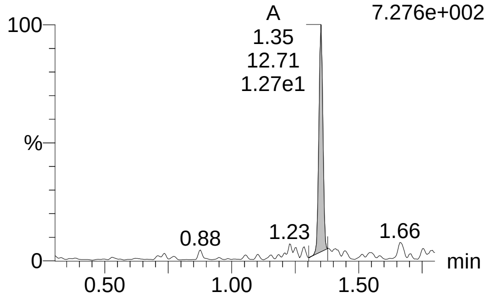

Sample Name: A MSM 6-2-2
A MSM 47 F1:MRM of 1 channel,ES+
419.868 > 295.05
6.254e+002
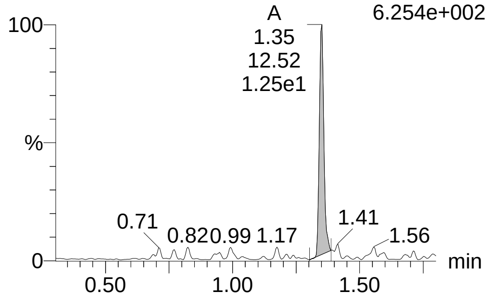

Sample Name: A MSM 6-2-3
A MSM 48 F1:MRM of 1 channel,ES+
419.868 > 295.05
5.480e+002
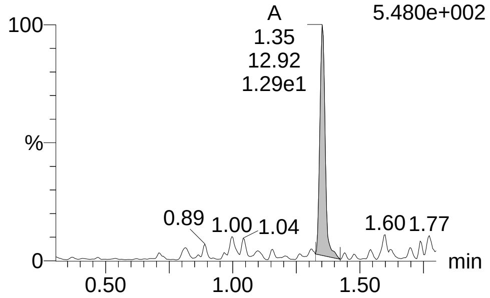

Sample Name: A MSM Blank 7
A MSM 49 F1:MRM of 1 channel,ES+
419.868 > 295.05
7.444e+001
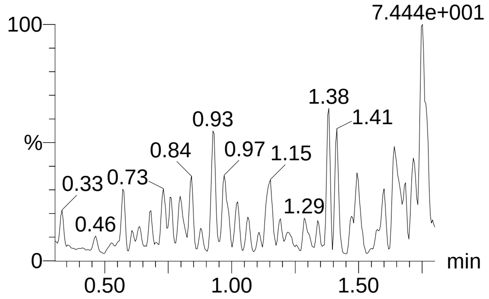

Sample Name: A MSM 24-1-1
A MSM 70 F1:MRM of 1 channel,ES+
419.868 > 295.05
1.678e+003
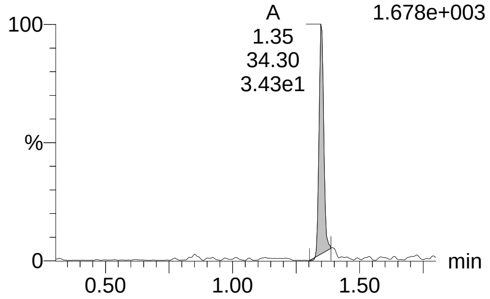

Sample Name: A MSM 24-1-2
A MSM 71 F1:MRM of 1 channel,ES+
419.868 > 295.05
1.790e+003
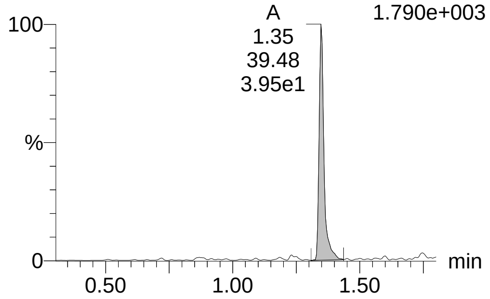

Sample Name: A MSM 24-1-3
A MSM 72 F1:MRM of 1 channel,ES+
419.868 > 295.05
1.915e+003
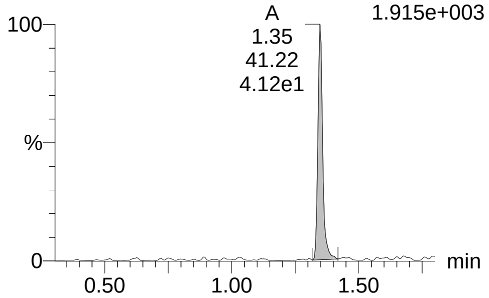

Sample Name: A MSM 24-2-1
A MSM 73 F1:MRM of 1 channel,ES+
419.868 > 295.05
5.873e+002
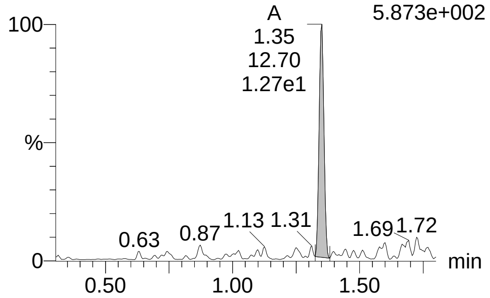

Sample Name: A MSM 24-2-2
A MSM 74 F1:MRM of 1 channel,ES+
419.868 > 295.05
6.819e+002
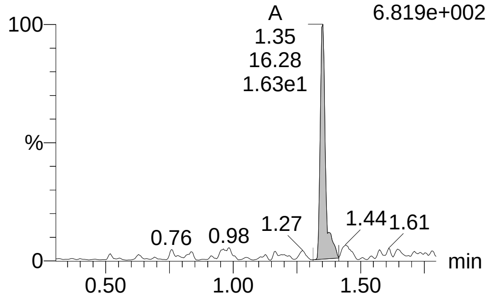

Sample Name: A MSM 24-2-3
A MSM 75 F1:MRM of 1 channel,ES+
419.868 > 295.05
7.848e+002
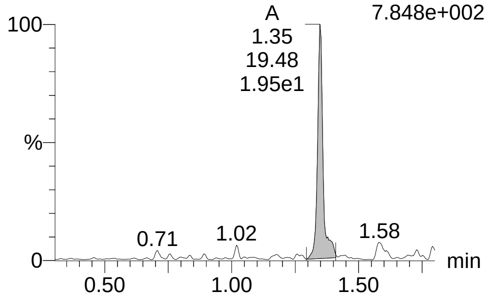

	#	Name	Type	Std. Conc	RT	Area	IS Area	Response	Detecti...	ng/mL	%Dev
1	1	A MSM 0-1-1	Anal...		1.35	1574.286		1574.286	bb		
2	2	A MSM 0-1-2	Anal...		1.35	1543.731		1543.731	bb		
3	3	A MSM 0-1-3	Anal...		1.35	1605.405		1605.405	bb		
4	4	A MSM 0-2-1	Anal...		1.35	1561.776		1561.776	bb		
5	5	A MSM 0-2-2	Anal...		1.35	1651.649		1651.649	MM		
6	6	A MSM 0-2-3	Anal...		1.35	1495.935		1495.935	bb		
7	7	A MSM Blank 1	Blank								
8	8	A MSM 15-1-1	Anal...		1.35	705.189		705.189	bb		
9	9	A MSM 15-1-2	Anal...		1.35	690.656		690.656	bb		
10	10	A MSM 15-1-3	Anal...		1.35	675.189		675.189	bb		
11	11	A MSM 15-2-1	Anal...		1.35	712.874		712.874	bb		
12	12	A MSM 15-2-2	Anal...		1.35	706.255		706.255	bb		
13	13	A MSM 15-2-3	Anal...		1.35	722.751		722.751	bb		
14	14	A MSM Blank 2	Blank								
15	15	A MSM 30-1-1	Anal...		1.35	308.876		308.876	bb		
16	16	A MSM 30-1-2	Anal...		1.35	322.889		322.889	bb		
17	17	A MSM 30-1-3	Anal...		1.35	318.783		318.783	MM		
18	18	A MSM 30-2-1	Anal...		1.35	372.798		372.798	MM		
19	19	A MSM 30-2-2	Anal...		1.35	309.781		309.781	bb		
20	20	A MSM 30-2-3	Anal...		1.35	341.714		341.714	bb		
21	21	A MSM Blank 3	Blank								
22	22	A MSM 1-1-1	Anal...		1.35	111.793		111.793	bb		
23	23	A MSM 1-1-2	Anal...		1.35	115.160		115.160	bb		
24	24	A MSM 1-1-3	Anal...		1.35	114.349		114.349	bb		
25	25	A MSM 1-2-1	Anal...		1.35	115.862		115.862	bb		
26	26	A MSM 1-2-2	Anal...		1.35	95.047		95.047	MM		
27	27	A MSM 1-2-3	Anal...		1.35	106.940		106.940	bb		
28	28	A MSM Blank 4	Blank								
29	29	A MSM 2-1-1	Anal...		1.35	30.357		30.357	MM		
30	30	A MSM 2-1-2	Anal...		1.35	29.151		29.151	MM		
31	31	A MSM 2-1-3	Anal...		1.35	28.388		28.388	MM		
32	32	A MSM 2-2-1	Anal...		1.35	32.411		32.411	MM		
33	33	A MSM 2-2-2	Anal...		1.35	29.102		29.102	MM		
34	34	A MSM 2-2-3	Anal...		1.35	28.537		28.537	MM		
35	35	A MSM Blank 5	Blank								
36	36	A MSM 4-1-1	Anal...		1.35	15.026		15.026	MM		
37	37	A MSM 4-1-2	Anal...		1.35	16.883		16.883	MM		
38	38	A MSM 4-1-3	Anal...		1.35	15.103		15.103	MM		
39	39	A MSM 4-2-1	Anal...		1.35	18.040		18.040	MM		
40	40	A MSM 4-2-2	Anal...		1.35	12.064		12.064	MM		
41	41	A MSM 4-2-3	Anal...		1.35	12.229		12.229	MM		
42	42	A MSM Blank 6	Blank								
43	43	A MSM 6-1-1	Anal...		1.35	24.249		24.249	MM		
44	44	A MSM 6-1-2	Anal...		1.35	23.711		23.711	MM		
45	45	A MSM 6-1-3	Anal...		1.35	24.281		24.281	MM		
46	46	A MSM 6-2-1	Anal...		1.35	12.707		12.707	MM		
47	47	A MSM 6-2-2	Anal...		1.35	12.524		12.524	MM		
48	48	A MSM 6-2-3	Anal...		1.35	12.917		12.917	MM		
49	49	A MSM Blank 7	Blank								
50	50	A MSM 24-1-1	Anal...		1.35	34.303		34.303	MM		
51	51	A MSM 24-1-2	Anal...		1.35	39.484		39.484	MM		
52	52	A MSM 24-1-3	Anal...		1.35	41.216		41.216	MM		
53	53	A MSM 24-2-1	Anal...		1.35	12.700		12.700	MM		
54	54	A MSM 24-2-2	Anal...		1.35	16.283		16.283	MM		
55	55	A MSM 24-2-3	Anal...		1.35	19.478		19.478	MM		

Method: C:\MassLynx_Projects\Medchem SU.PRO\MethDB\A.mdb 11 Jan 2021 10:59:51
Calibration: 11 Jan 2021 11:00:12

```
Header
Acquired File Name:   A MSM 0-1-1
Acquired Date:        07-Jan-2021
Acquired Time:        20:49:57
Job Code:             MSM 20210107 - 002 - 026 - 75 - A
Task Code:
User Name:
Laboratory Name:
Instrument:           ACQ-TQD#QBA320
Conditions:
Submitter:
SampleID:             A MSM 1
Bottle Number:        2:64
Description:
Instrument Calibration:
Calibration File: C:\MassLynx\IntelliStart\Results\Unit Mass Resolution\Calib 20161209.cal
Parameters
MS1 Static:
Mass:             20 Da to 1974 Da.
Resolution:       15.0/15.0
Ion Energy:       0.5
Reference File:   Naics2
Acquisition File: STATMS1
MS1 Scanning:
Mass:             15 Da to 2048 Da.
Resolution:       15.0/15.0
Ion Energy:       0.5
Reference File:   Naics2
Acquisition File: SCNMS1
MS1 Scan Speed Compensation:
Scan:             339 to 2000 amu/sec.
Resolution:       15.0/15.0
Ion Energy:       0.5
Reference File:   Naics2
Acquisition File: FASTMS1
MS2 Static:
Mass:             20 Da to 1974 Da.
Resolution:       15.0/15.0
Ion Energy:       0.5
Reference File:   Naics2
Acquisition File: STATMS2
MS2 Scanning:
Mass:             15 Da to 2048 Da.
Resolution:       15.0/15.0
Ion Energy:       0.5
Reference File:   Naics2
Acquisition File: SCNMS2
MS2 Scan Speed Compensation:
Scan:             339 to 10165 amu/sec.
Resolution:       15.0/15.0
Ion Energy:       0.5
Reference File:   Naics2
Acquisition File: FASTMS2
Calibration Time: 10:33
Calibration Date: 12/09/16
Coefficients
MS1 Static:       -0.000000000000*x^4 + 0.000000000843*x^3 + -0.000001360559*x^2 + 1.000940122634*x
 +-0.253266439024
MS2 Static:       -0.000000000000*x^4 + 0.000000000573*x^3 + -0.000001239648*x^2 + 1.000902600483*x
 +-0.192715155436
Function 1:       None
Function 2:       None
Parameters for C:\Documents and Settings\Administrator\Desktop\old desktop\log D\EA.EXP
Data Processing:
SIR / MRM Chromatogram Spike Removal      ON
```

```
SIR / MRM Smoothing           OFF
Smoothing window size (scans) 3
Number of smooths       2
Prescan Statistics:
Initial Average Intensity     22.1158
Initial Average Std Dev       1.8762
Bunch Zero Level        0.0036
Bunch Std Dev                 0.0096
Bunch Threshold               0.0768
Spike Removal Std Dev         1.7801
Method Events:
Initial Stop Flow:      No Change
Initial Switch 2: No Change
Initial Switch 3: No Change
Initial Switch 4: No Change
Initial Infusion: No Change
Initial Flow State:     LC
Initial Flow Rate:      5
Initial Reservoir:      No Action
API Probe Delay Temp:   20
Initial Refill:         No Action
Timed Events Enabled
Event Time  Name        Action
Instrument Parameters - Function 1:
Parameter File - C:\Documents and Settings\Administrator\Desktop\old desktop\log D\log D tune file.
IPR
Polarity    ES+
Calibration Static 2
Capillary (kV)    3.50  3.48
Cone (V)    38.00 35.41
Extractor (V)     3.00  2.20
RF (V)      0.10
Source Temperature (°C) 140   139
Desolvation Temperature (°C)  450   450
Cone Gas Flow (L/Hr)    90    90
Desolvation Gas Flow (L/Hr)   900   900
Collision Gas Flow (mL/Min)   0.20  0.20
LM 1 Resolution   15.50
HM 1 Resolution   14.84
Ion Energy 1      0.30
MS Mode Entrance  50.00
MS Mode Collision Energy      3.00
MS Mode Exit      50.00
MSMS Mode Entrance      -2.00
MSMS Mode Collision Energy    2.00
MSMS Mode Exit    2.00
LM 2 Resolution   15.00
HM 2 Resolution   15.50
Ion Energy 2      1.22
Gain  1.00
Multiplier  -493.30
Active Reservoir  B
Engineers Settings:
MS1 Low Mass Position         518
MS1 High Mass Position        284
MS1 Low Mass Resolution       513
MS1 High Mass Resolution      1732
MS1 Resolution Linearity      834
MS1 High Mass DC Balance      0
MS1 DC Polarity               Positive
MS2 Low Mass Position         519
MS2 High Mass Position        238
MS2 Low Mass Resolution       518
MS2 High Mass Resolution      644
MS2 Resolution Linearity      756
MS2 High Mass DC Balance      -0
MS2 DC Polarity               Positive
HM RF Lens Correction +       0
```

```
HM RF Lens Correction -       0
Inter-scan delays:
Automatic Mode
MS 1 Delay Table:
         R     delay
<=   0.500     0.005
<=   1.200     0.010
<=   2.400     0.015
<=   6.000     0.020
<=  15.000     0.025
<=  25.000     0.028
>   25.000     0.030
MS 2 Delay Table:
         R     delay
<=   2.000     0.005
<=   4.000     0.008
<=   7.000     0.010
<=  10.000     0.012
<=  20.000     0.014
>   20.000     0.016
ACE Experimental Record
Inlet Method File: c:\masslynx_projects\medchem su.pro\acqudb\adme sophie
-------------------   Prerun method parameters   ---------------
Waters ACQUITY QSM
Waters Acquity TUV
 Run Time: 0.20 min
 Wavelength Mode: Single Wavelength
 Lamp On: On
Channel A...
 Comment:
 Wavelength: 254 nm
 Sampling Rate: 20 points/sec
 Data Mode: Absorbance
 Time Constant: 0.1000 sec
 Auto Zero On Wavelength Change: Maintain Baseline
 Auto Zero On Inject Start: Yes
Analog 1...
 Sensitivity: 2.000 AUFS
 Chart Polarity: Positive (+)
 Voltage Offset: 0 mV
 Enable Chart Mark: Yes
 Run Events: Yes
 Pulse Width: 1.0 sec
 Rect Wave Period: 0.2 sec
----------------------------  oOo  ---------------------------
---------------------   Run method parameters   ----------------
Waters ACQUITY QSM
 Solvent A Name: Water
 Solvent B Name: Acetonitrile
 Solvent C Name: water 1 % FA
 Solvent D Name:
 Low Pressure Limit: 0 psi
 High Pressure Limit: 15000 psi
 Seal Wash Period: 5.00 min
 [Gradient Table]
  Time(min) Flow Rate(mL/min) %A %B %C %D Curve
 1. Initial 0.700 90.0 5.0 5.0 0.0 Initial
 2. 0.15 0.700 45.0 50.0 5.0 0.0 6
 3. 1.50 0.700 0.0 95.0 5.0 0.0 6
 4. 1.80 0.700 90.0 5.0 5.0 0.0 1
 Comment: ACQUITY UPLC BEH C18 2.1 x 50 mm
 Flow Ramp Rate: 0.45 min
 D Solvent Selection (if supported): No Change
 System Pressure Data Channel: No
 Flow Rate Data Channel: No
 %A Data Channel: No
 %B Data Channel: No
 %C Data Channel: No
```

```
 %D Data Channel: No
 Primary Data Channel: No
 Accumulator Data Channel: No
 Degasser Data Channel: No
 Gradient Start: At Injection
 Gradient Start Volume: 0 uL
 Gradient Start Time: 0.00 min
 Participate in pre-analysis: No
Waters Acquity TUV
 Run Time: 1.80 min
 Wavelength Mode: Single Wavelength
 Lamp On: On
Channel A...
 Comment:
 Wavelength: 214 nm
 Sampling Rate: 20 points/sec
 Data Mode: Absorbance
 Time Constant: 0.1000 sec
 Auto Zero On Wavelength Change: Maintain Baseline
 Auto Zero On Inject Start: Yes
Analog 1...
 Sensitivity: 2.000 AUFS
 Chart Polarity: Positive (+)
 Voltage Offset: 0 mV
 Enable Chart Mark: Yes
 Run Events: Yes
 Pulse Width: 1.0 sec
 Rect Wave Period: 0.2 sec
Waters ACQUITY FTN AutoSampler
 Run Time: 1.80 min
 Comment: ACQUITY UPLC BEH C18 2.1 x 50 mm
 Load Ahead: Disabled
 Loop Offline: Automatic min
 Wash Solvent Name: Acetonitrile
 Pre-Inject Wash Time: 0.0 sec
 Post-Inject Wash Time: 6.0 sec
 Purge Solvent Name: Water
 Dilution: Disabled
 Dilution Volume: 0 uL
 Delay Time: 0 min
 Dilution Needle Placement: 4.0 mm
 Target Column Temperature: 40.0 C
 Column Temperature Alarm Band: Disabled
 Target Sample Temperature: 15.0 C
 Sample Temperature Alarm Band: Disabled
 Syringe Draw Rate: Automatic
 Needle Placement: Automatic
 Pre-Aspirate Air Gap: Automatic
 Post-Aspirate Air Gap: Automatic
 Column Temperature Data Channel: No
 Room Temperature Data Channel: No
 Sample Temperature Data Channel: No
 Sample Organizer Temperature Data Channel: No
 Sample Pressure Data Channel: No
 Preheater Temperature Data Channel: No
 Seal Force Data Channel: No
 No Injection Mode Enabled: No
 Run Events: No
Sample Run Injection Parameter
Injection Volume (ul)   -   3.00
----------------------------  oOo  -----------------------------
End of experimental record.
-------------------- Waters ACQUITY QSM Postrun Report  ---------------
 Firmware Version: 1.50.237 (May 18 2011)
 Software Version: 1.50.1621
 Checksum: 0xae400516
 Serial Number: M09QSM056N
 Minimum System Pressure: 0.0 psi
```

```
 Maximum System Pressure: 0.0 psi
 Average System Pressure: 0.0 psi
----------------------------  oOo  ----------------------------
------------------- Waters ACQUITY FTN Postrun Report  ---------------
 Software Version: 1.50.1481
 Firmware Version: 1.50.317 (Jul 11 2011)
 Checksum: 0x3e83519d
 Serial Number: M09SDI055N
 Sample Syringe Size: 100.0
 Extension Loop Size: 0.0
 Needle Size: 15.0
 Column Type: ACQUITY UPLC® BEH C18 1.7µm
 Column Serial Number: 02343407715781
 Total Injections on Column: 456
 Minimum Sample Temperature: 0.0
 Maximum Sample Temperature: 0.0
 Average Sample Temperature: 0.0
 Minimum Column Temperature: 30.0
 Maximum Column Temperature: 41.4
 Average Column Temperature: 0.0
----------------------------  oOo  ----------------------------
------------------- Generic Instrument Postrun Report  ---------------
 Software Version: 1.50.2530
 Firmware Version: 1.50.2182 (May 11 2011)
 Checksum: 0xc09b9cb2
 Serial Number: J08UPT460M
 Lamp On/Off Event: No
 Lamp Life: 39.00
 Lamp Serial Number: 000296721
 Flow Cell Type: Other
 Flow Cell Path Length: 0.00 mm
 Flow Cell Volume: 0.00 microliters
 Flow Cell Serial Number: 1
 Flow Cell Part Number: 1
 Optics Temperature Stabilzation Setting: unknown
----------------------------  oOo  ----------------------------
------------------- Waters ACQUITY QSM Postrun Report  ---------------
 Firmware Version: 1.50.237 (May 18 2011)
 Software Version: 1.50.1621
 Checksum: 0xae400516
 Serial Number: M09QSM056N
 Minimum System Pressure: 0.0 psi
 Maximum System Pressure: 0.0 psi
 Average System Pressure: 0.0 psi
----------------------------  oOo  ----------------------------
Function 1
Scans in function:          338
Cycle time (secs):          Automatic
Inter Scan Delay (secs):    Automatic
Inter Channel Delay (secs):Automatic
Span (Da):                  2.000
Start and End Time(mins):    0.300 to 1.800
Ionization mode:            ES+
Data type:                  SIR or MRM data
Function type:              MRM of 1 channel
Chan Reaction               Dwell(secs) Cone Volt. Col.Energy Delay(secs) Compound Formula|Mass Comm
ents
1  : 419.87 > 295.05           0.260       30.0       18.0       Auto     EA        418.9        Inte
lliStart Generated
Function 2
Scans in function:          2161
Function type:              Diode Array
Wavelength range (nm):       214 to 214
```
