# Supplementary material for: Small-molecule-induced ERBB4 activation to treat heart failure
Source: Nat Commun. 2025 Jan 10;16:576. doi: 10.1038/s41467-024-54908-5 (PMC11724075; doi:10.1038/s41467-024-54908-5)
Supplement: Supplementary file 5 — Reporting Summary [file 41467_2024_54908_MOESM5_ESM.pdf]

nature portfolio

Corresponding author(s): Vincent F. Segers

Last updated by author(s): Nov 18, 2024

Reporting Summary

Nature Portfolio wishes to improve the reproducibility of the work that we publish. This form provides structure for consistency and transparency in reporting. For further information on Nature Portfolio policies, see our Editorial Policies and the Editorial Policy Checklist.

Statistics

For all statistical analyses, confirm that the following items are present in the figure legend, table legend, main text, or Methods section.

n/a	Confirmed	
☐	☒	The exact sample size (*n*) for each experimental group/condition, given as a discrete number and unit of measurement
☐	☒	A statement on whether measurements were taken from distinct samples or whether the same sample was measured repeatedly
☐	☒	The statistical test(s) used AND whether they are one- or two-sided<br>*Only common tests should be described solely by name; describe more complex techniques in the Methods section.*
☒	☐	A description of all covariates tested
☐	☒	A description of any assumptions or corrections, such as tests of normality and adjustment for multiple comparisons
☐	☒	A full description of the statistical parameters including central tendency (e.g. means) or other basic estimates (e.g. regression coefficient) AND variation (e.g. standard deviation) or associated estimates of uncertainty (e.g. confidence intervals)
☐	☒	For null hypothesis testing, the test statistic (e.g. *F*, *t*, *r*) with confidence intervals, effect sizes, degrees of freedom and *P* value noted<br>*Give P values as exact values whenever suitable.*
☒	☐	For Bayesian analysis, information on the choice of priors and Markov chain Monte Carlo settings
☒	☐	For hierarchical and complex designs, identification of the appropriate level for tests and full reporting of outcomes
☒	☐	Estimates of effect sizes (e.g. Cohen's *d*, Pearson's *r*), indicating how they were calculated

Our web collection on statistics for biologists contains articles on many of the points above.

Software and code

Policy information about availability of computer code

Data collection

- Luminescence was measured using the EnVision plate reader and the Luminoskan Ascent reader.
- Flow cytometry was used for the fluorescent competition binding assay.
- For RT-qPCR data collection, we utilized QuantStudio 3 as instrument.
- Data for experiments with optical densities were measured on the Microplate Epoch Reader with the right wavelength.
- Liquid chromatography with tandem mass spectrometry (Waters Acquity H-class UPLC system with a Bruker Daltonics Esquire 3000 plus ion trap mass spectrometer and an Agilent 1100 Series LC system) was used to collect data about the compound stability in plasma and liver microsomes.
- Echocardiography was performed using the Vevo F2-LAZRX and UHF57x probe.
- We used Olympus BX43 (Stream Motion Software) and CELENA® S Digital Imaging system for acquiring images of hearts and cell culture samples.
- For western Blot, images were visualized with the Odessey imaging system.

Data analysis

- Spotfire (TIBCO) was used for data analysis and visualization of the high-throughput screening. Data from the primary screening was analyzed via the HTS-Corrector software.
- RNA sequencing experiments, gene expression was quantified at the transcript level using Salmon (v1.10.0), with the validatMappings and -gcBias parameters switched on, to the Rnor_6.0 or GRCh38 transcriptome. Transcript level counts were aggregated to gene level using the import in the tximport package (v1.26.1), setting countsFromAbundance to 'lengthScaledTPM' in R (v4.1.1). DESeq2 R package (v1.38.3) was used for differential gene expression analysis between different conditions. The batch variability of different sequencing runs was accounted for by defining "batch" as a covariate in the linear model to analyse differential gene expression. Differential gene expression heat-maps were generated by using the pheatmap R package (v1.0.12), and volcano plots by EnhancedVolcano (v1.10.0). The overlapping genes between

different conditions were obtained by VennDiagram (v1.7.3). Functional enrichment of DEGs was determined using a hypergeometric test against the Gene Ontology database by using the ClueGO (v2.5.7) module of Cytoscape (v3.9.1) with Benjamini–Hochberg adjusted (FDR) P < 0.05. GSEA Preranked method was performed to identify the Hallmark pathways.
- MassLynx was used for analysis of the LC-MS/MS data.
- For image analysis, ImageJ 2.14.0 software was employed for quantifying stained tissue sections.
- For the determination of cross-sectional area of cardiomyocytes in fluorescent pictures, we used doi.10.5281/zenodo.13309997 (version 1).
- Parasternal long-axis B-mode images and M-mode images were obtained and analyzed using VevoLAB software (FUJIFILM VisualSonics, Version 5.7.1).
- Western blot analysis was done with Image Lab software.
- Microsoft Excel files were used to organize all data values and GraphPad Prism 10 was utilized for all statistical analysis.

For manuscripts utilizing custom algorithms or software that are central to the research but not yet described in published literature, software must be made available to editors and reviewers. We strongly encourage code deposition in a community repository (e.g. GitHub). See the Nature Portfolio guidelines for submitting code & software for further information.

Data

Policy information about availability of data

All manuscripts must include a data availability statement. This statement should provide the following information, where applicable:

- Accession codes, unique identifiers, or web links for publicly available datasets
- A description of any restrictions on data availability
- For clinical datasets or third party data, please ensure that the statement adheres to our policy

All data supporting the findings of this study are available within the paper and its Supplementary Information. Source data are provided with this paper.
The RNA sequencing data generated has been deposited in NCBI's Gene Expression Omnibus and is accessible through GEO Series accession numbers GSE256024 for iAM data (https://www.ncbi.nlm.nih.gov/geo/query/acc.cgi?acc=GSE256024) and GSE261219 for HCF data (https://www.ncbi.nlm.nih.gov/geo/query/acc.cgi?acc=GSE261219).
For some Figures, BioRender.com was used. The license can be found as Supplementary Information.
The code for the determination of the cross-sectional area of cardiomyocytes from fluorescence images and its analysis associated with the current submission is available at https://doi.org/10.5281/zenodo.13309997. Any updates will also be published on Zenodo, and the final DOI cited in the manuscript.
All mass spectrometry data are available as Supplementary File.

Research involving human participants, their data, or biological material

Policy information about studies with human participants or human data. See also policy information about sex, gender (identity/presentation), and sexual orientation and race, ethnicity and racism.

Reporting on sex and gender	Human participants were not used in this study.
Reporting on race, ethnicity, or other socially relevant groupings	Human participants were not used in this study.
Population characteristics	Human participants were not used in this study.
Recruitment	Human participants were not used in this study.
Ethics oversight	Human participants were not used in this study.

Note that full information on the approval of the study protocol must also be provided in the manuscript.

Field-specific reporting

Please select the one below that is the best fit for your research. If you are not sure, read the appropriate sections before making your selection.

☒ Life sciences ☐ Behavioural & social sciences ☐ Ecological, evolutionary & environmental sciences

For a reference copy of the document with all sections, see nature.com/documents/nr-reporting-summary-flat.pdf

Life sciences study design

All studies must disclose on these points even when the disclosure is negative.

Sample size	Sample size was determined using a power analysis based on preliminary data or prior studies to ensure sufficient power to detect statistically significant differences between experimental groups. Assuming an expected effect size, a standard deviation estimate, and a desired power of 80% with a significance level (α) of 0.05, we calculated the minimum number of samples and animals required per group. This method ensures that the study is adequately powered to minimize the risk of Type II errors, while avoiding unnecessary use of animals. All calculations were performed using standard statistical software.
Data exclusions	Data exclusions were made based on predefined criteria established before the start of the experiments to ensure data accuracy and relevance. Any samples or animals were excluded if they failed quality control thresholds, such as incomplete data, assay failures or technical issues such as poor quality staining, improper injection, or inconsistent echocardiographic measurements that could not be attributed to the

experimental intervention. Additionally, outliers were identified using a Grubbs' test for significant deviation from the group mean and were excluded where justified. For the MI experiment, cardiac ultrasound was performed two days after LAD ligation to exclude mice without successful MI (i.e. displaying hypokinesia in ≥ 2 out of 5 segments). Any data arising from non-compliance with experimental protocols were removed to maintain consistency across samples and uphold the study's integrity. All exclusions were applied uniformly across experimental groups to prevent bias, and no post-hoc exclusions were made based on experimental outcomes. These criteria were designed to minimize noise and enhance the reliability of the findings, ensuring robust and interpretable results.

Replication

To ensure the reproducibility of our experimental findings, we implemented several key measures. All experiments were performed in biological triplicates or more, with independent repeats conducted on separate days and with different cell culture passages to account for variability. Key findings were replicated across different batches of animals or cell cultures to confirm consistency. Additionally, standardized protocols were followed rigorously, and multiple investigators were involved in data collection and analysis to minimize bias. The data were also analyzed using consistent statistical methods across replicates to verify the robustness of the results.

Randomization

Samples and animals were randomly allocated into experimental groups to minimize selection bias. Randomization was performed using Microsoft Excel to assign samples and animals to each group in a manner that ensured balanced distribution. Additionally, for experiments involving multiple treatment conditions, investigators were blinded to group assignments during the intervention and data collection phases.

Blinding

Investigators were blinded to group allocation during both data collection and analysis to minimize bias. Blinding was achieved by coding the samples or by ear punching of the animals that concealed their group assignments. The investigators responsible for measuring outcomes, such as histological assessments, functional measurements, or molecular analyses, were unaware of the treatment conditions throughout the experimental procedures. Data analysis was conducted using these codes, and group identities were only revealed after the completion of all data collection and statistical analyses, ensuring objective assessment of the results.

Reporting for specific materials, systems and methods

We require information from authors about some types of materials, experimental systems and methods used in many studies. Here, indicate whether each material, system or method listed is relevant to your study. If you are not sure if a list item applies to your research, read the appropriate section before selecting a response.

Materials & experimental systems

n/a	Involved in the study	
☐	☒	Antibodies
☐	☒	Eukaryotic cell lines
☒	☐	Palaeontology and archaeology
☐	☒	Animals and other organisms
☒	☐	Clinical data
☒	☐	Dual use research of concern
☒	☐	Plants

Methods

n/a	Involved in the study	
☒	☐	ChIP-seq
☒	☐	Flow cytometry
☒	☐	MRI-based neuroimaging

Antibodies

Antibodies used

GAPDH // Mouse // Cell Signaling Technology // 97166 // Primary monoclonal Ab D4C6R // LOT 7 // 1:2,500 dilution
pAKT (Ser473) // mouse // Cell Signaling Technology// 23430 // Primary monoclonal Ab E4U3U// LOT 1 // 1:1,000 dilution
AKT // rabbit // Cell Signaling Technology// 9272 // Primary polyclonal Ab // LOT 30 // 1:1,000 dilution
pERK1/2 (Thr202/Tyr204) // rabbit // Cell Signaling Technology// 9101 // Primary polyclonal Ab // LOT 31 // 1:1,000 dilution
ERK1/2 // rabbit // Cell Signaling Technology// 9102 // Primary polyclonal Ab // LOT 20 // 1:1,000 dilution
ERBB4 // rabbit // Cell Signaling Technology // 4795 // Primary monoclonal Ab 111B2 // LOT 7 // 1:250 dilution
Anti-mouse 680 RD // Goat // Li-Cor // 926-68070 // Secondary Ab // LOT C90910-21 // 1:10,000 dilution
Anti-rabbit 800 RD // Goat // Li-Cor // 926-32211 // Secondary Ab // LOT D20208-05 // 1:10,000 dilution

Validation

Manufacturer information statements were reviewed on their respective websites, confirming the antibody's suitability for the species and experimental applications (Western blot). Each primary antibody used in this study was validated for species specificity (human and rat cell lines, mouse samples) and their dilution by western blotting. pAkt, Akt, pERK1/2 and ERK1/2 was validated in iAM cells with NRG1 15 minute stimulation as a positive control, according to the manufacturer's website. Regarding GAPDH and ERBB4, we performed an extra validation step because we needed to dilute GAPDH more and ERBB4 less than stated on the manufacturer's website. All relevant validation data are referenced to publications on the manufacturer's website to ensure robust and reliable antibody performance in our experiments.

Eukaryotic cell lines

Policy information about cell lines and Sex and Gender in Research

Cell line source(s)

PathHunter U2OS ERBB4/ERBB4 – osteosarcoma (Eurofins, 93-0961C3)
PathHunter U2OS ERBB2/ERBB4 – osteosarcoma (Eurofins, 493-0960C3)
PathHunter U2OS ERBB2/ERBB3 – osteosarcoma (Eurofins, 93-1042C3)
PathHunter U2OS ERBB1/ERBB1 – osteosarcoma (Eurofins, 93-0989C3)
Human cardiac fibroblasts – heart (human; Innoprot, P10454).

	Immortalized atrial myocytes – heart (rat), gift from LUMC
Authentication	Cell morphology was routinely monitored under light microscopy. Deviations from expected cell shape, size, or growth patterns were flagged for further investigation (not applicable in our study). Cell lines were cultured according to standard protocols, and passage numbers were closely tracked to avoid genetic drift or senescence. Cells were not used beyond recommended passage limits to maintain the integrity of the experimental data.
Mycoplasma contamination	All cell lines tested negative for mycoplasma contamination.
Commonly misidentified lines (See ICLAC register)	Commonly misidentified cell lines were not used in this study.

Animals and other research organisms

Policy information about studies involving animals; ARRIVE guidelines recommended for reporting animal research, and Sex and Gender in Research

Laboratory animals	Thirteen-week-old C57BL/6N (Charles River, 027) female and male mice // Twelve-week-old female and male Balb/cJ (Charles River, 000651) mice // Eleven-week-old female and male iCAGGCre-ERTM/Erbb4f/f mice [breeding combination of floxed Erbb4 mice (Erbb4f/f; B6; 129-Erbb4tm1Fej/Mmucd, MMRRC, #010439-UCD) were crossed with CAGGCre-ERTM mice (Jackson Laboratory, 004682) containing the Tg(CAG-cre/Esr1*)5Amc transgene].
Wild animals	The study did not involve wild animals.
Reporting on sex	Sex was considered in conducted experiments in our study design. Both male and female mice were used in a model of myocardial fibrosis, acute cardiotoxicity and myocardial infarction, to account for potential sex-based differences in response to the experimental interventions. Randomized allocation of sex was applied across experimental groups, ensuring equal distribution of male and female subjects where applicable. Data were collected separately for each sex and disaggregated accordingly in the source data. Analysis and description of the results was always shown and reported separately for males and females, except for the Erbb4-null mice (because no sex-based differences were observed). Overall, 172 male mice and 152 female mice were used in this study.
Field-collected samples	The study did not involve samples collected from the field.
Ethics oversight	All animal experiments were approved by the Ethical Committee of the University of Antwerp (approval number ECD 2020-08) and conformed to the Guide for the Care and Use of Laboratory Animals, 8th edition published by the US National Institutes of Health in 2011, and to the European Communities Council Directive 2010/63/EU for the protection of animals used for experimental purposes.

Note that full information on the approval of the study protocol must also be provided in the manuscript.

Plants

Seed stocks	*Report on the source of all seed stocks or other plant material used. If applicable, state the seed stock centre and catalogue number. If plant specimens were collected from the field, describe the collection location, date and sampling procedures.*
Novel plant genotypes	*Describe the methods by which all novel plant genotypes were produced. This includes those generated by transgenic approaches, gene editing, chemical/radiation-based mutagenesis and hybridization. For transgenic lines, describe the transformation method, the number of independent lines analyzed and the generation upon which experiments were performed. For gene-edited lines, describe the editor used, the endogenous sequence targeted for editing, the targeting guide RNA sequence (if applicable) and how the editor was applied.*
Authentication	*Describe any authentication procedures for each seed stock used or novel genotype generated. Describe any experiments used to assess the effect of a mutation and, where applicable, how potential secondary effects (e.g. second site T-DNA insertions, mosiacism, off-target gene editing) were examined.*
